# A meta-analysis on the structure of pulmonary rehabilitation maintenance programmes on COPD patients’ functional capacity

**DOI:** 10.1038/s41533-022-00302-x

**Published:** 2022-10-03

**Authors:** Liliana Silva, Tiago Maricoto, Patrício Costa, Joana Berger-Estilita, José Miguel Padilha

**Affiliations:** 1 Matosinhos Local Health Unit, Porto, Portugal; 2 CINTESIS – Center for Health Technology and Services Research, Porto, Portugal; 3 Aradas Health Center, Aveiro, Portugal; 4 Faculty of Health Sciences – University of Beira Interior, CACB - Clinical Academic Centre of Beiras, Covilhã, Portugal; 5 Life and Health Sciences Research Institute (ICVS), School of Medicine, University of Minho, Braga, Portugal; 6 ICVS/3B’s, PT Government Associate Laboratory, Braga, Guimarães, Portugal; 7 Faculty of Psychology and Education Sciences, University of Porto, Porto, Portugal; 8 Department of Anaesthesiology and Pain Medicine, Inselspital, Bern University Hospital, University of Bern, Bern, Switzerland; 9 Porto Nursing School, Porto, Portugal; 10 RISE@CINTESIS, Porto, Portugal

**Keywords:** Rehabilitation, Chronic obstructive pulmonary disease, Translational research, Rehabilitation

## Abstract

Pulmonary rehabilitation (PR) improves functional capacity, health-related quality of life (HRQoL) in COPD patients, and maintenance programmes are relevant in preserving those improvements. However, little is known about the structure of maintenance programmes after PR. We performed a systematic review and meta-analysis of experimental and quasi-experimental studies evaluating individuals with COPD admitted to a maintenance PR programme, delivered after an initial PR programme. We reported functional capacity evaluation (6-minute-walking-test), HRQoL, dyspnoea and symptom control. Searches were performed on the 11^th^ April 2021 using MEDLINE, Embase, EBSCO, CINAHL, Web of Science and Cochrane Library. We extracted summary-level data from trial publications and used a random-effects model, predicting that severe heterogeneity was detected. The protocol was registered in PROSPERO (CRD42021247724). Fifteen studies were included in the meta-analysis, with 1151 participants. Maintenance programmes were associated with a pooled mean increase of 27.08 meters in 6mWT (CI: 10.39 to 43.77; I^2^ = 93%; *p* < 0.0001), being better in supervised, long (>12 month) home-based programmes; and having a potential MD of -4.20 pts in SGRQ (CI: -4.49 to -3.91; I^2^ = 0%; *p* = 0.74). Regarding dyspnoea and exacerbations, we found a nonsignificant trend for improvement after maintenance PR programmes. Severe COPD patients showed smaller improvements in programmes up to a year. Overall, the strength of the underlying evidence was moderate. Despite limitations of risk of bias and heterogeneity, our results support that home-based, supervised, long-term maintenance PR programmes may significantly improve functional capacity in COPD patients and HRQoL.

## Introduction

Chronic Obstructive Pulmonary Disease (COPD) is a common, preventable and treatable disease, and it is the third cause of death worldwide^1^. COPD patients often present a decline in functional capacity (FC) over time and persistent symptoms such as dyspnoea, low exercise tolerance and fatigue, leading to poor health-related quality of life and an increased risk for exacerbations and death^1^.

Pulmonary Rehabilitation (PR) is one of the most cost-effective treatments for COPD^1^. Its standard core elements are physical exercise training, patient-directed education, smoking cessation support, disease self-management and behaviour change^1–4^. However, improvements in the functional capacity of COPD patients last less than one year^1,2,4^.

Functional capacity tends to decrease over time, mainly if the patient does not change his behaviour concerning physical activity^1^. While initial studies showed a difference at six months favouring supervised maintenance exercises^5–7^, the effect seems not to be sustained at 12 months. A more recent meta-analysis suggested that maintenance PR programmes might confer long-term efficacy (12 months) on functional capacity in patients with COPD^8^, with a mean difference in the six-minute walk test (6mWT) of 27 meters. The 6mWT is a predictor of morbidity and mortality^9^ among COPD patients, it is a simple clinical exercise test for the objective evaluation of functional exercise capacity improvement in COPD, which measures the distance a patient can quickly walk on a flat hard surface in 6 minutes.

It is well known that PR programmes include outpatient or inpatient (centre-based), community and home-based types. Most PR programmes are centre-based. Participants are prescribed personalized and structured programmes^3^, usually lasting eight to twelve weeks^1,2^. Still, such programmes vary significantly depending on healthcare systems and local policies^2,10^. According to the consensus of PR, to maintain the benefits of this intervention, health behaviour change is crucial^1,2,4^.

Malaguti et al. ^11^, in a recent Cochrane review that specifically included papers on supervised maintenance programs, states that these programmes have an impact on functional exercise capacity. Particularly, the optimal duration to achieve relevant benefits, the role of the supervision and setting of maintenance PR programmes is still unclear^12^. Thus, little is known about what components should constitute a maintenance programme^8^.

This systematic review and meta-analysis aimed to synthesise’ evidence of interventional studies, including maintenance PR programmes on COPD patients, evaluate its impact on functional capacity and other disease-related secondary outcomes, and determine the components of those programmes that lead to better long term outcomes.

## Methods

This systematic review and meta-analysis followed the Cochrane Handbook recommendations^13^. Our study protocol has been prospectively registered and published in the PROSPERO database, CRD42021247724.

### Search strategy and selection criteria

The primary outcome of this study was the functional capacity measured by the 6mWT, and we selected experimental and quasi-experimental studies evaluating individuals with COPD submitted to a maintenance PR programme, delivered after an initial PR programme and reporting an evaluation of functional capacity measured by the 6mWT^14,15^. Quasi-experimental studies were considered in this systematic review because we aimed to acknowledge the components of the intervention.

Secondary outcomes considered for inclusion were dyspnoea, measured by the modified Medical Research Council scale (mMRC);^16^ exacerbations, measured by hospital admissions during the maintenance rehabilitation programme;^17^ and health-related quality of life (HRQoL), measured by the St George’s Respiratory Questionnaire (SGRQ)^18^, the EuroQoL5^19^ or SF-36^20^.

We excluded non-interventional studies and studies without an initial PR programme. In addition, studies in which the initial PR programme included only physical exercise or only education were also excluded because they are not actual PR programmes according to the accepted definition of PR^2,4^. We did not exclude studies based on language.

The search was performed on the 11th April 2021 in the Scopus database (MEDLINE and Embase), EBSCO, the Cumulative Index to Nursing and Allied Health Literature [CINAHL] Complete, Web of Science and Cochrane Library. In addition, we reviewed the references of the included literature and correlated systematic reviews. See “Supplementary Information 1 – Search strategy”.

Two researchers (LS, TM) conducted the study selection independently, using the RYYAN QCRI app (available at: https://www.rayyan.ai/), and all disagreements were resolved by consensus or through consultation with a third investigator (MP) in the review team. Duplicated and irrelevant studies were rejected first by examining titles and abstracts. After that, full texts of potentially eligible studies were obtained and reviewed according to inclusion and exclusion criteria. When the full text was not available, we contacted the authors to obtain raw data, and if the authors did not answer in two weeks, the study was excluded from the selection.

### Data analysis

For selected articles, two researchers (LS and TM) extracted the following study characteristics: study design, country of study, year of publication, sample size and participant characteristics [such as age, sex and severity of COPD assessed by percentage (%) of predicted Forced Expiratory Volume in the 1st second (FEV_1_)], duration of the previous/initial PR, setting/design and duration of the maintenance PR programme, supervision and follow-up. Estimates of the association between the intervention parameters (PR programmes) and the study outcomes were measured as mean and standard deviation (SD) for all continuous outcomes. Missing SD was calculated as recommended in the 16.1.3.2 chapter of Cochrane Handbook for Systematic Reviews of Interventions^13^. Studies that do not report group means or MD were not included in the meta-analysis. Agreement between researchers was calculated with kappa statistics^13^.

The meta-analysis was conducted using RevMan software v.5.4^21^. Due to the expected heterogeneity, we applied a random-effect model. For continuous outcome measures, data are presented as the mean and SD, and the effect size was estimated by the mean difference (MD) with 95% confidence intervals (CI). Missing SDs were arithmetically calculated^13^. Studies not reporting group means or MD were not included in the meta-analysis. Data not accessed through meta-analyses was summarised and described in the text.

In addition, there was, as we expected, heterogeneity due to the lack of evidence concerning the structure of maintenance programmes. Therefore, we performed sensitivity (leave-one-out) and subgroup analyses defined as a priori. We compared the effectiveness of the interventions between the duration of the initial PR programme, the severity of COPD (assessed by % of predicted FEV_1_, type of professional supervision and setting, risk of bias and publication year^2,4,11^. The guidelines recommend that these programmes last between eight and twelve weeks, therefore, we wanted to evaluate if the effects of a maintenance PR program were different in a twelve, eight or less than eight weeks programme^2,4^

The duration of the maintenance program and supervision by a health professional are aspects that vary substantially between existing programs and settings. We also wanted to adjust for the severity of COPD, according to % of predicted FEV_1_, and, finally, if the studies published before the 2013 guidelines^2^ had different results. The methodological quality of the primary studies was also a reason for the analysis.

We used R^22–25^ to perform a mixed-effects meta-regression to analyze the continuous variables in consideration of the presence of “residual heterogeneity”^26^. In addition, subgroup analysis was performed for all the categorical variables.

### Assessment of the risk of bias

Two independent researchers (LS and TM) assessed the quality of the evidence for the collected outcomes of interest using the Grading of Recommendations, Assessment, Development and Evaluations (GRADE) system^13^. We appraised each study’s components, including selection, performance, detection, attrition, reporting, and other biases. For each study, either for the grading of each component and for the global study rating, we assigned categories of risk of bias: low, unclear, and high. The global grading involved taking an average of all individual components^13^. As blinding of allocation to patient and PR therapist was not possible, studies without any other source of bias were considered as being at the lowest risk possible for this type of intervention. We assessed publication bias with a funnel plot created in RevMan software v.5.4^21^.

### Reporting summary

Further information on research design is available in the Nature Research Reporting Summary linked to this article.

## Results

**Fig. 1:**
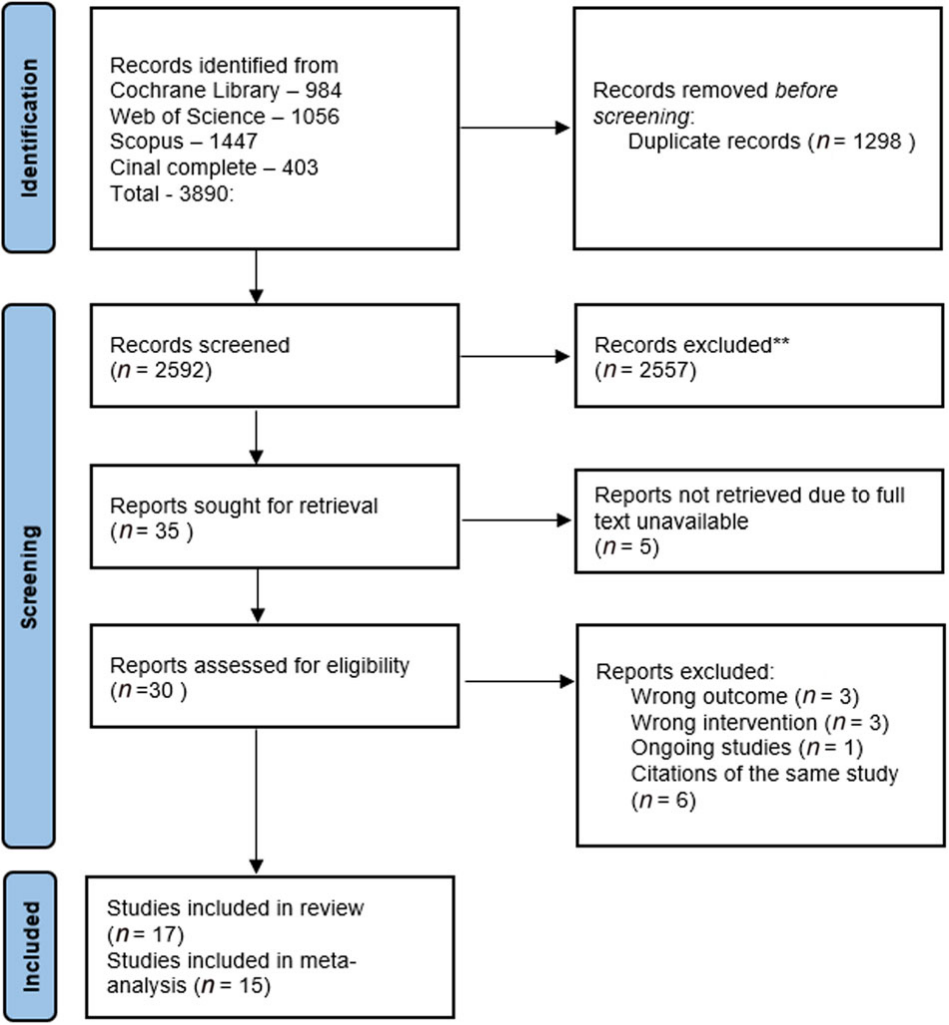
PRISMA Flowchart for Study selection.

The electronic database searches identified 3890 records, from which we removed 1298 duplicate records. From the remaining 2592 references, 2557 were excluded by title and abstract. This resulted in 35 records for the full-text review. After this, we excluded six records for failing to meet the inclusion criteria (three studies did not report an evaluation of functional capacity measured by the 6mWT. Two of them reported the endurance shuttle walk test^27,28^ and found no differences between groups in this test, and one study evaluated physical activity^29^. Three studies did not have an initial PR programme or the programme only included physical exercise, three were not a PR intervention, and one study was still ongoing^30^) (Fig. 1). We were unable to contact the authors of 5 records. Six citations reported duplicated data, referring to the same studies. Finally, 17 studies were kept after careful inspection. The *k* value was 0,85, indicating an almost perfect agreement^13^.

### Risk of bias in included studies

**Fig. 2:**
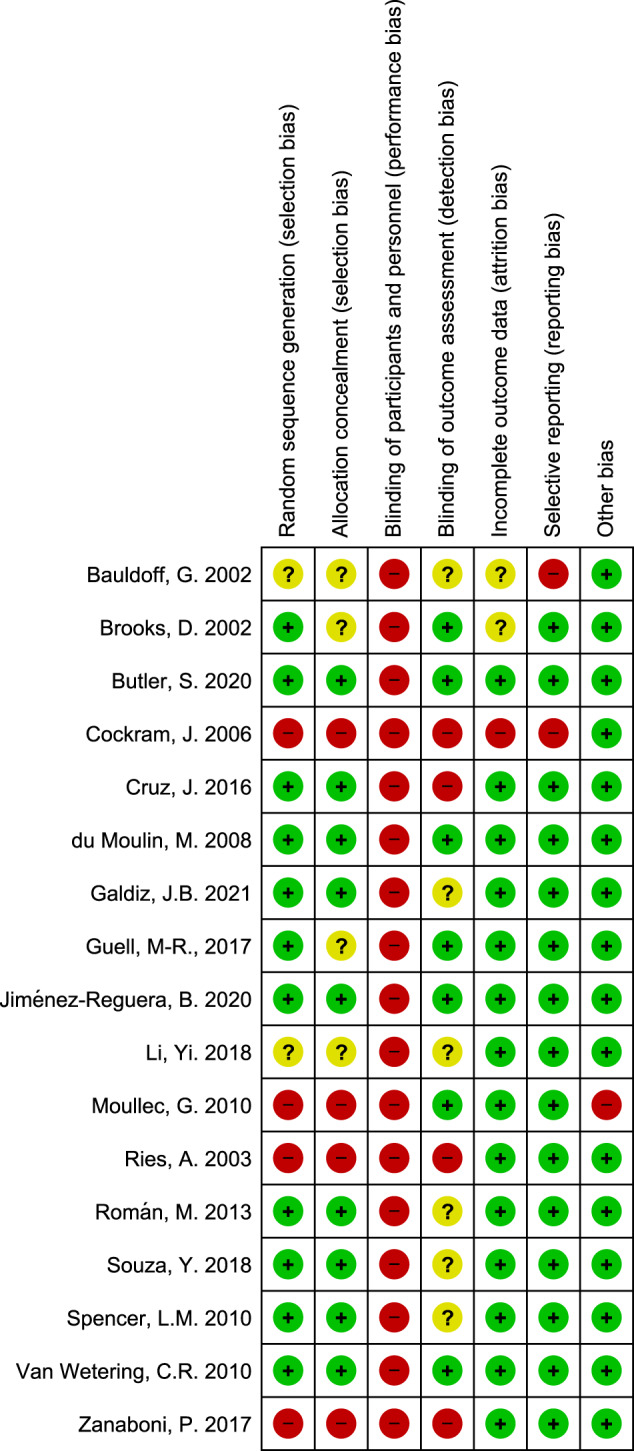
Detailed Risk of Bias assessment in included studies according to GRADE system.

The risk of bias full details may be found in “Supplementary Information 3 - Quality Assessment and Risk of Bias Table”. All studies had no blinding of participants and personnel, which was due to the nature of the intervention itself. Four studies presented a lack of blinding as the only source of bias, representing the best bias-free design possible for this kind of intervention. In addition, more than half of the included studies did not refer to the blinding of the outcome assessment, which is an important step that may overcome the difficulty upon blinding the intervention (Fig. 2).

The funnel plot analysis detected no relevant publication bias for all outcomes (see “Supplementary Information 4 – Complete meta-analysis data”).

### Description of studies

This review included 17 studies,14 of which were randomised controlled trials^31–44^, and 3 were quasi-experimental trial (two studies had no control group^45,46^, and one was not randomized^47^). Detailed information on included studies is available in “Supplementary Information 2 - Complete data of selected studies”.

The publication year varies from 2002^31,32^ to 2021^40^. The mean participants’ ages ranged between 62 and 69 years old, and the mean percentage of predicted FEV_1_ was between 34 and 66%.

The studies included 1222 participants, all receiving an initial PR programme, varying between three^39^ and twelve weeks^33,34,36,38^ duration. Among these, 1151 participants were randomly assigned to either a control or an intervention group. Forty participants were allocated to one of the groups, though not randomly^47^.

The intervention group had access to a maintenance PR programme, varying between 2^31^ and 36^36^ months. The presence of a healthcare provider supervision was variable (Table 1); in five studies, the intervention was always supervised^31,33,43,47^. In the other five, there was no supervision^32,34,39,40,42^, in six studies some of the sessions were supervised^35,37,38,41,44^.

**Table 1:** Description of included studies.

AUTHOR, YEAR	STUDY TYPE	SAMPLE SIZE	PRE PR PROGRAMME DURATION (WKS)	INTERVENTION	FOLLOW_UP (MONTHS)	SUPERVISION	SETTING	DISEASE SEVERITY
BAULDOFF, G.^31^	RCT	I: 12 C:12	Not reported	Use of music during exercise training and walks	2	SV	HB	NR
BROOKS, D.^32^	RCT	I: 37 C:48	inpatients: 6; outpatients: 8	Home programme and phone call between visits	12	USV	HB/CB	Moderate to Severe
BUTLER, S.J.^37^	RCT	I:49 C:48	Not reported	Community-based maintenance exercise programme and recommendations for home exercise.	12	AS	CB	Moderate to Severe
COCKRAM, J. 2006 ^45^	QE	230	8	Community classes for exercise trainning	12	SV	CB	NR
CRUZ, J.^38^	RCT	I: 16 C:16	12	Participants were given a piezoelectric pedometer, a log diary to record daily steps, and a calendar to register their short-term step-count goals and daily steps. Individual feedback is provided weekly (in the first month) and fortnightly (in the second and third months) by telephone calls. The final aim was to achieve the long-term goal.	3	AS	HB/CB	Mild to severe
MOULIN, M.^39^	RCT	I:10 C:10	3	Individualized training plan. Patients were instructed to quickly walk a distance equivalent to 125% of their last 6MWT three times a day, with each training walk not exceeding 15 min. Patients were given a pedometer so that the training could be better incorporated into daily activities in a homebased setting, and with a training diary. Telephone contacts for motivation.	6	USV	HB	NR
GALDIZ, J B.^40^	RCT	I:48 C:46	8	Three training sessions a week and four educational sessions	12	USV	Tele	Moderate to severe
GUELL, M-R.^41^	RCT	I:68 C:70	8	Home-based programme. Calls to patients every 15 days using a standardized protocol. Alternate week supervised training session.	36	AS	HB	Moderate to severe
JIMÉNEZ-REGUERA^42^	RCT	I: 20 C: 24	8	The HappyAir app has an educational programme and an integrated plan as a model of a therapeutic programme based on communication that introduced the figure of the therapeutic educator.	10	USV	Tele	Moderate to severe
LI, Y.^43^	RCT	I:65 C:69	8	Step 1: Home-visit once every 2 weeks for 2 months; Step 2: Home-visit every 4 weeks and phone contact once a week for 4 months; Step 3: Phone contact once a week for 6 months.	12	SV	HB	Mild to severe
MOULLEC, G.^47^	QE	I:14 C:26	4	Individualized exercise training (3.5 hours/week; 72 sessions) supervised by a teacher of adapted physical; health education provided alternatively by all professionals of the health care network (2 hours/month; 12 sessions) and psychosocial support with discussion group (1 hour/month; 12 sessions) supervised by a psychologist in the same room.	12	SV	CB	Moderate to severe
RIES, A.L.^44^	RCT	I:64 C:74	8	1-weekly telephone calls; 2-monthly supervised reinforcement sessions.	12	AS	HB/CB	Mild to severe
ROMÁN, M.^33^	RCT	I:26 C:22	12	Weekly-session maintenance programme.	12	SV	CB	Mild to severe
SPENCER, L.M.^35^	RCT	I:24 C:24	8	Supervised, outpatient-based exercise 1 day per week plus unsupervised home exercise on four other days.	12	AS	HB/CB	Moderate
SOUZA, Y.^34^	RCT	I:25 C:25	12	Patients received the manual for activities and were instructed to use it.	6	USV	HB	Mild to severe
WETERING^36^	RCT	I:102 C:97	12	Patients were instructed to perform the same exercises twice a day in their home environment Monthly encouragments to programme adherence and home training. Six extra training sessions in 3 weeks after exacerbations.	20	AS	HB	Moderate to severe
ZANABONI^46^	QE	10	4	Two-year telerehabilitation programme consisting of home exercise, telemonitoring and self-management, weekly supervised.	24	ASV	HB	Mild to severe

*RCT* Randomized controlled trial, *QE* quasi-experimental, *I* intervention, *C* control, *SV* supervised, *USV* unsupervised, *AS* Alt. supervision, *HB* home-based, *CB* community-based, *HB/CB* Alternate Home-based and community-based, *Tele* Telerehabilitation, *NR* not reported

In six studies, intervention was assessed through a home-based programme^31,34,39,41,43^, three took place in a community centre^33,37,47^, four studies used programmes that incorporated both home-based and community-based sessions^32,35,38,44^, and two studies assessed telerehabilitation^40,42^.

Five studies showed a significant improvement in functional capacity^31,35,36,43,47^ after the maintenance programme. In general, they used strategies for measuring programme adherence through daily exercise records performed by patients^36^. Within this group, three studies included home-based supervised programmes^31,36,43^.

In five studies^36,40,42,43,47^, education for self-management was provided. Only one study controlled the exercise intensity by the patient’s maximum heart rate and symptoms^43^. Pedometers were used in two studies^38,39^, and an experiment with music during exercise training and walks was explored in one study^31^.

One study^36^ included structured healthcare provider supervision regarding long-term programme transitions, and another study^43^ modified the intervention over time, reducing the number of sessions supervised in stages and alternating with telephone contacts.

### Meta-analysis and meta-regression

This review included 15 studies in the meta-analysis^31–44,47^, two of which were excluded^45,46^ because they lacked a control group. The two studies with more weight were Wetering et al. and Brooks et al. ^32,36^. We analysed the changes after the initial PR until the end of the maintenance and calculated the MD between these two moments.

### Primary outcome – Functional capacity measured with the 6mWT

**Fig. 3:**
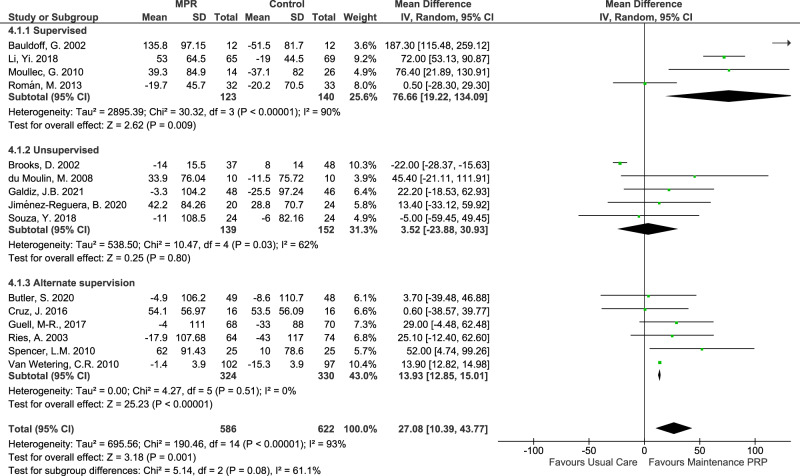
Forest plot of MD in 6mWT with maintenance PR programme, according to supervision type.

Most studies showed an effect favouring maintenance PR programmes over usual care, of which five studies showed high certainty evidence (Fig. 3). The pooled estimate for the maintenance group is a MD of 27 meters (CI: 10.4 to 43.8; I^2^ = 93%; *p* < 0.0001) (Fig. 3), which lies between the minimal important difference confidence interval that ranges between 25 and 33^14^.

Considering the severe heterogeneity detected across studies (I^2^ = 93%), we performed a sensitivity analysis with the leave-one-out method. Only one study showed an effect favouring usual care over maintenance PR programmes with high certainty evidence^32^. Although it revealed a slight reduction of heterogeneity to I^2^ = 81%, no significant changes were found in the pooled estimate. Supplementary Information 4 summarises the main findings of clinical outcomes from selected studies.

Nine studies^33,34,37–42,44^ showed low certainty evidence of the effect, in which 4^39–41,44^ the effect favoured the maintenance PR group over usual care. These studies included interventions with health technologies and telerehabilitation and were mainly unsupervised.

Subgroup and sensitivity analyses are available in detail in “Supplementary Information 4 - Complete meta-analysis data”.

The sub-analysis according to maintenance PR programme duration found a difference in the 6mWT favouring maintenance compared to the usual care group in programmes with more than 12 months^36,41^ with a MD of 14 meters with high certainty evidence and a significative reduction of heterogeneity (CI 12.8 to 15.0; I^2^ = 0%; *p* < 0.38). However, although programmes with eight weeks favoured maintenance PR programmes over usual care, they had high heterogeneity. Shorter programmes had low certainty evidence and high heterogeneity, although they also favoured maintenance PR programmes over usual care.

Subgroup analysis according to the initial PR programme duration revealed a difference in the 6mWT favouring maintenance compared to the usual care in studies with the initial PR programme of 12 weeks^33,34,36,38^ with high certainty evidence and a significative reduction of heterogeneity (MD of 14 meters; CI 12.8 to 15.0; I^2^ = 0%; *p* = 0.63). Studies with eight weeks of initial PR programmes^35,40–44^ also revealed a difference in the 6mWT favouring maintenance compared to the usual care (MD of 38 meters; CI 15.9 to 61.6; I^2^ = 60%; *p* = 0.03) but with marked heterogeneity across studies. One study^32^ has both inpatients in a 6-week PR programme and outpatients in an 8-week programme. Two studies^31,37^ have no data about the duration of the PR initial programme, and we excluded them from this subanalysis.

Regarding supervision by healthcare professionals, subgroup analysis revealed a difference in the 6mWT favouring maintenance compared to the usual care in studies with supervised intervention with high certainty evidence, although there was no significant reduction of heterogeneity (MD of 77 meters; CI 19.2 to 134.1.0; I^2^ = 90%; *p* < 0.00001). Programmes in which some of the sessions were supervised and others were unsupervised^35–38,41,44^ the results in the 6mWT favoured maintenance PR programmes compared to the usual care, with high certainty evidence and no heterogeneity detected across studies. (MD of 14 meters; CI 12.8 to 15.0; I^2^ = 0%; *p* = 0.51).

Considering COPD severity, studies that include people with all degrees of severity^32,34,37,40,41,43,47^ the results in the 6mWT favoured maintenance PR programmes compared to the usual care, with high certainty evidence and no heterogeneity detected across studies. (MD of 14 meters; CI 12.8 to 15.0; I^2^ = 0%; *p* = 0.44). Notwithstanding, although studies that enrolled moderate to severe COPD patients also favoured maintenance PR programmes compared to the usual care, they had low certainty evidence and high heterogeneity.

**Fig. 4:**
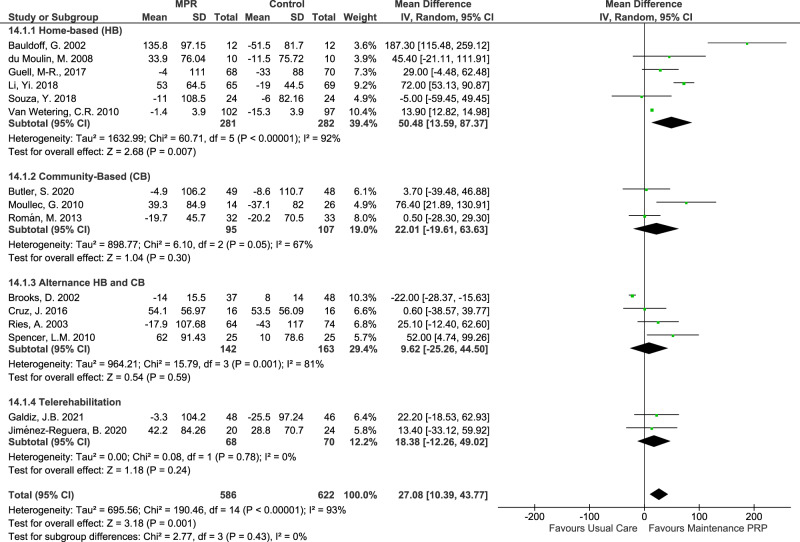
Forest plot of MD in 6mWT with maintenance PR programme, according to the setting.

According to the setting of the maintenance PR programme (Fig. 4), home-based maintenance programmes^31,34,36,39,41,43^, the 6mWT favoured maintenance PR programmes compared to the usual care, with high certainty evidence but significant heterogeneity detected across studies. (MD of 51 meters; CI 13.6 to 87.4; I^2^ = 92%; *p* < 0.00001). Even though other settings, such as community-based, telerehabilitation or the combination between home and community-based, favoured maintenance PR programmes compared to the usual care, they had low certainty evidence and relevant heterogeneity.

According to the studies’ risk of bias subgroup analysis, studies with lower risk^36,37,39,42^ showed results in the 6mWT favouring maintenance PR programmes compared to the usual care, with high certainty evidence and a significant reduction of the detected heterogeneity across studies (MD of 14 meters; CI 12.8 to 15.0; I^2^ = 0%; *p* = 0.78).

Subgroup analysis according to publication year revealed no relevant findings.

**Fig. 5:**
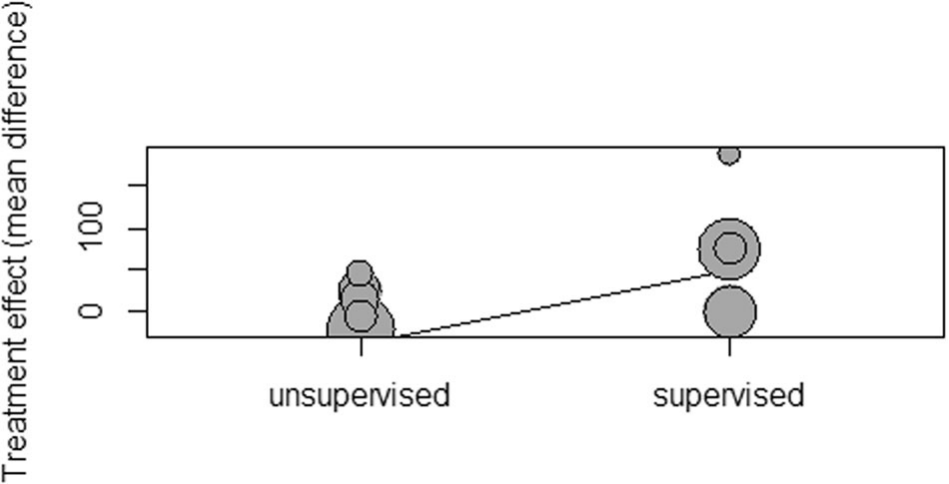
Bubble plot for supervision meta-regression adjusted for supervision, setting and programme duration.

In meta-regression, as we observe in Fig. 5, supervision, setting, and programme duration are heterogeneity moderators (*p* = 0.05), and, when adjusted to programme duration and setting, supervised programmes have a mean increment of 89 meters in MD between groups when compared to unsupervised programmes (*p* < 0.001). In the same metaregression, home-based programmes have a mean increment of 57 meters between groups compared to centre-based programmes (*p* < 0.01).

The meta-regression has shown that when adjusting to the setting and duration of the maintenance PR programme, supervised programmes when compared to unsupervised programmes, had a mean increment of 89 meters in MD between groups (*p* < 0.001), and home-based programmes had a mean increment of 57 meters (*p* < 0.01) in MD between groups when adjusted to both supervision and duration of the maintenance PR programme. Furthermore, when adjusting to the severity of the disease using the FEV_1_, supervised PR programmes had a mean increment of 73 meters (*p* < 0,01) compared to unsupervised programmes.

### Secondary outcome - Health-related quality of life

Among the primary studies that evaluated the HRQoL, we found two studies^42,46^ using the EQ5D and two others using the SF-36 form^40,41^ to monitor the HRQoL. None revealed significant changes in HRQoL between groups. In addition, four studies evaluated HRQoL with the SGRQ^35,36,38,42^.

**Fig. 6:**
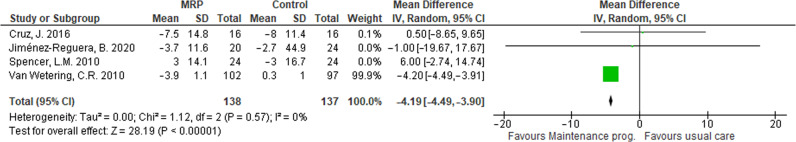
Forest plot of MD in HRQoL with a maintenance PR programme.

Overall, the studies showed little to no difference between maintenance and usual care groups concerning the SGRQ, and high heterogeneity was detected. We performed a sensitivity analysis using the leave one out method. Therefore, we exclude the studies on the risk of bias. (See Supplementary Information 4) After removing Spencer et al. ^35^, heterogeneity is not detectable, and the SGRQ results in favour maintenance PR programmes compared to the usual care, with high certainty evidence and a significant reduction of the detected heterogeneity across studies (MD of −4 pts; CI −4.5 to −3.9; I^2^ = 0%; *p* = 0.57) (Fig. 6). The same trend was found when considering only studies with a low risk of bias^36,42^ (MD −4 pts; CI −4.5 to −3.9; I^2^ = 0%; *p* = 0.74), achieving the minimal important change to general COPD patients of four points^18^.

### Secondary outcome - Dyspnoea

The studies showed little to no difference between maintenance and usual care groups concerning the mMRC, and high heterogeneity was detected (MD −1.2 pts; CI −2.5 to 0.1; I^2^ = 99%; *p* < 0.0001)^34,36,43^ On sensitivity analysis, we observed that the one study^43^, although with the more pronounced potential benefit, is the one most contributing to heterogeneity. As there are only three studies with data, no subgroup analyses were performed.

### Secondary outcome - Symptoms control

The studies showed little to no difference between maintenance and usual care groups concerning symptom control, using the Chronic Respiratory Disease Questionnaire^33,37,39–41^.

### Secondary outcome - Exacerbations

Exacerbations were monitored in five studies^32,33,35,40,43^. The mean number of exacerbations in the maintenance programmes was lower than in the control group but without significant estimates (Intervention: 17.8 exacerbations; Control: 18.8 exacerbations).

## Discussion

This review has synthesised the available evidence of the long‐term effects and the structure of maintenance PR programmes in COPD patients compared to usual care. Our results confirm that such programmes significantly increased functional capacity. This improvement was more pronounced in supervised PR programmes, in those with longer duration (over 12 months), in a home-based setting, and after initial programmes with more than eight weeks.

Our results are aligned with recent meta-analyses^11,48^, showing that maintenance PR programmes confer long-term efficacy on functional capacity in COPD patients compared to usual care.

We found that different types of follow-up had different effects on patients. High certainty evidence suggests that more extended programmes (>12 months) seem superior to shorter ones compared to usual care. This comes with no surprise, as exercise training is known to play a central role in PR, and maintaining that training is one of the most critical factors^2,4^. However, continuing more extended PR programmes is often difficult due to a lack of human resources and, in home-based programmes, the need for healthcare professionals to visit the patient’s home. Additionally, the costs of delivering such maintenance programmes have not been well documented across different models. The impact of maintenance programmes on other healthcare costs (e.g. hospitalisation, primary care visits) is unknown and should be the topic of further studies.

We could also demonstrate that home-based programmes are associated with better maintenance of patients’ functional capacity compared to usual care. This is not unexpected, as a Cochrane Review^48^ comparing PR or maintenance rehabilitation in COPD patients showed that telerehabilitation improved exercise capacity in maintenance programmes. Additionally, rehabilitation at home has been shown to improve long-term HRQoL^11^.

While home-based settings are convenient for the patient, they may lack the equipment or floor space. In addition, it may be challenging to determine the progress within a therapy session. Nevertheless, home-based maintenance PR programmes provide an adequate setting for promoting pedometer-based physical activity, diaries and guidebooks^49^, which have demonstrated, using positive feedback, increasing the number of steps-per-day performed by patients^50^, which can be ways to achieve better physical activity levels in COPD patients following PR^51^. Furthermore, such feedback mechanisms are essential to motivation, which plays a central role in behaviour change.

The cornerstone of maintenance programme success might be engaging COPD patients in therapeutic adherence, considering they are time-consuming and need a stable commitment to the plan^49^. For that reason, supervision by healthcare professionals seems important to achieve better results, either in centre-based or home-based interventions.

Maintenance PR programmes included in the meta‐analysis differed in several aspects, including clinical setting, duration and composition. However, our work demonstrates the superiority of supervised training during the programme compared to usual care, rather than other modalities, having supervised programmes have a mean increment of 62 meters in MD between groups compared to unsupervised programmes (Supplementary Information 4), following other studies^8,11^. In addition, home-based PR programmes seem to have better outcomes with structured schedules, including health professional visits and regular phone calls between them^38,39,41,43^.

Supervised PR maintenance programmes are effective in the early stages to better tailor exercise training to the patient and thereby increase programme compliance^2,3,10^, and can progressively be replaced by non-supervised sessions, maintaining a good impact on functional capacity and decreasing health system burdens. This “stepwise approach” in time, with tighter supervision in the early stages of the programme, gradual increase of more spaced visits and telephone calls, and unsupervised strategies in later stages, might lead to long-lasting changes in behaviour and support patients’ autonomy and responsibility for their health status while optimizing resources. This is an important finding regarding programme feasibility and resources needed to implement it^35–38,41,44^.

Our review could not find evidence of the ideal core components of maintenance PR programmes, such as the type of intervention and its specific components or the appropriate duration/format/setting according to each specific profile of COPD patient. However, the effect of different components of rehabilitation in COPD, such as contributions of educational activities and psychological support to exercise training, would be relevant to physicians and allied healthcare professionals who prescribe rehabilitation and policymakers who allocate the resources.

As patients with COPD are concerned mainly with treating symptoms^1^, HRQoL should be considered an additional critical outcome in PR. The present meta‐analysis reconfirms the previous findings^52^ that PR effectively relieves dyspnoea and fatigue and improves patients’ control over the disease.

Of particular relevance, our findings showed that more severe patients do not seem to improve symptom control with shorter (<6 months) maintenance programmes. These results are aligned with Salman et al. ^53^, which demonstrated that severe COPD rehabilitation groups did significantly better than control groups only when the rehabilitation programmes were six months or longer. Severe COPD patients have a fluctuating course of the disease, with frequent exacerbations and a vicious cycle of increasing dyspnoea, which leads to decreased exercise tolerance, depression, and social isolation, all predictors of poor HRQoL and high risk of mortality^54^. Furthermore, from a rehabilitation point of view, such patients present skeletal muscle wasting, which further contributes to muscle fatigue during exercise. This can lead patients to stop exercising even before reaching their aerobic capacity^55^. However, patients with severe COPD are the subgroup that shows the most improvements after PR^56^, which might mean that this subgroup of patients will benefit the most from long-term interventions. Therefore, despite the lack of suggestions for long-term rehabilitation schemes (>6 months) in severe COPD patients in current guidelines^2^, this subgroup should be actively offered home-based, supervised, long-term programmes, according to the best available evidence.

Owing to the paucity of data in included studies, we could not establish the ideal structure of PR, mainly regarding its specific components or even specific features/duration/setting to each profile of COPD patient.

This systematic review has limitations. The intervention was heterogeneous among included studies in terms of duration, type, setting or supervision. Most studies had an unclear and high risk of bias in their global quality rating, with some evidence of significant heterogeneity across studies, limiting the strength of evidence and the confidence of our findings. Given the objective of this systematic review, we understand that by excluding programmes that have not delivered a comprehensive PR programme as defined^2,4^ before the maintenance PR programme, we could have lost some relevant studies that could have reached different results. The same concerns other outcome measures such as the endurance shuttle walking test (ESWT) and the incremental shuttle walking test (ISWT). However, the ESWT and the ISWT have been used in fewer studies and show little to no difference between maintenance and usual care^11^. Our pooled estimate using the 6mWT was the same as a previous review^8^.

However, this review has some strengths. It has been conducted with a robust methodology, following the Cochrane Handbook recommendations^13^, and our search was comprehensive. We also performed a GRADE-based assessment of the quality of evidence and found limited reasons to suspect publication bias according to the funnel plot (Supplementary Information 4).

Our results highlight the importance of PR maintenance programmes on COPD treatment, mainly when home-based, supervised and long-term, delivered after an initial PR intervention. COPD patients submitted to maintenance PR programmes seem to benefit from significant improvements in functional capacity and other clinical outcomes, ultimately reducing the risk for future exacerbations and death. Functional capacity seems to be improved after 12 months or more if both education and physical exercise are present in the patient’s daily routine. More long-term studies are needed to evaluate the potential benefit of maintenance PR programmes above the 12-month horizon, mainly in relevant clinical outcomes, such as clinical control and exacerbation risk. Finally, future guidelines and stakeholders should reinforce the importance and investment in maintenance PR programmes, as they play a crucial role in COPD treatment.

We recommend that supervised, home-based maintenance PR programmes lasting 12 months be routinely offered as an add-on to remaining treatment strategies since the programmes with this structure increase functional capacity in COPD patients. In addition, severe COPD patients should be offered more extended programmes (>12 months).

Regarding dyspnoea and exacerbations, we found a non-significant trend for improvement after maintenance PR programmes. Severe COPD patients showed smaller improvements in programmes for up to a year. Overall, the strength of the underlying evidence was moderate.

## Supplementary information

REPORTING SUMMARY

Supplementary material

## Data Availability

Raw data were available from the cited articles. In addition, derived data supporting the findings of this study are available from the corresponding author (LS) on request.
