# Supplementary material for: A meta-analysis on the structure of pulmonary rehabilitation maintenance programmes on COPD patients’ functional capacity
Source: NPJ Prim Care Respir Med. 2022 Oct 3;32:38. doi: 10.1038/s41533-022-00302-x (PMC9530215; doi:10.1038/s41533-022-00302-x)
Supplement: Supplementary file 2 — Supplementary material [file 41533_2022_302_MOESM2_ESM.pdf]

**Supplementary information 1 –** Search strategy used

**Web of Science**

(COPD OR EPOC OR DPOC OR "Chronic Obstructive Pulmonary Disease") AND ("Pulmonary rehabilitation" OR "respiratory rehabilitation") AND (Trial OR "RCT" OR "Randomized Controlled trial" OR "Experimental")

**Scopus (Embase e MEDLINE)**

(COPD OR EPOC OR DPOC OR "Chronic Obstructive Pulmonary Disease") AND ("Pulmonary rehabilitation" OR "respiratory rehabilitation") AND (Trial OR "RCT" OR "Randomized Controlled trial" OR "Experimental")

**The Cumulative Index to Nursing and Allied Health Literature [CINAHL] Complete**

(COPD OR EPOC OR DPOC OR "Chronic Obstructive Pulmonary Disease") AND ("Pulmonary rehabilitation" OR "respiratory rehabilitation") AND (Trial OR "RCT" OR "Randomized Controlled trial" OR "Experimental")

**Cochrane Library**

(COPD OR EPOC OR DPOC OR "Chronic Obstructive Pulmonary Disease") AND ("Pulmonary rehabilitation" OR "respiratory rehabilitation") AND (Trial OR "RCT" OR "Randomized Controlled trial" OR "Experimental")

**Supplementary information 2** – Complete data of selected studies

| Author, year | Study type | Sample size | Age (mean±sd) | Gender (%M, %F) | Pre PR Program duration (wks) | Intervention | | FOLLOW_UP (months) | Supervision (SV supervised) (USV unsupervised) (AS – Alt. supervision) | SETTING (Home-based (HB), centre-based(CB), tele(Tele)) | Disease Severity (FEV1 % pred) | 6mWT | | mMRC | | QoL *EQ5D **SGRQ ***SF36 | | CRDQ | |
|---|---|---|---|---|---|---|---|---|---|---|---|---|---|---|---|---|---|---|---|
| | | | | | | Usual Care | Intervention group | | | | | Mean diff (sd) Control | Mean diff (sd) Intervention | Mean diff (sd) Control | Mean diff (sd) Intervention | Mean diff (sd) Control | Mean diff (sd) Intervention | Mean diff (sd) Control | Mean diff (sd) Intervention |
| Bauldoff, G. 2002 | RCT | I: 12 C:12 | 68.1 (±8) | 17/83 | Not reported | Exercise training and walks without music | Use of music during exercise training and walks | 2 | SV | HB | NR | -51.5 (81.73) | 135.8 (97.15) | NA | NA | NA | NA | NA | NA |
| Brooks, D. 2002 | RCT | I: 37 C:48 | I:68(±1.1) C:68(±1.1) | I:60/40 C:59/41 | inpatients: 6; outpatients: 8 | Physical therapist visit every 3 months to encourage their exercise programme. | Prescription of home programme and phone call between visits asking standardised questions regarding adherence. Monthly group sessions to reinforce and encourage their exercise programme. | 12 | USV | Alt HB and CB | Moderate to Severe | 8 (4) | -14 (15.5) | NA | NA | NA | NA | NA | NA |
| Butler, S.J. 2020 | RCT | I:49 C:48 | I:68(±9) C:69(±9) | I:57/43 C:46/54 | Not reported | Standard home exercise instructions from their physical therapist + routine medical. | The same standard care as the control group plus a community-based maintenance exercise program. Participants were encouraged to exercise at the centre. Group classes were led by a certified fitness instructor with training in COPD supervision. | 12 | AS | CB | Moderate to Severe | -8.6 (110.7) | -4.9 (106.19) | NA | NA | NA | NA | 0(1) | 0.1 (1.1) |
| Cockram, J. 2006 | quasi-experimental | 230 | 68.9(±7.4) | 71.4/28.6 | 8 | NA | Endurance component (e.g. walking), upper limb and lower limb exercises, and activities for balance, flexibility and strength. All participants were encouraged to continue with their home programme on three or four additional days per week. | 12 | SV | CB | NR | NA | 41.1 (15.7-66.5) | NA | NA | NA | NA | NA | NA |

**Supplementary information 2** – Complete data of selected studies

| | | | | | | | | | | | | | | | | | | | |
|---|---|---|---|---|---|---|---|---|---|---|---|---|---|---|---|---|---|---|---|
| Cruz, J. 2016 | RCT | I: 16<br>C:16 | I: 68.8 (±8.2)<br>C:64.1 (±8.2) | I:81.2/18.8<br>C:87.5/12.5 | 12 | The same programme as the intervention group | Participants were given a piezoelectric pedometer and a log diary to record daily steps, and a written contract agreement. Participants also received a calendar to register their short-term step-count goals and daily steps, which were self-monitored with the pedometer. Short-term goals were defined on a weekly basis and consisted of the previous short-term goal plus approximately 800 additional steps or the previous goal. The final aim was to achieve the long-term goal. In each session, the physiotherapist provided individual feedback. Patients continued registering their steps in the calendar and received the physiotherapist's support on a weekly (in the first month) and fortnightly (in the second and third months) basis by telephone calls. | 3 | AS | Alt HB and CB | Mild to severe | 53.5 (56.09) | 54.1 (56.97) | NA | NA | **-8 (11.4) | **-7.5 (14.8) | NA | NA |
| du Moulin, M. 2008 | RCT | I:10<br>C:10 | I:67 (63-72)<br>C:72 (69-77) | 70/30 | 3 | No specific instructions were given regarding physical activities during the period following rehabilitation. | Patients in the maintenance group received an individualized training plan, based on their last 6MWT, measured following completion of the 3-week outpatient rehabilitation program. Patients were instructed to quickly walk a distance equivalent to 125% of their last 6MWT three times a day with each training walk not exceeding 15 min.<br>Patients were given a pedometer so that the training could be better incorporated into daily activities in a homebased setting, and qith a training diary. Patients were also contacted by telephone every 4 weeks for motivation.. | 6 | USV | HB | NR | -11.5 (75.72) | 33.9 (76.04) | NA | NA | NA | NA | -0.3 (0.8) | 0.4 (0.7) |
| Galdiz, J B., 2021 | RCT | I:48<br>C:46 | I:63 (±6.6)<br>C:62.3 (±8.2) | I:65.2/34.8<br>C:68.8/31.2 | 8 | Advised to excercise regularly and provided with general education material, following current clinical practice at participating sites. | Three trainning sessions a week which included 30 min of weight lifting and 30 min of leg cycle ergometry along with four educational sessions wich included chest physiotherapy training. | 12 | USV | Tele | moderate to severe | -25.5 (97.24) | -3.3 (104.2) | NA | NA | NA | NA | 0(1.4) | -0.17 (1.4) |

## **Supplementary information 2** – Complete data of selected studies

| | | | | | | | | | | | | | | | | | | | |
|---|---|---|---|---|---|---|---|---|---|---|---|---|---|---|---|---|---|---|---|
| Guell, M-R. 2017 | RCT | I:68 C:70 | I:64(±8 ) C:64(± 9) | I:91/ 9 C:87 /13 | 8 | Maintenance of the exercise at home, without any supervision. Advised to walk at least 1 hour or buy a cycle ergometer. | Maintenance at home with a similar program to that completed in the hospital including 15 minutes of chest physiotherapy, 30 minutes of arm training, and 30 minutes of leg training (at least 3 times/weekly).<br>Use of cycle ergometers and weights. Calls to patients every 15 days using a standardized protocol. If the exercise was well tolerated, the home training was increased to one guided by the patient's dyspnea, oxygen saturation as measured by pulse, and heart rate. | 36 | AS | HB | moder ate to severe | -33 (88) | -4 (111) | NA | NA | ***-3 (18) | ***-4 (2) | -0.27 (1.4) | -0.5 (1.4) |
| Jiménez-Reguera 2020 | RCT | I: 20 C: 24 | I:68.1 (±6.6) C:68.1 (±7.0) | I:40. 9/57. 1 C:59. 1/42. 9 | 8 | Usual follow up | The HappyAir app comprises two main parts: an educational program providing patients useful information and advice about their illness and data collection related to physical activity and disease, and daily reminders.<br>After patients finished pulmonary rehabilitation, we offered an education session that consisted of 3 to 4 hours of practical class demonstration plus an online support aid. The therapeutic educators supervised the evolution of the HappyAir group.<br>The HappyAir integrated plan was designed as a model of a therapeutic program based on communication that introduced the figure of the therapeutic educator (physiotherapist or respiratory coach) in order to design interventions focused on the patients and their needs, with minimal intervention and presence, making the patients responsible for their self-care and management of their illness. | 10 | US V | Tele | moder ate to severe | 28.8 (70.7) | 42.2 (84.26) | NA | NA | **-2.7 (44.9) | **-3.7 (11.6) | NA | NA |
| Li, Y. 2018 | RCT | I:65 C:69 | I:65 (±8.7) C:66 (±9.3) | I:87/ 13 C:80 /20 | 8 | Health education, smoking cessation, oxygen therapy when patients suffered respiratory failure, as well as dietary | Performed at home. The frequency of aerobic and upper resistance training was once per week, and respiratory training was 3 times per week. Additionally, patients adjusted speed or exercise intensity according to maximum heart rate | 12 | SV | HB | mild to severe | -19 (44.5) | 53 (64.5) | 1.2 (0.82) | -1 (0.89) | NA | NA | NA | NA |

| | | | | | | | | | | | | | | | | | | | |
|---|---|---|---|---|---|---|---|---|---|---|---|---|---|---|---|---|---|---|---|
| | | | | | | and nutrition consultation along with necessary pharmacological therapy without systemic corticosteroids. | and symptoms when they were ready for aerobic training.<br>Were defined 3 evolutionary steps to supervise patients' performance and adherence: Step 1: Home-visit once every 2 weeks to conduct rehabilitation exercise strategy and provide health education for 2 months. Step 2: Home-visit every 4 weeks and phone contact once a week for 4 months. Step 3: Phone contact once a week for 6 months. | | | | | | | | | | | | |
| Moullec, G. 2010 | quasi-experimental | I:14 C:26 | I:62.9 (±7.4) C:59.7 (±9.6) | I:71/29 C:81/19 | 4 | Health education, smoking cessation, oxygen therapy when patients suffered respiratory failure, as well as dietary and nutrition consultation along with necessary pharmacological therapy without systemic corticosteroids. | Individualized exercise training (3.5 hours/week; 72 sessions) supervised by a teacher of adapted physical activities including breathing exercises, interval training, strength training, upper limb training and endurance training with nature walking at the ventilatory threshold; health education provided alternatively by all professionals of the health care network (2 hours/month; 12 sessions) in a municipal conference room; and psychosocial support with discussion group (1 hour/month; 12 sessions) supervised by a psychologist in the same room. | 12 | SV | CB | moderate to severe | -37.1 (82) | 39.3 (84.9) | NA | NA | NA | NA | NA | NA |
| Ries, a.L 2003 | RCT | I:64 C:74 | 67.1 (±8.2) | 54.3/45.7 | 8 | Home-based exercise programme at discharge. Usual primary care follow-up for one year. | 1-weekly telephone calls;<br>2-monthly supervised reinforcement sessions, about compliance with the individual home care plan and health problems, and similar to the inicial rehabilitation program sessions. | 12 | AS | Alt HB and CB | mild to severe | -43 (117) | -17.9 (107.68) | NA | NA | NA | NA | NA | NA |
| Román, M. 2013 | RCT | I:26 C:22 | I:64.9 (62.1-67.7) C:64.1 (59.9-68.2) | I:80.8/19.2 C:81.8/18.2 | 12 | Routine care with their physician and nurse. | Weekly-session maintenance program, including both respiratory physiotherapy and low intensity peripheral muscle training, until the end of the program at 12 months. | 12 | SV | CB | Mild to severe | -20.2 (70.5 | -19.7 (45.7) | NA | NA | NA | NA | 0 (1.8) | 0.44 (1.8) |
| Spencer, L.M. 2010 | RCT | I:24 C:24 | I:41.6/58.3 C:50/50 | I:41.6/58.3 C:50/50 | 8 | Unsupervised home exercise 5 days per week, plus home exercise booklet and diary. | Supervised, outpatient-based exercise 1 day per week plus unsupervised home exercise on four other days.<br>On the day subjects attended the gymnasium, they performed the same exercise regimen as during | 12 | AS | Alt HB and CB | Moderate | 10 (78.6) | 62 (91.43) | NA | NA | **-3 (16.7) | **3 (14.1) | NA | NA |

| | | | | | | | | | | | | | | | | | | | |
|---|---|---|---|---|---|---|---|---|---|---|---|---|---|---|---|---|---|---|---|
| | | | | | | | the pulmonary rehabilitation programme (20 min walking, 20 min cycling, 10 min arm cycling, and upper and lower limb strength training exercises using weight equipment and free weights). Unsupervised home exercise consisted of 30 min of walking plus 30 min of upper and lower limb strengthening exercises using free weights and body weight. All subjects had an illustrated home exercise booklet to guide them, plus a diary for recording sessions completed. | | | | | | | | | | | | |
| Souza, Y. 2018 | RCT | I:25 C:25 | I:40/60 C:56/44 | I:40/60 C:56/44 | 12 | Oral informations about performing the exercises learned during the program served as the control group | Patients received the manual for activities and were instructed to use it daily (figures with brief descriptions). The manual has the same program as the outpatient rehabilitation, and the 20 repetitions of each exercise were used to facilitate the comprehension and memorizations of the exercise program. The subjects were instructed to selfmeasure by using the Borg scale after the exercises. When the subjects experienced strong breathlessness, they were instructed to use the breath control technique, to use inhalation medication or, in severe cases, to call their physician. | 6 | USV | HB | mild to severe | -6 (82.16) | -11 (108.5) | -1 (1) | -2 (1) | NA | NA | NA | NA |
| van Wetering, CR 2010 | RCT | I:102 C:97 | I:65.9 (±8.8) C:67.2 (±8.9) | 71/29 | 12 | The usual care group received pharmacotherapy according to accepted guidelines, a short smoking cessation advice by their chest physician and, if they were nutritionally depleted, a recommendation by their respiratory | Patients were instructed to perform the same exercises twice a day during 30 min in their home environment in addition to walking and cycling outside, plus individualised education programme that was structured using a patient education book and smoking cessation counselling. Monthly encouragments to programme adherence and to home training. After exacerbations, an six extra training sessions in 3 weeks at the physiotherapy practice were perfomed, plus dietician visits four | 20 | AS | HB | moderate to severe | -15.3 (3.9) | -1.4 (3.9) | 0.1 (0.1) | -0.3 (0.1) | **0.3 (1) | **-3.9 (1.1) | NA | NA |

| | | | | | | | | | | | | | | | | | | | |
|---|---|---|---|---|---|---|---|---|---|---|---|---|---|---|---|---|---|---|---|
| | | | | | | physician to eat more.. | times in the maintenance phase and ocasional visits to the respiratory nurse. | | | | | | | | | | | | |
| Zanaboni 2017 | quasi - expe rime ntal | 10 | 55.2 (±6.1) | 50/5 0 | 4 | NA | Telerehabilitation consisting in home exercise, telemonitoring and self-management, supervised by a physiotherapist. Equipment for home exercise consisted of a treadmill installed in the patients' homes (supported speed up to 16 km/h and incline up to 10%). Videoconferencing was initiated from a rehabilitation centre and performed through the application running on the participant's tablet. Participants used the web-based platform to access an individual training programme, to fill in a daily diary and a training diary, and to access historical data. The information was also accessible to the physiotherapist and used for discussion with the patients. | 24 | ASV | HB | mild to severe | NA | -56 (107) | NA | NA | NA | *-0.07 (0.21) | NA | NA |

**Supplementary information 3 - Quality Assessment and Risk of Bias Table:**

| | Initial Classification (evidence; risk) | Weaknesses | | Strengths | Risk of Bias in GRADE | | | | | | | Final Classification (evidence; risk) |
|---|---|---|---|---|---|---|---|---|---|---|---|---|
| | | Limitations | Risk of Bias | | 1 | 2 | 3 | 4 | 5 | 6 | 7 | |
| **Bauldofd, G. 2002** | Moderate evidence; High risk | Small sample size<br>Short follow-up<br>No adequate blinding | high | -- | ? | ? | - | ? | ? | - | + | Low evidence; High risk |
| **Brooks, D, 2002** | High evidence; high risk | No exact data<br>Only per-protocol analysis<br>Small sample size | uncertain | Outcome measure blinding | + | ? | - | + | ? | + | + | High evidence; uncertain risk |
| **Butler, S.J. 2020** | High evidence; low risk | Small sample size<br>Lack of participants blinding. | low | Outcome measure blinding<br>Adequate randomization and allocation | + | + | - | + | + | + | + | High evidence; low risk |
| **Cockram, J. 2006** | Low evidence; high risk | Quasi-experimental<br>Small sample size<br>No data from the post-PR programme/maintenance baseline.<br>No adequate blinding | high | -- | - | - | - | - | - | - | + | Low evidence; High risk |
| **Cruz, J. 2016** | High evidence; high risk | Lack of blinding for the secondary outcomes<br>Small sample size<br>Short follow-up | high | Intention to treat analysis<br>Good study design<br>Primary outcome measure blinding<br>Adequate randomization and allocation | + | + | - | - | + | + | + | Moderate evidence; Uncertain risk |
| **du Moulin, M. 2008** | High evidence; low risk | Small sample size<br>Short follow-up<br>Outcome measurement at baseline is pre-PR initial programme | Low | Outcome measure blinding<br>Adequate randomization and allocation | + | + | - | + | + | + | + | High evidence; low risk |
| **Galdiz, JB. 2021** | High evidence, uncertain risk | No reference to outcome assessment blinding<br>Only per-protocol analysis<br>Small sample size | uncertain | Adequate randomization and allocation | + | + | - | ? | + | + | + | High Evidence; High Risk |
| **Guell, M-R., 2017** | High evidence; low risk | Uncertain allocation and participants blinding | uncertain | Good sample size<br>Outcome measure blinding<br>Long follow-up | + | ? | - | + | + | + | + | High evidence; Uncertain risk |
| **Jiménez-Reguera, B. 2020** | High evidence; low risk | Small sample size<br>Outcome measurement at baseline is pre-PR initial programme | Low | Outcome measure blinding<br>Adequate randomization and allocation | + | + | - | + | + | + | + | High evidence; low risk |
| **Li, Y. 2018** | High evidence; low risk | No adequate blinding | high | Good sample size | ? | ? | - | ? | + | + | + | High evidence; Uncertain risk |

| | | | | | | | | | | | | |
|---|---|---|---|---|---|---|---|---|---|---|---|---|
| **Moullec, G. 2010** | Moderate evidence; High risk | Small sample size<br>No randomization | high | Outcome measure blinding | - | - | - | + | + | + | - | Low evidence; High risk |
| **Ries, 2003** | High evidence; uncertain risk | Incomplete outcome measure blinding<br>No adequate allocation and participants blinding | high | Good sample size | - | - | - | - | + | + | + | Moderate evidence; High risk |
| **Román, M, 2013** | High evidence; low risk | Small sample size<br>Only per-protocol analysis<br>No adequate blinding | uncertain | Adequate randomization and allocation | + | + | - | ? | + | + | + | High evidence; uncertain risk |
| **Souza, Y. 2018** | Moderate evidence; uncertain risk | Small sample size<br>Short follow-up<br>No adequate blinding | uncertain | Intention to treat analysis<br>Adequate randomization and allocation | + | + | - | ? | + | + | + | Moderate evidence; Uncertain risk |
| **Spencer, L.M. 2010** | High evidence; low risk | Small sample size<br>No adequate blinding | uncertain | Adequate randomization and allocation | + | + | - | ? | + | + | + | High evidence; uncertain risk |
| **van Wetering, CR, 2010** | High evidence; low risk | Only per-protocol analysis | Low | Good sample size<br>Long follow-up<br>Adequate randomization and allocation | + | + | - | + | + | + | + | High evidence; low risk |
| **Zanaboni, 2017** | Low evidence; high risk | Quasi Experimental<br>Small sample size<br>No adequate blinding | high | Long follow-up | - | - | - | - | + | + | + | Low evidence; High risk |

1 - Random Sequence Generation
2 - Allocation Concealment
3 - Blinding of Participants and Personnel
4 - Blinding of Outcome Assessment
5 - Incomplete Outcome Data
6 - Selective Reporting
7 - Other source of Bias
PR - pulmonary rehabilitation

## Risk of Bias assessment summary in included studies according to GRADE system

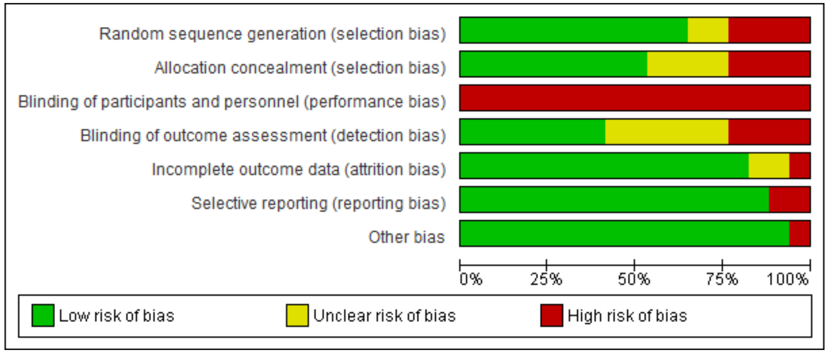

Detailed Risk of Bias assessment in included studies according to GRADE system

| | Random sequence generation (selection bias) | Allocation concealment (selection bias) | Blinding of participants and personnel (performance bias) | Blinding of outcome assessment (detection bias) | Incomplete outcome data (attrition bias) | Selective reporting (reporting bias) | Other bias |
|---|---|---|---|---|---|---|---|
| Bauldoff, G. 2002 | ? | ? | − | ? | ? | − | + |
| Brooks, D. 2002 | + | ? | − | + | ? | + | + |
| Butler, S. 2020 | + | + | − | + | + | + | + |
| Cockram, J. 2006 | − | − | − | − | − | − | + |
| Cruz, J. 2016 | + | + | − | − | + | + | + |
| du Moulin, M. 2008 | + | + | − | + | + | + | + |
| Galdiz, J.B. 2021 | + | + | − | ? | + | + | + |
| Guell, M-R., 2017 | + | ? | − | + | + | + | + |
| Jiménez-Reguera, B. 2020 | + | + | − | + | + | + | + |
| Li, Yi. 2018 | ? | ? | − | ? | + | + | + |
| Moullec, G. 2010 | − | − | − | + | + | + | − |
| Ries, A. 2003 | − | − | − | − | + | + | + |
| Román, M. 2013 | + | + | − | ? | + | + | + |
| Souza, Y. 2018 | + | + | − | ? | + | + | + |
| Spencer, L.M. 2010 | + | + | − | ? | + | + | + |
| Van Wetering, C.R. 2010 | + | + | − | + | + | + | + |
| Zanaboni, P. 2017 | − | − | − | − | + | + | + |

**Supplementary Information 4 –** Complete data of Meta-analysis and Meta-regression

## Summary of findings

| OUTCOME | Functional Capacity (6mWT) ES(CI) | QoL (SGRQ) ES(CI) | Symptoms (CRDQ) ES(CI) | Dyspnea (mMRC) ES(CI) |
|---|---|---|---|---|
| **Total – All studies** | **27.08(10.39;43.77)*** ** | **-0.75(-6.22;4.73)*** ** | **0.10 (-0.22;0.42)** ** | **-1.20 (-2.50;-0.37)*** ** |
| **Subgroup analysis** | | | | # |
| Programme duration | | | | |
| ≤6 months | 53.41 (-23.28;130.10)*** | 0.5 (-8.65;9.65)† | 0.70 (0.04;1.36)† | |
| 12 months | 25.82 (-6.99;58.62)*** | | 0.00 (-0.33;0.34)* | |
| > 12 months | **13.92 (12.83;15.00)*** | -0.53 (-8.14;7.08)*** | -0.00(-0.62; 0.62)** | |
| Initial PR Programme duration | | | | |
| 3-6 weeks | 28.91 (-41.80;99.63)*** | †† | 0.70 (0.04;1.36) † | |
| 8 weeks | 38.74 (15.90;61.57)*** | 4.74 (-3.18;12.66)* | -0.21 (-0.57;0.15)* | |
| 12 weeks | **13.86 (12.78;14.95)*** | **-4.17 (-4.92; -3.41)*** | 0.44 (-0.44;1.32) † | |
| COPD severity | | | | |
| Mild to severe | **13.89 (12.81;14.97)*** | -0.75(-6.22;4.73)*** | 0.44 (-0.44;1.32) † | |
| Moderate to severe | 24.73 (-15.89;65.34)*** | †† | -0.08 (-0.35;0.20)* | |
| Professional supervision | | | | |
| Supervised | **76.66 (19.22;134.09)*** ** | 0.50 (-8.65;9.65)† | 0.44 (-0.44;1.32) † | |
| Unsupervised | 3.52 (-23.88;30.93)*** | -1.00 (-19.67;17.67)† | 0.25 (-0.60;1.10)*** | |
| Alternate supervision | **13.92 (12.84;15.00)*** | -0.07(-9.89;9.74)*** | -0.05 (-0.37;0.27)** | |
| Setting | | | | |
| Home-based (HB) | **50.48 (13.59;87.37)*** ** | **-4.20 (-4.49;-3.91)†** | 0.20 (-0.70;1.11)*** | |
| Community-based (CB) | 22.01 (-19.61; 63.63)*** | †† | 0.16 (-0.21;0.54)* | |
| Alternate HB/CB | 9.62 (-25.26;44.50)** | 3.38 (-2.95;9.70)** | †† | |
| Telerehabilitation | 18.38 (-12.26;49.02)** | -1.0 (-19.67;17.67) † | -0.17 (-0.74;0.40)† | |
| Methodological quality | | | | |
| High risk of bias | 66.53 (0.85;132.22)*** | **3.38 (-2.95;9.7)*** | †† | |
| Uncertain risk of bias | 21.14 (-16.42;58.70)*** | | -0.11 (-0.45;0.22)* | |
| Low risk of bias | **13.90 (12.82;14.98)*** | **-4.20 (-4.49; -3.91)*** | 0.34 (-0.23;0.92)** | |

** $I^2$=0%; **$I^2$≤50%; ***$I^2$>50%;*

† One study only; ††No Studies available; #No subgroup analyses were performed to the outcome "dyspnea" considering that it includes only 3 studies.

*ES – Effect size QoL – Quality of Life;*

## OUTCOME: Six minute walking test (6MWT)

### All studies

| Study or Subgroup | MPR Mean | MPR SD | MPR Total | Control Mean | Control SD | Control Total | Weight | Mean Difference IV, Random, 95% CI |
|---|---|---|---|---|---|---|---|---|
| Bauldoff, G. 2002 | 135.8 | 97.15 | 12 | -51.5 | 81.7 | 12 | 3.6% | 187.30 [115.48, 259.12] |
| Brooks, D. 2002 | -14 | 15.5 | 37 | 8 | 14 | 48 | 10.3% | -22.00 [-28.37, -15.63] |
| Butler, S. 2020 | -4.9 | 106.2 | 49 | -8.6 | 110.7 | 48 | 6.1% | 3.70 [-39.48, 46.88] |
| Cruz, J. 2016 | 54.1 | 56.97 | 16 | 53.5 | 56.09 | 16 | 6.6% | 0.60 [-38.57, 39.77] |
| du Moulin, M. 2008 | 33.9 | 76.04 | 10 | -11.5 | 75.72 | 10 | 3.9% | 45.40 [-21.11, 111.91] |
| Galdiz, J.B. 2021 | -3.3 | 104.2 | 48 | -25.5 | 97.24 | 46 | 6.4% | 22.20 [-18.53, 62.93] |
| Guell, M-R., 2017 | -4 | 111 | 68 | -33 | 88 | 70 | 7.3% | 29.00 [-4.48, 62.48] |
| Jiménez-Reguera, B. 2020 | 42.2 | 84.26 | 20 | 28.8 | 70.7 | 24 | 5.8% | 13.40 [-33.12, 59.92] |
| Li, Yi. 2018 | 53 | 64.5 | 65 | -19 | 44.5 | 69 | 9.2% | 72.00 [53.13, 90.87] |
| Moullec, G. 2010 | 39.3 | 84.9 | 14 | -37.1 | 82 | 26 | 4.9% | 76.40 [21.89, 130.91] |
| Ries, A. 2003 | -17.9 | 107.68 | 64 | -43 | 117 | 74 | 6.8% | 25.10 [-12.40, 62.60] |
| Román, M. 2013 | -19.7 | 45.7 | 32 | -20.2 | 70.5 | 33 | 8.0% | 0.50 [-28.30, 29.30] |
| Souza, Y. 2018 | -11 | 108.5 | 24 | -6 | 82.16 | 24 | 4.9% | -5.00 [-59.45, 49.45] |
| Spencer, L.M. 2010 | 62 | 91.43 | 25 | 10 | 78.6 | 25 | 5.7% | 52.00 [4.74, 99.26] |
| Van Wetering, C.R. 2010 | -1.4 | 3.9 | 102 | -15.3 | 3.9 | 97 | 10.4% | 13.90 [12.82, 14.98] |
| **Total (95% CI)** | | | **586** | | | **622** | **100.0%** | **27.08 [10.39, 43.77]** |

Heterogeneity: Tau² = 695.56; Chi² = 190.46, df = 14 (P < 0.00001); I² = 93%
Test for overall effect: Z = 3.18 (P = 0.001)

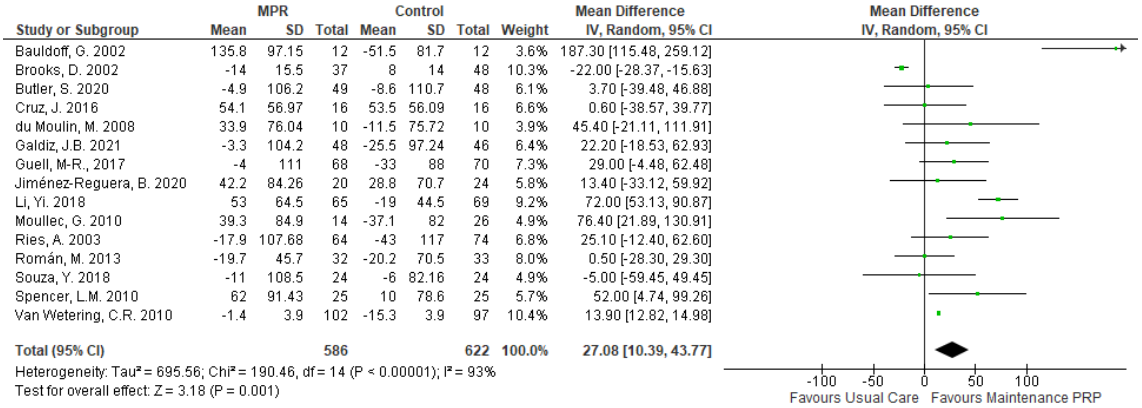


Sensitive analysis, excluding Brooks D 2002 effect:

| Study or Subgroup | MPR Mean | MPR SD | MPR Total | Control Mean | Control SD | Control Total | Weight | Mean Difference IV, Random, 95% CI |
|---|---|---|---|---|---|---|---|---|
| Bauldoff, G. 2002 | 135.8 | 97.15 | 12 | -51.5 | 81.7 | 12 | 4.1% | 187.30 [115.48, 259.12] |
| Brooks, D. 2002 | -14 | 15.5 | 37 | 8 | 14 | 48 | 0.0% | -22.00 [-28.37, -15.63] |
| Butler, S. 2020 | -4.9 | 106.2 | 49 | -8.6 | 110.7 | 48 | 6.9% | 3.70 [-39.48, 46.88] |
| Cruz, J. 2016 | 54.1 | 56.97 | 16 | 53.5 | 56.09 | 16 | 7.4% | 0.60 [-38.57, 39.77] |
| du Moulin, M. 2008 | 33.9 | 76.04 | 10 | -11.5 | 75.72 | 10 | 4.5% | 45.40 [-21.11, 111.91] |
| Galdiz, J.B. 2021 | -3.3 | 104.2 | 48 | -25.5 | 97.24 | 46 | 7.2% | 22.20 [-18.53, 62.93] |
| Guell, M-R., 2017 | -4 | 111 | 68 | -33 | 88 | 70 | 8.1% | 29.00 [-4.48, 62.48] |
| Jiménez-Reguera, B. 2020 | 42.2 | 84.26 | 20 | 28.8 | 70.7 | 24 | 6.5% | 13.40 [-33.12, 59.92] |
| Li, Yi. 2018 | 53 | 64.5 | 65 | -19 | 44.5 | 69 | 10.0% | 72.00 [53.13, 90.87] |
| Moullec, G. 2010 | 39.3 | 84.9 | 14 | -37.1 | 82 | 26 | 5.6% | 76.40 [21.89, 130.91] |
| Ries, A. 2003 | -17.9 | 107.68 | 64 | -43 | 117 | 74 | 7.6% | 25.10 [-12.40, 62.60] |
| Román, M. 2013 | -19.7 | 45.7 | 32 | -20.2 | 70.5 | 33 | 8.7% | 0.50 [-28.30, 29.30] |
| Souza, Y. 2018 | -11 | 108.5 | 24 | -6 | 82.16 | 24 | 5.6% | -5.00 [-59.45, 49.45] |
| Spencer, L.M. 2010 | 62 | 91.43 | 25 | 10 | 78.6 | 25 | 6.4% | 52.00 [4.74, 99.26] |
| Van Wetering, C.R. 2010 | -1.4 | 3.9 | 102 | -15.3 | 3.9 | 97 | 11.1% | 13.90 [12.82, 14.98] |
| **Total (95% CI)** | | | **549** | | | **574** | **100.0%** | **32.99 [14.55, 51.43]** |

Heterogeneity: Tau² = 796.19; Chi² = 70.21, df = 13 (P < 0.00001); I² = 81%
Test for overall effect: Z = 3.51 (P = 0.0005)

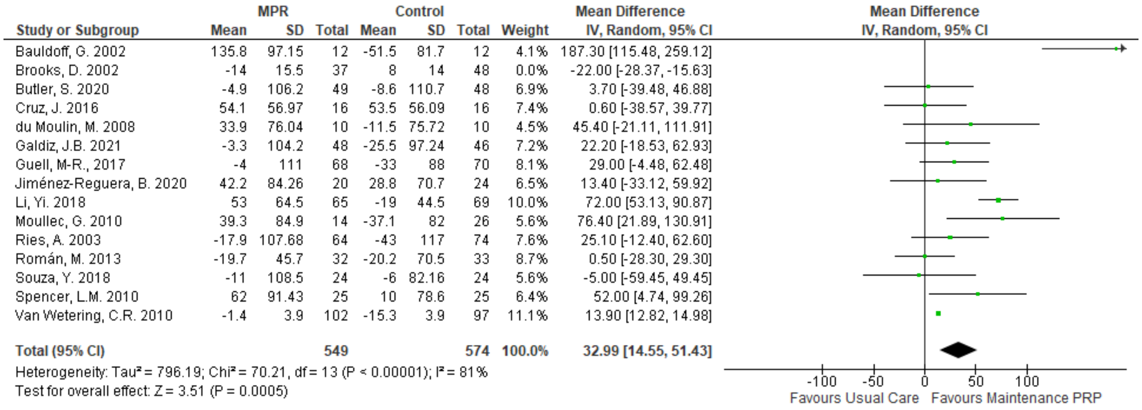

Subgroup analysis according to the main characteristics of the studies:

## Duration time of the maintenance PR programme

| Study or Subgroup | MPR Mean | MPR SD | MPR Total | Control Mean | Control SD | Control Total | Weight | Mean Difference IV, Random, 95% CI |
|---|---|---|---|---|---|---|---|---|
| **1.1.1 Up to 6 months** | | | | | | | | |
| Bauldoff, G. 2002 | 135.8 | 97.15 | 12 | -51.5 | 81.7 | 12 | 3.6% | 187.30 [115.48, 259.12] |
| Cruz, J. 2016 | 54.1 | 56.97 | 16 | 53.5 | 56.09 | 16 | 6.6% | 0.60 [-38.57, 39.77] |
| du Moulin, M. 2008 | 33.9 | 76.04 | 10 | -11.5 | 75.72 | 10 | 3.9% | 45.40 [-21.11, 111.91] |
| Souza, Y. 2018 | -11 | 108.5 | 24 | -6 | 82.16 | 24 | 4.9% | -5.00 [-59.45, 49.45] |
| **Subtotal (95% CI)** | | | **62** | | | **62** | **19.0%** | **53.41 [-23.28, 130.10]** |
| Heterogeneity: Tau² = 5229.11; Chi² = 22.36, df = 3 (P < 0.0001); I² = 87% | | | | | | | | |
| Test for overall effect: Z = 1.37 (P = 0.17) | | | | | | | | |
| **1.1.2 12 months** | | | | | | | | |
| Brooks, D. 2002 | -14 | 15.5 | 37 | 8 | 14 | 48 | 10.3% | -22.00 [-28.37, -15.63] |
| Butler, S. 2020 | -4.9 | 106.2 | 49 | -8.6 | 110.7 | 48 | 6.1% | 3.70 [-39.48, 46.88] |
| Galdiz, J.B. 2021 | -3.3 | 104.2 | 48 | -25.5 | 97.24 | 46 | 6.4% | 22.20 [-18.53, 62.93] |
| Jiménez-Reguera, B. 2020 | 42.2 | 84.26 | 20 | 28.8 | 70.7 | 24 | 5.8% | 13.40 [-33.12, 59.92] |
| Li, Yi. 2018 | 53 | 64.5 | 65 | -19 | 44.5 | 69 | 9.2% | 72.00 [53.13, 90.87] |
| Moullec, G. 2010 | 39.3 | 84.9 | 14 | -37.1 | 82 | 26 | 4.9% | 76.40 [21.89, 130.91] |
| Ries, A. 2003 | -17.9 | 107.68 | 64 | -43 | 117 | 74 | 6.8% | 25.10 [-12.40, 62.60] |
| Román, M. 2013 | -19.7 | 45.7 | 32 | -20.2 | 70.5 | 33 | 8.0% | 0.50 [-28.30, 29.30] |
| Spencer, L.M. 2010 | 62 | 91.43 | 25 | 10 | 78.6 | 25 | 5.7% | 52.00 [4.74, 99.26] |
| **Subtotal (95% CI)** | | | **354** | | | **393** | **63.2%** | **25.82 [-6.99, 58.62]** |
| Heterogeneity: Tau² = 2151.77; Chi² = 109.50, df = 8 (P < 0.00001); I² = 93% | | | | | | | | |
| Test for overall effect: Z = 1.54 (P = 0.12) | | | | | | | | |
| **1.1.3 More than 12 months** | | | | | | | | |
| Guell, M-R., 2017 | -4 | 111 | 68 | -33 | 88 | 70 | 7.3% | 29.00 [-4.48, 62.48] |
| Van Wetering, C.R. 2010 | -1.4 | 3.9 | 102 | -15.3 | 3.9 | 97 | 10.4% | 13.90 [12.82, 14.98] |
| **Subtotal (95% CI)** | | | **170** | | | **167** | **17.8%** | **13.92 [12.83, 15.00]** |
| Heterogeneity: Tau² = 0.00; Chi² = 0.78, df = 1 (P = 0.38); I² = 0% | | | | | | | | |
| Test for overall effect: Z = 25.17 (P < 0.00001) | | | | | | | | |
| **Total (95% CI)** | | | **586** | | | **622** | **100.0%** | **27.08 [10.39, 43.77]** |
| Heterogeneity: Tau² = 695.56; Chi² = 190.46, df = 14 (P < 0.00001); I² = 93% | | | | | | | | |
| Test for overall effect: Z = 3.18 (P = 0.001) | | | | | | | | |
| Test for subgroup differences: Chi² = 1.52, df = 2 (P = 0.47), I² = 0% | | | | | | | | |

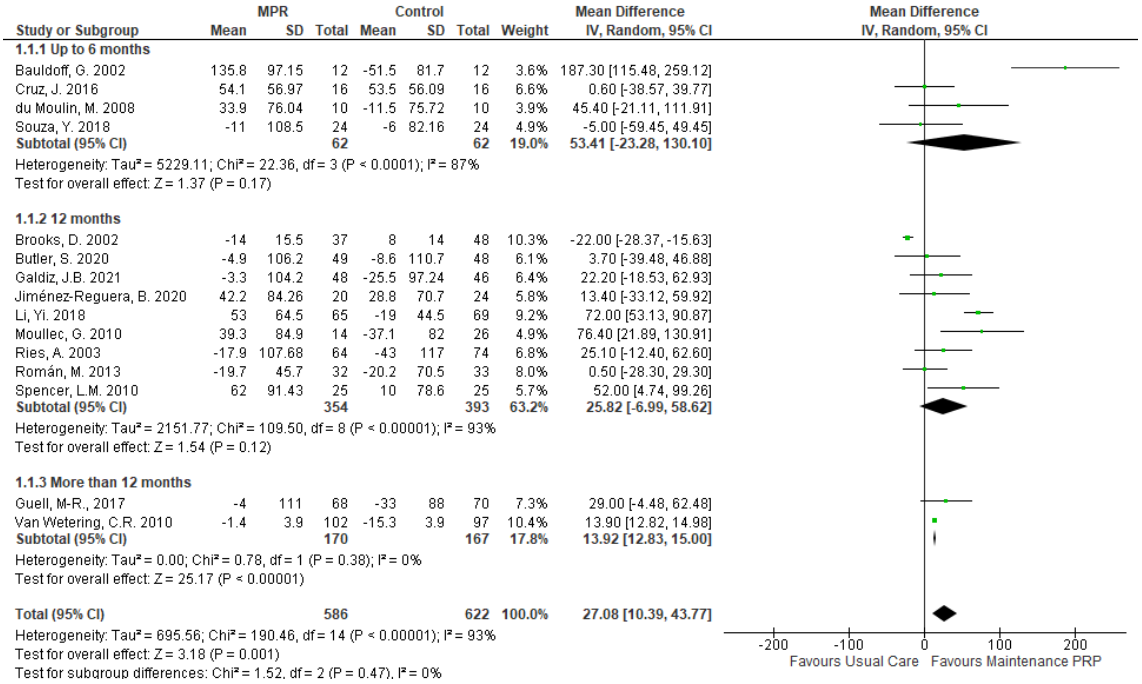


## Duration of the initial pulmonary rehabilitation programme

| Study or Subgroup | MPR Mean | MPR SD | MPR Total | Control Mean | Control SD | Control Total | Weight | Mean Difference IV, Random, 95% CI |
|---|---|---|---|---|---|---|---|---|
| **2.1.1 3 to 6 weeks** | | | | | | | | |
| Brooks, D. 2002 | -14 | 15.5 | 37 | 8 | 14 | 48 | 11.7% | -22.00 [-28.37, -15.63] |
| du Moulin, M. 2008 | 33.9 | 76.04 | 10 | -11.5 | 75.72 | 10 | 4.2% | 45.40 [-21.11, 111.91] |
| Moullec, G. 2010 | 39.3 | 84.9 | 14 | -37.1 | 82 | 26 | 5.3% | 76.40 [21.89, 130.91] |
| **Subtotal (95% CI)** | | | **61** | | | **84** | **21.2%** | **28.91 [-41.80, 99.63]** |
| Heterogeneity: Tau² = 3319.83; Chi² = 16.11, df = 2 (P = 0.0003); I² = 88% | | | | | | | | |
| Test for overall effect: Z = 0.80 (P = 0.42) | | | | | | | | |
| **2.1.2 8 weeks** | | | | | | | | |
| Galdiz, J.B. 2021 | -3.3 | 104.2 | 48 | -25.5 | 97.24 | 46 | 7.0% | 22.20 [-18.53, 62.93] |
| Guell, M-R., 2017 | -4 | 111 | 68 | -33 | 88 | 70 | 8.1% | 29.00 [-4.48, 62.48] |
| Jiménez-Reguera, B. 2020 | 42.2 | 84.26 | 20 | 28.8 | 70.7 | 24 | 6.3% | 13.40 [-33.12, 59.92] |
| Li, Yi. 2018 | 53 | 64.5 | 65 | -19 | 44.5 | 69 | 10.4% | 72.00 [53.13, 90.87] |
| Ries, A. 2003 | -17.9 | 107.68 | 64 | -43 | 117 | 74 | 7.5% | 25.10 [-12.40, 62.60] |
| Spencer, L.M. 2010 | 62 | 91.43 | 25 | 10 | 78.6 | 25 | 6.2% | 52.00 [4.74, 99.26] |
| **Subtotal (95% CI)** | | | **290** | | | **308** | **45.5%** | **38.74 [15.90, 61.57]** |
| Heterogeneity: Tau² = 464.24; Chi² = 12.56, df = 5 (P = 0.03); I² = 60% | | | | | | | | |
| Test for overall effect: Z = 3.32 (P = 0.0009) | | | | | | | | |
| **2.1.3 12 weeks** | | | | | | | | |
| Cruz, J. 2016 | 54.1 | 56.97 | 16 | 53.5 | 56.09 | 16 | 7.3% | 0.60 [-38.57, 39.77] |
| Román, M. 2013 | -19.7 | 45.7 | 32 | -20.2 | 70.5 | 33 | 8.8% | 0.50 [-28.30, 29.30] |
| Souza, Y. 2018 | -11 | 108.5 | 24 | -6 | 82.16 | 24 | 5.3% | -5.00 [-59.45, 49.45] |
| Van Wetering, C.R. 2010 | -1.4 | 3.9 | 102 | -15.3 | 3.9 | 97 | 11.9% | 13.90 [12.82, 14.98] |
| **Subtotal (95% CI)** | | | **174** | | | **170** | **33.3%** | **13.86 [12.78, 14.95]** |
| Heterogeneity: Tau² = 0.00; Chi² = 1.73, df = 3 (P = 0.63); I² = 0% | | | | | | | | |
| Test for overall effect: Z = 25.10 (P < 0.00001) | | | | | | | | |
| **Total (95% CI)** | | | **525** | | | **562** | **100.0%** | **22.18 [5.26, 39.11]** |
| Heterogeneity: Tau² = 627.03; Chi² = 167.69, df = 12 (P < 0.00001); I² = 93% | | | | | | | | |
| Test for overall effect: Z = 2.57 (P = 0.01) | | | | | | | | |
| Test for subgroup differences: Chi² = 4.72, df = 2 (P = 0.09), I² = 57.6% | | | | | | | | |

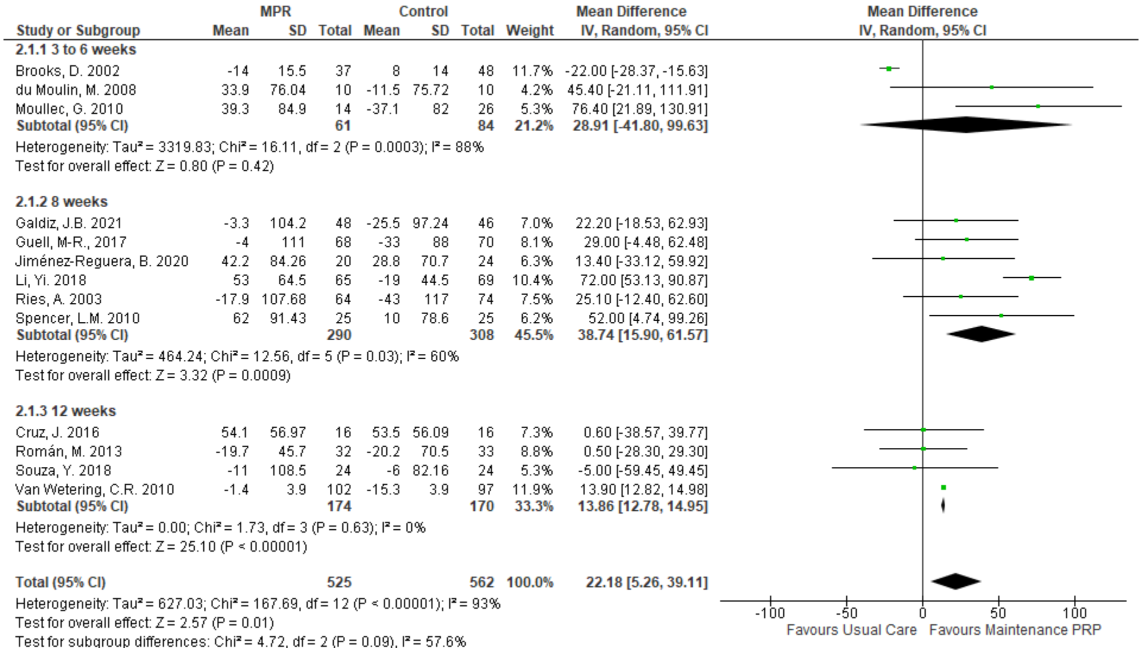

## Severity of COPD assessed by % $FEV_1$ predicted

| Study or Subgroup | MPR Mean | MPR SD | MPR Total | Control Mean | Control SD | Control Total | Weight | Mean Difference IV, Random, 95% CI |
|---|---|---|---|---|---|---|---|---|
| **3.1.1 Mild to Severe** | | | | | | | | |
| Cruz, J. 2016 | 54.1 | 56.97 | 16 | 53.5 | 56.09 | 16 | 7.7% | 0.60 [-38.57, 39.77] |
| Jiménez-Reguera, B. 2020 | 42.2 | 84.26 | 20 | 28.8 | 70.7 | 24 | 6.6% | 13.40 [-33.12, 59.92] |
| Román, M. 2013 | -19.7 | 45.7 | 32 | -20.2 | 70.5 | 33 | 9.3% | 0.50 [-28.30, 29.30] |
| Spencer, L.M. 2010 | 62 | 91.43 | 25 | 10 | 78.6 | 25 | 6.5% | 52.00 [4.74, 99.26] |
| Van Wetering, C.R. 2010 | -1.4 | 3.9 | 102 | -15.3 | 3.9 | 97 | 12.5% | 13.90 [12.82, 14.98] |
| **Subtotal (95% CI)** | | | **195** | | | **195** | **42.6%** | **13.89 [12.81, 14.97]** |
| Heterogeneity: Tau² = 0.00; Chi² = 3.77, df = 4 (P = 0.44); I² = 0% | | | | | | | | |
| Test for overall effect: Z = 25.15 (P < 0.00001) | | | | | | | | |
| **3.1.2 Moderate to Severe** | | | | | | | | |
| Brooks, D. 2002 | -14 | 15.5 | 37 | 8 | 14 | 48 | 12.3% | -22.00 [-28.37, -15.63] |
| Butler, S. 2020 | -4.9 | 106.2 | 49 | -8.6 | 110.7 | 48 | 7.1% | 3.70 [-39.48, 46.88] |
| Galdiz, J.B. 2021 | -3.3 | 104.2 | 48 | -25.5 | 97.24 | 46 | 7.4% | 22.20 [-18.53, 62.93] |
| Guell, M-R., 2017 | -4 | 111 | 68 | -33 | 88 | 70 | 8.5% | 29.00 [-4.48, 62.48] |
| Li, Yi. 2018 | 53 | 64.5 | 65 | -19 | 44.5 | 69 | 10.9% | 72.00 [53.13, 90.87] |
| Moullec, G. 2010 | 39.3 | 84.9 | 14 | -37.1 | 82 | 26 | 5.6% | 76.40 [21.89, 130.91] |
| Souza, Y. 2018 | -11 | 108.5 | 24 | -6 | 82.16 | 24 | 5.6% | -5.00 [-59.45, 49.45] |
| **Subtotal (95% CI)** | | | **305** | | | **331** | **57.4%** | **24.73 [-15.89, 65.34]** |
| Heterogeneity: Tau² = 2622.95; Chi² = 103.74, df = 6 (P < 0.00001); I² = 94% | | | | | | | | |
| Test for overall effect: Z = 1.19 (P = 0.23) | | | | | | | | |
| **Total (95% CI)** | | | **500** | | | **526** | **100.0%** | **19.64 [2.20, 37.07]** |
| Heterogeneity: Tau² = 634.14; Chi² = 166.57, df = 11 (P < 0.00001); I² = 93% | | | | | | | | |
| Test for overall effect: Z = 2.21 (P = 0.03) | | | | | | | | |
| Test for subgroup differences: Chi² = 0.27, df = 1 (P = 0.60), I² = 0% | | | | | | | | |

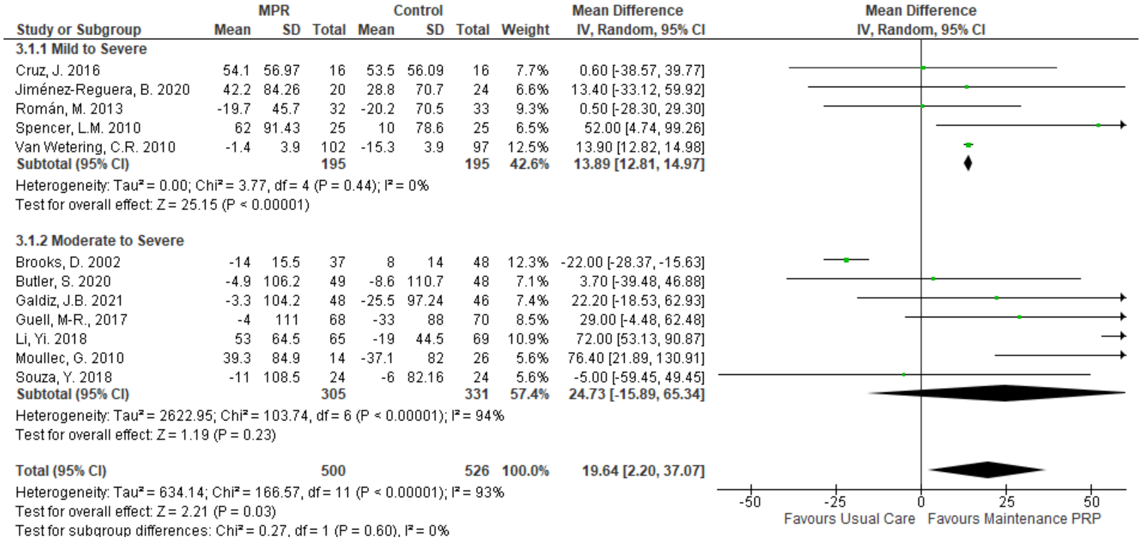


## Professional supervision

| Study or Subgroup | MPR Mean | MPR SD | MPR Total | Control Mean | Control SD | Control Total | Weight | Mean Difference IV, Random, 95% CI |
|---|---|---|---|---|---|---|---|---|
| **4.1.1 Supervised** | | | | | | | | |
| Bauldoff, G. 2002 | 135.8 | 97.15 | 12 | -51.5 | 81.7 | 12 | 3.6% | 187.30 [115.48, 259.12] |
| Li, Yi. 2018 | 53 | 64.5 | 65 | -19 | 44.5 | 69 | 9.2% | 72.00 [53.13, 90.87] |
| Moullec, G. 2010 | 39.3 | 84.9 | 14 | -37.1 | 82 | 26 | 4.9% | 76.40 [21.89, 130.91] |
| Román, M. 2013 | -19.7 | 45.7 | 32 | -20.2 | 70.5 | 33 | 8.0% | 0.50 [-28.30, 29.30] |
| **Subtotal (95% CI)** | | | **123** | | | **140** | **25.6%** | **76.66 [19.22, 134.09]** |
| Heterogeneity: Tau² = 2895.39; Chi² = 30.32, df = 3 (P < 0.00001); I² = 90% | | | | | | | | |
| Test for overall effect: Z = 2.62 (P = 0.009) | | | | | | | | |
| **4.1.2 Unsupervised** | | | | | | | | |
| Brooks, D. 2002 | -14 | 15.5 | 37 | 8 | 14 | 48 | 10.3% | -22.00 [-28.37, -15.63] |
| du Moulin, M. 2008 | 33.9 | 76.04 | 10 | -11.5 | 75.72 | 10 | 3.9% | 45.40 [-21.11, 111.91] |
| Galdiz, J.B. 2021 | -3.3 | 104.2 | 48 | -25.5 | 97.24 | 46 | 6.4% | 22.20 [-18.53, 62.93] |
| Jiménez-Reguera, B. 2020 | 42.2 | 84.26 | 20 | 28.8 | 70.7 | 24 | 5.8% | 13.40 [-33.12, 59.92] |
| Souza, Y. 2018 | -11 | 108.5 | 24 | -6 | 82.16 | 24 | 4.9% | -5.00 [-59.45, 49.45] |
| **Subtotal (95% CI)** | | | **139** | | | **152** | **31.3%** | **3.52 [-23.88, 30.93]** |
| Heterogeneity: Tau² = 538.50; Chi² = 10.47, df = 4 (P = 0.03); I² = 62% | | | | | | | | |
| Test for overall effect: Z = 0.25 (P = 0.80) | | | | | | | | |
| **4.1.3 Alternate supervision** | | | | | | | | |
| Butler, S. 2020 | -4.9 | 106.2 | 49 | -8.6 | 110.7 | 48 | 6.1% | 3.70 [-39.48, 46.88] |
| Cruz, J. 2016 | 54.1 | 56.97 | 16 | 53.5 | 56.09 | 16 | 6.6% | 0.60 [-38.57, 39.77] |
| Guell, M-R., 2017 | -4 | 111 | 68 | -33 | 88 | 70 | 7.3% | 29.00 [-4.48, 62.48] |
| Ries, A. 2003 | -17.9 | 107.68 | 64 | -43 | 117 | 74 | 6.8% | 25.10 [-12.40, 62.60] |
| Spencer, L.M. 2010 | 62 | 91.43 | 25 | 10 | 78.6 | 25 | 5.7% | 52.00 [4.74, 99.26] |
| Van Wetering, C.R. 2010 | -1.4 | 3.9 | 102 | -15.3 | 3.9 | 97 | 10.4% | 13.90 [12.82, 14.98] |
| **Subtotal (95% CI)** | | | **324** | | | **330** | **43.0%** | **13.93 [12.85, 15.01]** |
| Heterogeneity: Tau² = 0.00; Chi² = 4.27, df = 5 (P = 0.51); I² = 0% | | | | | | | | |
| Test for overall effect: Z = 25.23 (P < 0.00001) | | | | | | | | |
| **Total (95% CI)** | | | **586** | | | **622** | **100.0%** | **27.08 [10.39, 43.77]** |
| Heterogeneity: Tau² = 695.56; Chi² = 190.46, df = 14 (P < 0.00001); I² = 93% | | | | | | | | |
| Test for overall effect: Z = 3.18 (P = 0.001) | | | | | | | | |
| Test for subgroup differences: Chi² = 5.14, df = 2 (P = 0.08), I² = 61.1% | | | | | | | | |

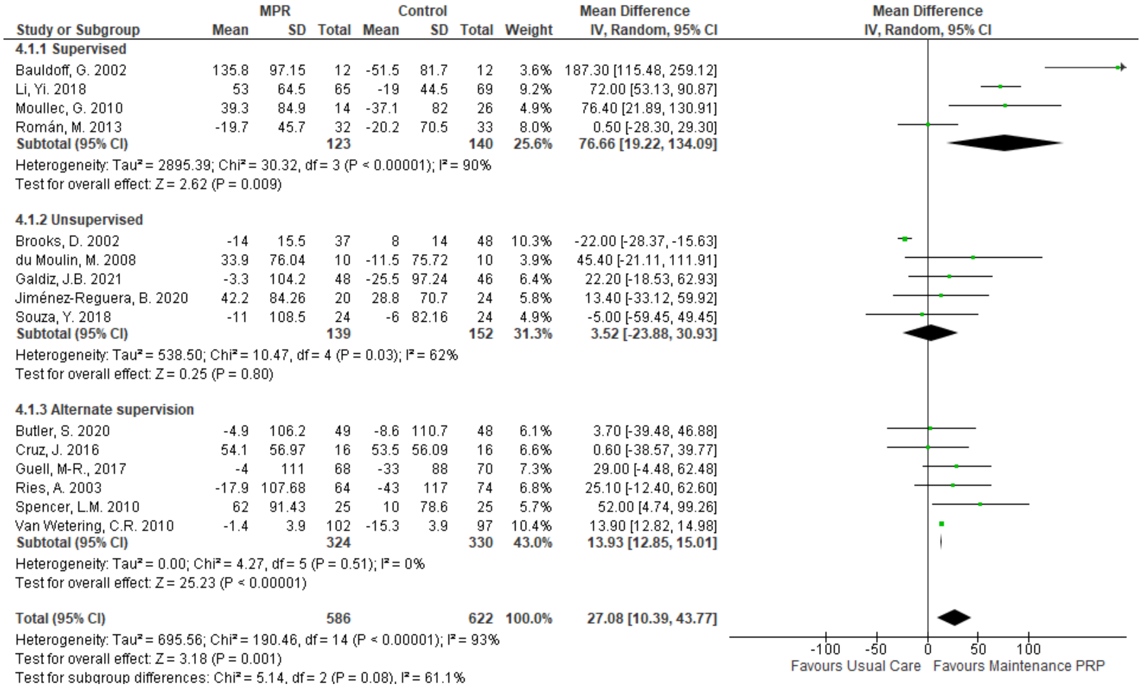

## Setting

| Study or Subgroup | MPR Mean | MPR SD | MPR Total | Control Mean | Control SD | Control Total | Weight | Mean Difference IV, Random, 95% CI |
|---|---|---|---|---|---|---|---|---|
| **14.1.1 Home-based (HB)** | | | | | | | | |
| Bauldoff, G. 2002 | 135.8 | 97.15 | 12 | -51.5 | 81.7 | 12 | 3.6% | 187.30 [115.48, 259.12] |
| du Moulin, M. 2008 | 33.9 | 76.04 | 10 | -11.5 | 75.72 | 10 | 3.9% | 45.40 [-21.11, 111.91] |
| Guell, M-R., 2017 | -4 | 111 | 68 | -33 | 88 | 70 | 7.3% | 29.00 [-4.48, 62.48] |
| Li, Yi. 2018 | 53 | 64.5 | 65 | -19 | 44.5 | 69 | 9.2% | 72.00 [53.13, 90.87] |
| Souza, Y. 2018 | -11 | 108.5 | 24 | -6 | 82.16 | 24 | 4.9% | -5.00 [-59.45, 49.45] |
| Van Wetering, C.R. 2010 | -1.4 | 3.9 | 102 | -15.3 | 3.9 | 97 | 10.4% | 13.90 [12.82, 14.98] |
| **Subtotal (95% CI)** | | | **281** | | | **282** | **39.4%** | **50.48 [13.59, 87.37]** |
| Heterogeneity: Tau² = 1632.99; Chi² = 60.71, df = 5 (P < 0.00001); I² = 92% | | | | | | | | |
| Test for overall effect: Z = 2.68 (P = 0.007) | | | | | | | | |
| **14.1.2 Community-Based (CB)** | | | | | | | | |
| Butler, S. 2020 | -4.9 | 106.2 | 49 | -8.6 | 110.7 | 48 | 6.1% | 3.70 [-39.48, 46.88] |
| Moullec, G. 2010 | 39.3 | 84.9 | 14 | -37.1 | 82 | 26 | 4.9% | 76.40 [21.89, 130.91] |
| Román, M. 2013 | -19.7 | 45.7 | 32 | -20.2 | 70.5 | 33 | 8.0% | 0.50 [-28.30, 29.30] |
| **Subtotal (95% CI)** | | | **95** | | | **107** | **19.0%** | **22.01 [-19.61, 63.63]** |
| Heterogeneity: Tau² = 898.77; Chi² = 6.10, df = 2 (P = 0.05); I² = 67% | | | | | | | | |
| Test for overall effect: Z = 1.04 (P = 0.30) | | | | | | | | |
| **14.1.3 Alternance HB and CB** | | | | | | | | |
| Brooks, D. 2002 | -14 | 15.5 | 37 | 8 | 14 | 48 | 10.3% | -22.00 [-28.37, -15.63] |
| Cruz, J. 2016 | 54.1 | 56.97 | 16 | 53.5 | 56.09 | 16 | 6.6% | 0.60 [-38.57, 39.77] |
| Ries, A. 2003 | -17.9 | 107.68 | 64 | -43 | 117 | 74 | 6.8% | 25.10 [-12.40, 62.60] |
| Spencer, L.M. 2010 | 62 | 91.43 | 25 | 10 | 78.6 | 25 | 5.7% | 52.00 [4.74, 99.26] |
| **Subtotal (95% CI)** | | | **142** | | | **163** | **29.4%** | **9.62 [-25.26, 44.50]** |
| Heterogeneity: Tau² = 964.21; Chi² = 15.79, df = 3 (P = 0.001); I² = 81% | | | | | | | | |
| Test for overall effect: Z = 0.54 (P = 0.59) | | | | | | | | |
| **14.1.4 Telerehabilitation** | | | | | | | | |
| Galdiz, J.B. 2021 | -3.3 | 104.2 | 48 | -25.5 | 97.24 | 46 | 6.4% | 22.20 [-18.53, 62.93] |
| Jiménez-Reguera, B. 2020 | 42.2 | 84.26 | 20 | 28.8 | 70.7 | 24 | 5.8% | 13.40 [-33.12, 59.92] |
| **Subtotal (95% CI)** | | | **68** | | | **70** | **12.2%** | **18.38 [-12.26, 49.02]** |
| Heterogeneity: Tau² = 0.00; Chi² = 0.08, df = 1 (P = 0.78); I² = 0% | | | | | | | | |
| Test for overall effect: Z = 1.18 (P = 0.24) | | | | | | | | |
| **Total (95% CI)** | | | **586** | | | **622** | **100.0%** | **27.08 [10.39, 43.77]** |
| Heterogeneity: Tau² = 695.56; Chi² = 190.46, df = 14 (P < 0.00001); I² = 93% | | | | | | | | |
| Test for overall effect: Z = 3.18 (P = 0.001) | | | | | | | | |
| Test for subgroup differences: Chi² = 2.77, df = 3 (P = 0.43), I² = 0% | | | | | | | | |

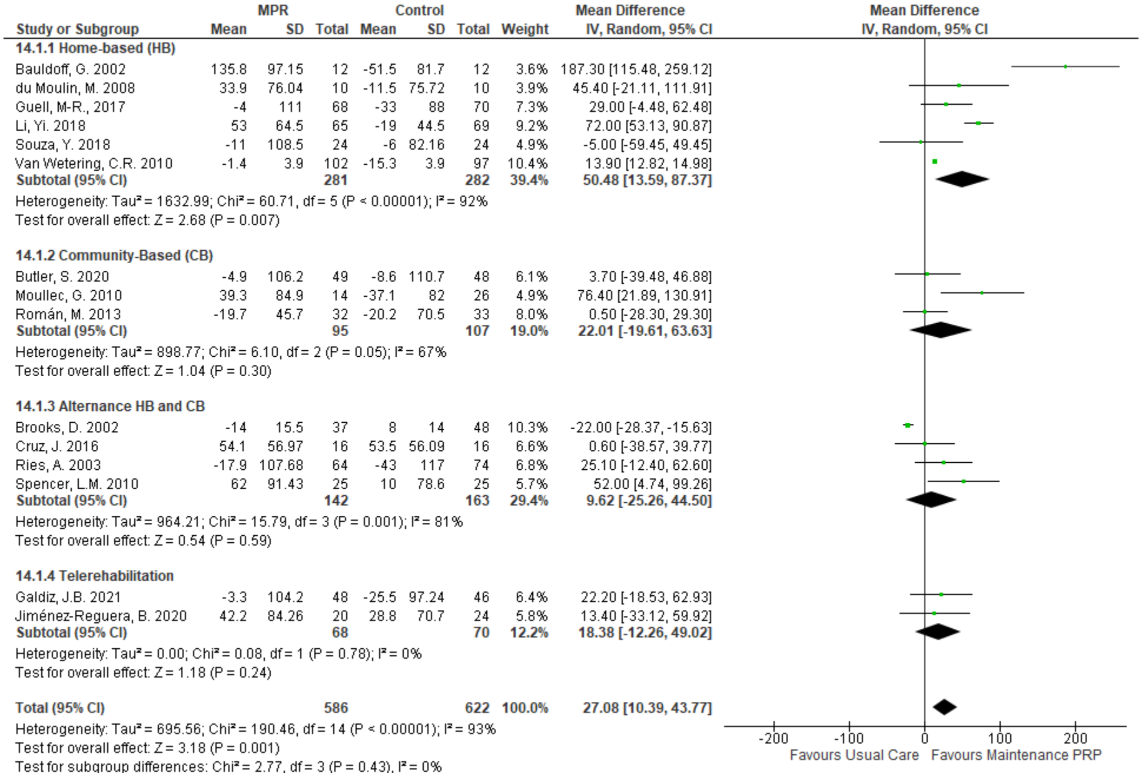


## Risk of bias

| Study or Subgroup | MPR Mean | MPR SD | MPR Total | Control Mean | Control SD | Control Total | Weight | Mean Difference IV, Random, 95% CI | A | B | C | D | E | F | G |
|---|---|---|---|---|---|---|---|---|---|---|---|---|---|---|---|
| **5.1.1 High risk of bias** | | | | | | | | | | | | | | | |
| Bauldoff, G. 2002 | 135.8 | 97.15 | 12 | -51.5 | 81.7 | 12 | 3.6% | 187.30 [115.48, 259.12] | ? | ? | − | ? | ? | − | + |
| Cruz, J. 2016 | 54.1 | 56.97 | 16 | 53.5 | 56.09 | 16 | 6.6% | 0.60 [-38.57, 39.77] | + | + | − | − | + | + | + |
| Moullec, G. 2010 | 39.3 | 84.9 | 14 | -37.1 | 82 | 26 | 4.9% | 76.40 [21.89, 130.91] | − | − | − | + | + | + | − |
| Ries, A. 2003 | -17.9 | 107.68 | 64 | -43 | 117 | 74 | 6.8% | 25.10 [-12.40, 62.60] | − | − | − | − | + | + | + |
| **Subtotal (95% CI)** | | | **106** | | | **128** | **21.9%** | **66.53 [0.85, 132.22]** | | | | | | | |
| Heterogeneity: Tau² = 3804.80; Chi² = 22.32, df = 3 (P < 0.0001); I² = 87% | | | | | | | | | | | | | | | |
| Test for overall effect: Z = 1.99 (P = 0.05) | | | | | | | | | | | | | | | |
| **5.1.2 Low risk of bias** | | | | | | | | | | | | | | | |
| Butler, S. 2020 | -4.9 | 106.2 | 49 | -8.6 | 110.7 | 48 | 6.1% | 3.70 [-39.48, 46.88] | + | + | − | + | + | + | + |
| du Moulin, M. 2008 | 33.9 | 76.04 | 10 | -11.5 | 75.72 | 10 | 3.9% | 45.40 [-21.11, 111.91] | + | + | − | + | + | + | + |
| Jiménez-Reguera, B. 2020 | 42.2 | 84.26 | 20 | 28.8 | 70.7 | 24 | 5.8% | 13.40 [-33.12, 59.92] | + | + | − | + | + | + | + |
| Van Wetering, C.R. 2010 | -1.4 | 3.9 | 102 | -15.3 | 3.9 | 97 | 10.4% | 13.90 [12.82, 14.98] | + | + | − | + | + | + | + |
| **Subtotal (95% CI)** | | | **181** | | | **179** | **26.2%** | **13.90 [12.82, 14.98]** | | | | | | | |
| Heterogeneity: Tau² = 0.00; Chi² = 1.08, df = 3 (P = 0.78); I² = 0% | | | | | | | | | | | | | | | |
| Test for overall effect: Z = 25.15 (P < 0.00001) | | | | | | | | | | | | | | | |
| **5.1.3 Uncertain risk of bias** | | | | | | | | | | | | | | | |
| Brooks, D. 2002 | -14 | 15.5 | 37 | 8 | 14 | 48 | 10.3% | -22.00 [-28.37, -15.63] | + | ? | − | + | ? | + | + |
| Galdiz, J.B. 2021 | -3.3 | 104.2 | 48 | -25.5 | 97.24 | 46 | 6.4% | 22.20 [-18.53, 62.93] | + | + | − | ? | + | + | + |
| Guell, M-R., 2017 | -4 | 111 | 68 | -33 | 88 | 70 | 7.3% | 29.00 [-4.48, 62.48] | + | ? | − | + | + | + | + |
| Li, Yi. 2018 | 53 | 64.5 | 65 | -19 | 44.5 | 69 | 9.2% | 72.00 [53.13, 90.87] | ? | ? | − | ? | + | + | + |
| Román, M. 2013 | -19.7 | 45.7 | 32 | -20.2 | 70.5 | 33 | 8.0% | 0.50 [-28.30, 29.30] | + | + | − | ? | + | + | + |
| Souza, Y. 2018 | -11 | 108.5 | 24 | -6 | 82.16 | 24 | 4.9% | -5.00 [-59.45, 49.45] | + | + | − | ? | + | + | + |
| Spencer, L.M. 2010 | 62 | 91.43 | 25 | 10 | 78.6 | 25 | 5.7% | 52.00 [4.74, 99.26] | + | + | − | ? | + | + | + |
| **Subtotal (95% CI)** | | | **299** | | | **315** | **51.8%** | **21.14 [-16.42, 58.70]** | | | | | | | |
| Heterogeneity: Tau² = 2252.29; Chi² = 100.79, df = 6 (P < 0.00001); I² = 94% | | | | | | | | | | | | | | | |
| Test for overall effect: Z = 1.10 (P = 0.27) | | | | | | | | | | | | | | | |
| **Total (95% CI)** | | | **586** | | | **622** | **100.0%** | **27.08 [10.39, 43.77]** | | | | | | | |
| Heterogeneity: Tau² = 695.56; Chi² = 190.46, df = 14 (P < 0.00001); I² = 93% | | | | | | | | | | | | | | | |
| Test for overall effect: Z = 3.18 (P = 0.001) | | | | | | | | | | | | | | | |
| Test for subgroup differences: Chi² = 2.61, df = 2 (P = 0.27), I² = 23.3% | | | | | | | | | | | | | | | |

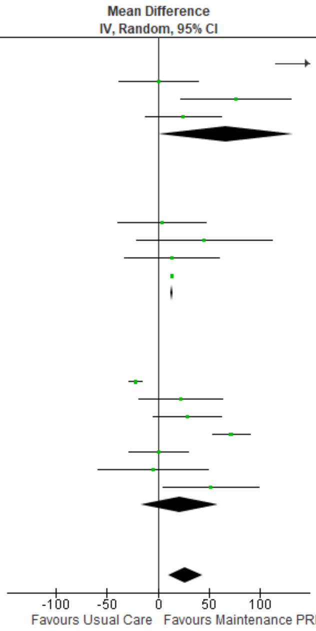


Risk of bias legend
(A) Random sequence generation (selection bias)
(B) Allocation concealment (selection bias)
(C) Blinding of participants and personnel (performance bias)
(D) Blinding of outcome assessment (detection bias)
(E) Incomplete outcome data (attrition bias)
(F) Selective reporting (reporting bias)
(G) Other bias

## Year of publication

| Study or Subgroup | MPR Mean | MPR SD | MPR Total | Control Mean | Control SD | Control Total | Weight | Mean Difference IV, Random, 95% CI |
|---|---|---|---|---|---|---|---|---|
| **6.1.1 Until 2013** | | | | | | | | |
| Bauldoff, G. 2002 | 135.8 | 97.15 | 12 | -51.5 | 81.7 | 12 | 3.6% | 187.30 [115.48, 259.12] |
| Brooks, D. 2002 | -14 | 15.5 | 37 | 8 | 14 | 48 | 10.3% | -22.00 [-28.37, -15.63] |
| du Moulin, M. 2008 | 33.9 | 76.04 | 10 | -11.5 | 75.72 | 10 | 3.9% | 45.40 [-21.11, 111.91] |
| Moullec, G. 2010 | 39.3 | 84.9 | 14 | -37.1 | 82 | 26 | 4.9% | 76.40 [21.89, 130.91] |
| Ries, A. 2003 | -17.9 | 107.68 | 64 | -43 | 117 | 74 | 6.8% | 25.10 [-12.40, 62.60] |
| Román, M. 2013 | -19.7 | 45.7 | 32 | -20.2 | 70.5 | 33 | 8.0% | 0.50 [-28.30, 29.30] |
| Spencer, L.M. 2010 | 62 | 91.43 | 25 | 10 | 78.6 | 25 | 5.7% | 52.00 [4.74, 99.26] |
| Van Wetering, C.R. 2010 | -1.4 | 3.9 | 102 | -15.3 | 3.9 | 97 | 10.4% | 13.90 [12.82, 14.98] |
| **Subtotal (95% CI)** | | | **296** | | | **325** | **53.6%** | **30.07 [7.27, 52.87]** |
| Heterogeneity: Tau² = 695.19; Chi² = 150.94, df = 7 (P < 0.00001); I² = 95% | | | | | | | | |
| Test for overall effect: Z = 2.59 (P = 0.010) | | | | | | | | |
| **6.1.2 From 2014** | | | | | | | | |
| Butler, S. 2020 | -4.9 | 106.2 | 49 | -8.6 | 110.7 | 48 | 6.1% | 3.70 [-39.48, 46.88] |
| Cruz, J. 2016 | 54.1 | 56.97 | 16 | 53.5 | 56.09 | 16 | 6.6% | 0.60 [-38.57, 39.77] |
| Galdiz, J.B. 2021 | -3.3 | 104.2 | 48 | -25.5 | 97.24 | 46 | 6.4% | 22.20 [-18.53, 62.93] |
| Guell, M-R., 2017 | -4 | 111 | 68 | -33 | 88 | 70 | 7.3% | 29.00 [-4.48, 62.48] |
| Jiménez-Reguera, B. 2020 | 42.2 | 84.26 | 20 | 28.8 | 70.7 | 24 | 5.8% | 13.40 [-33.12, 59.92] |
| Li, Yi. 2018 | 53 | 64.5 | 65 | -19 | 44.5 | 69 | 9.2% | 72.00 [53.13, 90.87] |
| Souza, Y. 2018 | -11 | 108.5 | 24 | -6 | 82.16 | 24 | 4.9% | -5.00 [-59.45, 49.45] |
| **Subtotal (95% CI)** | | | **290** | | | **297** | **46.4%** | **22.80 [-4.22, 49.81]** |
| Heterogeneity: Tau² = 925.70; Chi² = 22.67, df = 6 (P = 0.0009); I² = 74% | | | | | | | | |
| Test for overall effect: Z = 1.65 (P = 0.10) | | | | | | | | |
| **Total (95% CI)** | | | **586** | | | **622** | **100.0%** | **27.08 [10.39, 43.77]** |
| Heterogeneity: Tau² = 695.56; Chi² = 190.46, df = 14 (P < 0.00001); I² = 93% | | | | | | | | |
| Test for overall effect: Z = 3.18 (P = 0.001) | | | | | | | | |
| Test for subgroup differences: Chi² = 0.16, df = 1 (P = 0.69), I² = 0% | | | | | | | | |

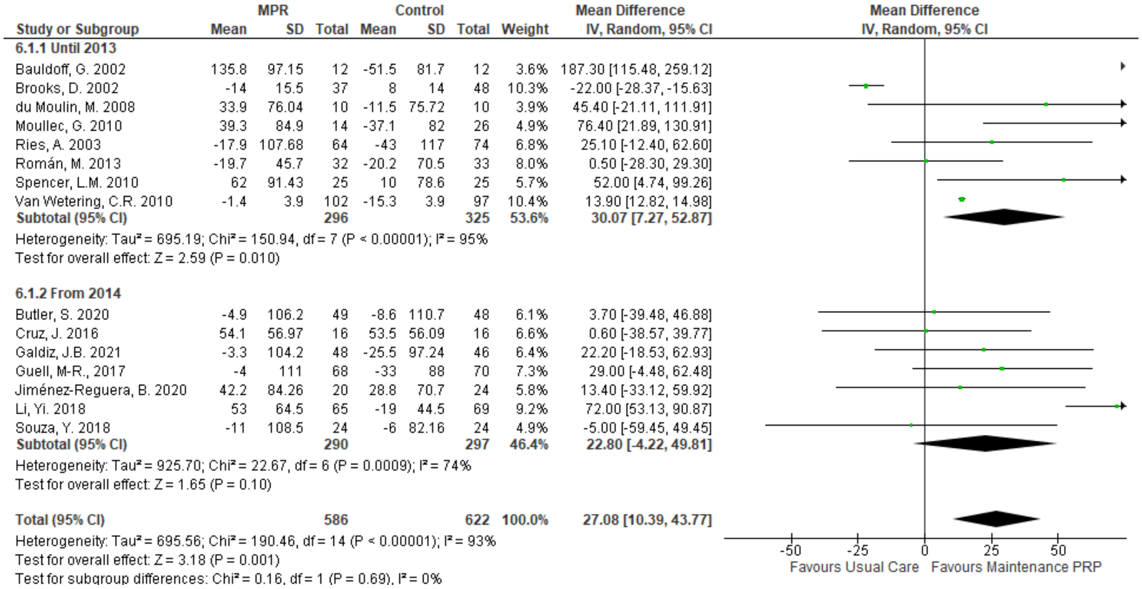


## Intervention

| Study or Subgroup | MPR Mean | MPR SD | MPR Total | Control Mean | Control SD | Control Total | Weight | Mean Difference IV, Random, 95% CI |
|---|---|---|---|---|---|---|---|---|
| **17.1.1 Exercise and Education** | | | | | | | | |
| Galdiz, J.B. 2021 | -3.3 | 104.2 | 48 | -25.5 | 97.24 | 46 | 6.4% | 22.20 [-18.53, 62.93] |
| Jiménez-Reguera, B. 2020 | 42.2 | 84.26 | 20 | 28.8 | 70.7 | 24 | 5.8% | 13.40 [-33.12, 59.92] |
| Li, Yi. 2018 | 53 | 64.5 | 65 | -19 | 44.5 | 69 | 9.2% | 72.00 [53.13, 90.87] |
| Moullec, G. 2010 | 39.3 | 84.9 | 14 | -37.1 | 82 | 26 | 4.9% | 76.40 [21.89, 130.91] |
| Van Wetering, C.R. 2010 | -1.4 | 3.9 | 102 | -15.3 | 3.9 | 97 | 10.4% | 13.90 [12.82, 14.98] |
| **Subtotal (95% CI)** | | | **249** | | | **262** | **36.7%** | **38.60 [4.89, 72.31]** |
| Heterogeneity: Tau² = 1162.55; Chi² = 41.46, df = 4 (P < 0.00001); I² = 90% | | | | | | | | |
| Test for overall effect: Z = 2.24 (P = 0.02) | | | | | | | | |
| **17.1.2 Exercise alone** | | | | | | | | |
| Bauldoff, G. 2002 | 135.8 | 97.15 | 12 | -51.5 | 81.7 | 12 | 3.6% | 187.30 [115.48, 259.12] |
| Brooks, D. 2002 | -14 | 15.5 | 37 | 8 | 14 | 48 | 10.3% | -22.00 [-28.37, -15.63] |
| Butler, S. 2020 | -4.9 | 106.2 | 49 | -8.6 | 110.7 | 48 | 6.1% | 3.70 [-39.48, 46.88] |
| Cruz, J. 2016 | 54.1 | 56.97 | 16 | 53.5 | 56.09 | 16 | 6.6% | 0.60 [-38.57, 39.77] |
| du Moulin, M. 2008 | 33.9 | 76.04 | 10 | -11.5 | 75.72 | 10 | 3.9% | 45.40 [-21.11, 111.91] |
| Guell, M-R., 2017 | -4 | 111 | 68 | -33 | 88 | 70 | 7.3% | 29.00 [-4.48, 62.48] |
| Ries, A. 2003 | -17.9 | 107.68 | 64 | -43 | 117 | 74 | 6.8% | 25.10 [-12.40, 62.60] |
| Román, M. 2013 | -19.7 | 45.7 | 32 | -20.2 | 70.5 | 33 | 8.0% | 0.50 [-28.30, 29.30] |
| Souza, Y. 2018 | -11 | 108.5 | 24 | -6 | 82.16 | 24 | 4.9% | -5.00 [-59.45, 49.45] |
| Spencer, L.M. 2010 | 62 | 91.43 | 25 | 10 | 78.6 | 25 | 5.7% | 52.00 [4.74, 99.26] |
| **Subtotal (95% CI)** | | | **337** | | | **360** | **63.3%** | **24.27 [-2.70, 51.25]** |
| Heterogeneity: Tau² = 1404.79; Chi² = 59.54, df = 9 (P < 0.00001); I² = 85% | | | | | | | | |
| Test for overall effect: Z = 1.76 (P = 0.08) | | | | | | | | |
| **Total (95% CI)** | | | **586** | | | **622** | **100.0%** | **27.08 [10.39, 43.77]** |
| Heterogeneity: Tau² = 695.56; Chi² = 190.46, df = 14 (P < 0.00001); I² = 93% | | | | | | | | |
| Test for overall effect: Z = 3.18 (P = 0.001) | | | | | | | | |
| Test for subgroup differences: Chi² = 0.42, df = 1 (P = 0.52), I² = 0% | | | | | | | | |

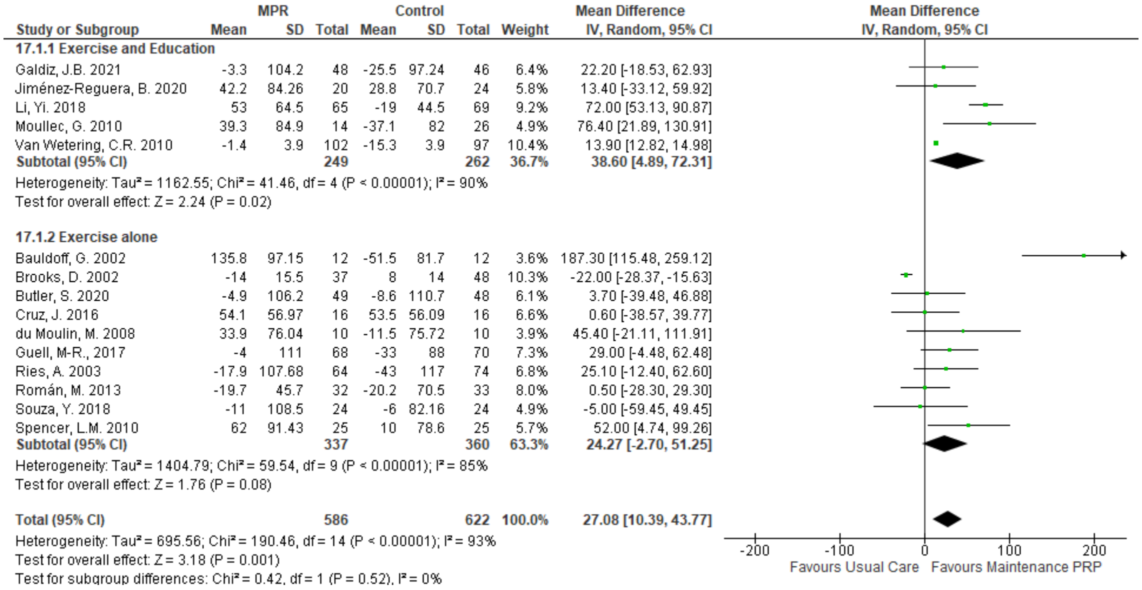

**Funnel plot – all studies**

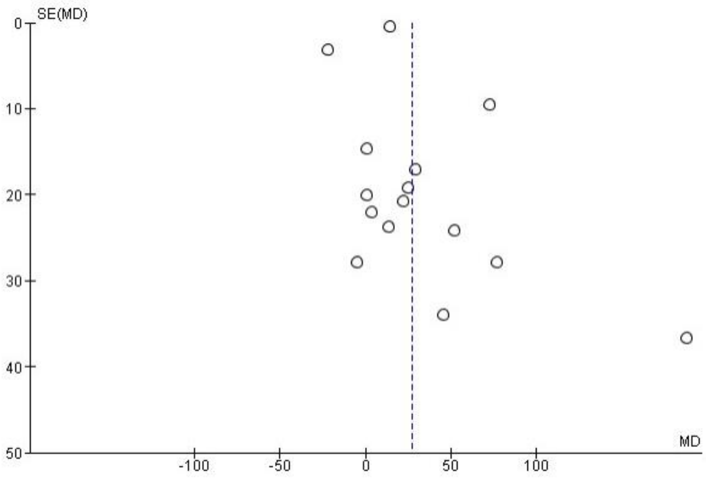


```
> metabias(rvmeta,method="rank")
Rank correlation test of funnel plot asymmetry

Test result: z = 0.64, p-value = 0.5200

Sample estimates:
      ks    se.ks
 13.0000 20.2073

- reference: Begg & Mazumdar (1993), Biometrics
>
```

## Meta regression

## Six minute walking test (6MWT) adjusted to FEV1 mean value:

```
> rvmeta_FEV1

Mixed-Effects Model (k = 15; tau^2 estimator: DL)

tau^2 (estimated amount of residual heterogeneity):     1478.6826 (SE = 1041.4222)
tau (square root of estimated tau^2 value):             38.4536
I^2 (residual heterogeneity / unaccounted variability): 87.63%
H^2 (unaccounted variability / sampling variability):   8.08
R^2 (amount of heterogeneity accounted for):            0.00%

Test for Residual Heterogeneity:
QE(df = 13) = 105.0627, p-val < .0001

Test of Moderators (coefficient 2):
QM(df = 1) = 0.0401, p-val = 0.8412

Model Results:

         estimate       se     zval    pval     ci.lb     ci.ub
intrcpt   40.5054  55.8947   0.7247  0.4687  -69.0463  150.0570
FEV_1     -0.2226   1.1109  -0.2003  0.8412   -2.3999    1.9548

---
Signif. codes:  0 '***' 0.001 '**' 0.01 '*' 0.05 '.' 0.1 ' ' 1
```

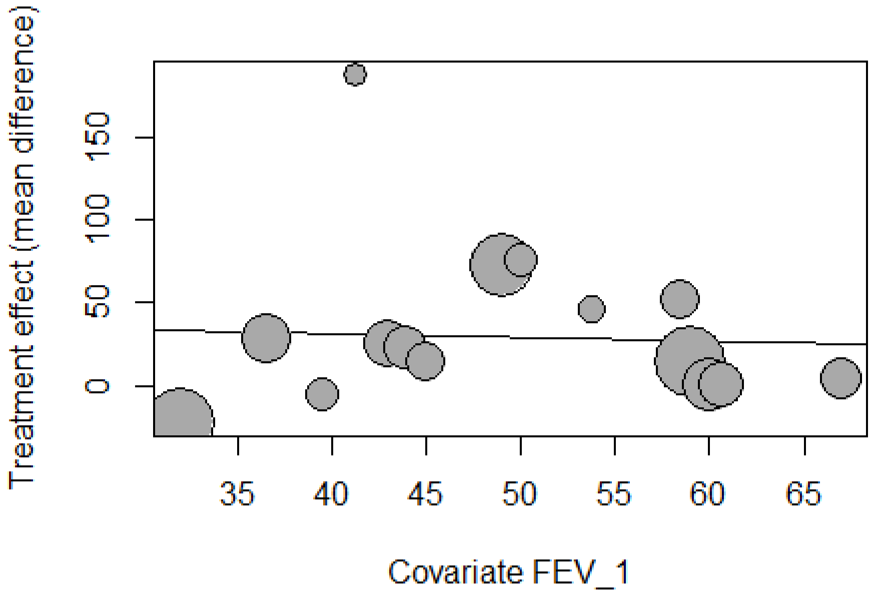

**Six minute walking test (6MWT) adjusted to time of maintenance programme in months:**

```
> rvmeta_TempoPM

Mixed-Effects Model (k = 15; tau^2 estimator: DL)

tau^2 (estimated amount of residual heterogeneity):     1335.3799 (SE = 1120.4054)
tau (square root of estimated tau^2 value):             36.5428
I^2 (residual heterogeneity / unaccounted variability): 91.21%
H^2 (unaccounted variability / sampling variability):   11.38
R^2 (amount of heterogeneity accounted for):            0.00%

Test for Residual Heterogeneity:
QE(df = 13) = 147.9317, p-val < .0001

Test of Moderators (coefficient 2):
QM(df = 1) = 0.0241, p-val = 0.8767

Model Results:

         estimate       se     zval    pval     ci.lb    ci.ub
intrcpt   32.1193  21.5412   1.4911  0.1359  -10.1006  74.3393
Time_MP   -0.2237   1.4416  -0.1552  0.8767   -3.0492   2.6018

---
Signif. codes:  0 ‘***’ 0.001 ‘**’ 0.01 ‘*’ 0.05 ‘.’ 0.1 ‘ ’ 1

> 
```

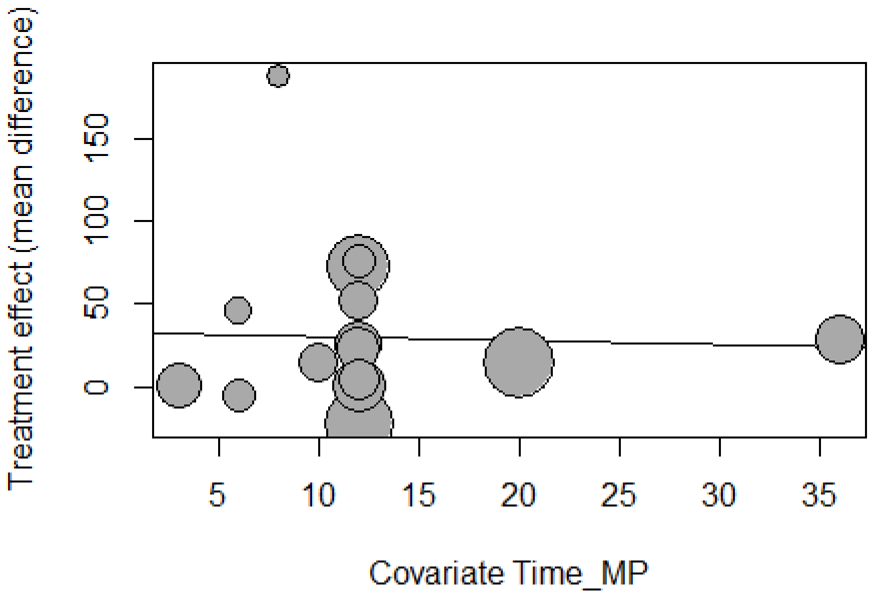

## Six minute walking test (6MWT) adjusted to supervision

## (reference – unsupervised)

```
> rvmeta_SV

Mixed-Effects Model (k = 15; tau^2 estimator: DL)

tau^2 (estimated amount of residual heterogeneity):     714.4834 (SE = 496.6212)
tau (square root of estimated tau^2 value):             26.7298
I^2 (residual heterogeneity / unaccounted variability): 73.37%
H^2 (unaccounted variability / sampling variability):   3.76
R^2 (amount of heterogeneity accounted for):            0.00%

Test for Residual Heterogeneity:
QE(df = 12) = 45.0614, p-val < .0001

Test of Moderators (coefficients 2:3):
QM(df = 2) = 7.9036, p-val = 0.0192

Model Results:

                                  estimate       se    zval    pval     ci.lb     ci.ub
intrcpt                             4.8263  15.3520  0.3144  0.7532  -25.2630   34.9157
Supervisionalternate supervision   14.9712  20.1862  0.7417  0.4583  -24.5929   54.5354
Supervisionsupervised              62.0328  22.8848  2.7107  0.0067   17.1794  106.8861  **

---
Signif. codes:  0 '***' 0.001 '**' 0.01 '*' 0.05 '.' 0.1 ' ' 1

>
```

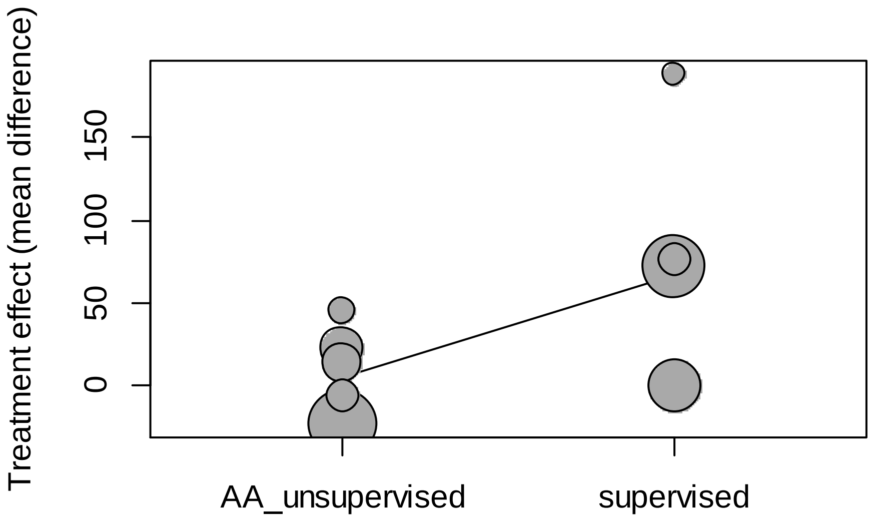

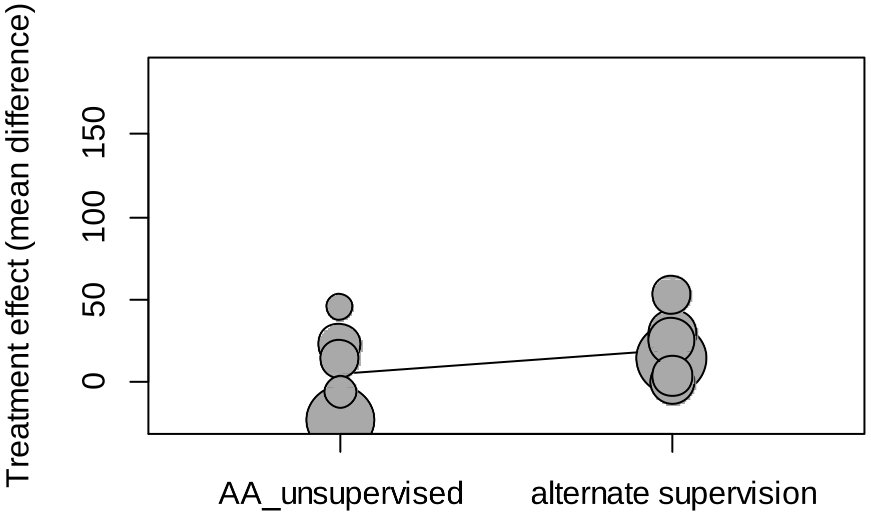
Treatment effect (mean difference)
150
100
50
0
AA_unsupervised
alternate supervision

**Six minute walking test (6MWT) adjusted to setting**

**(reference – community-based)**

```
> rvmeta_Set

Mixed-Effects Model (k = 15; tau^2 estimator: DL)

tau^2 (estimated amount of residual heterogeneity):     1328.5748 (SE = 1027.0420)
tau (square root of estimated tau^2 value):             36.4496
I^2 (residual heterogeneity / unaccounted variability): 86.70%
H^2 (unaccounted variability / sampling variability):   7.52
R^2 (amount of heterogeneity accounted for):            0.00%

Test for Residual Heterogeneity:
QE(df = 11) = 82.6775, p-val < .0001

Test of Moderators (coefficients 2:4):
QM(df = 3) = 2.4268, p-val = 0.4887

Model Results:

                             estimate       se     zval    pval     ci.lb    ci.ub
intrcpt                       23.1173  24.4378   0.9460  0.3442  -24.7800  71.0146
SettingHD and CB             -12.4763  31.7365  -0.3931  0.6942  -74.6788  49.7261
SettingHome-based             26.4951  29.9631   0.8843  0.3766  -32.2316  85.2217
SettingTelerehabilitation     -5.1588  38.8467  -0.1328  0.8944  -81.2970  70.9793

---
Signif. codes:  0 ‘***’ 0.001 ‘**’ 0.01 ‘*’ 0.05 ‘.’ 0.1 ‘ ’ 1

>
```

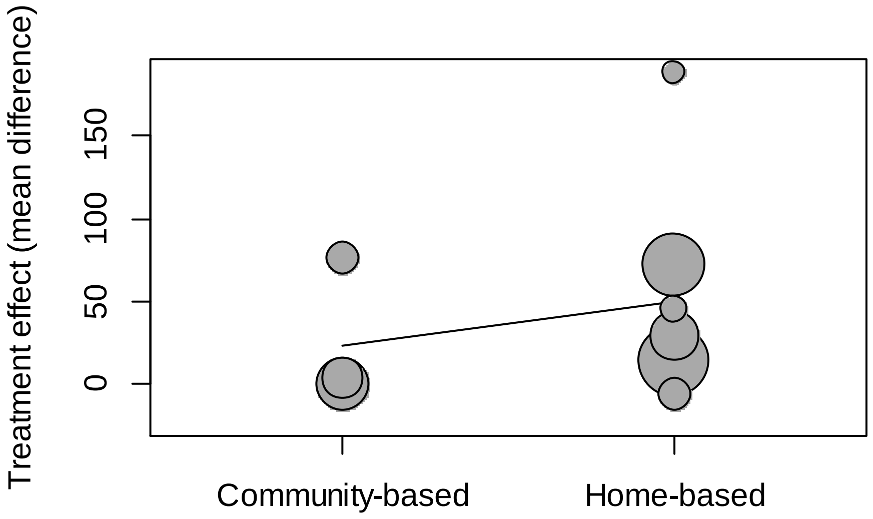

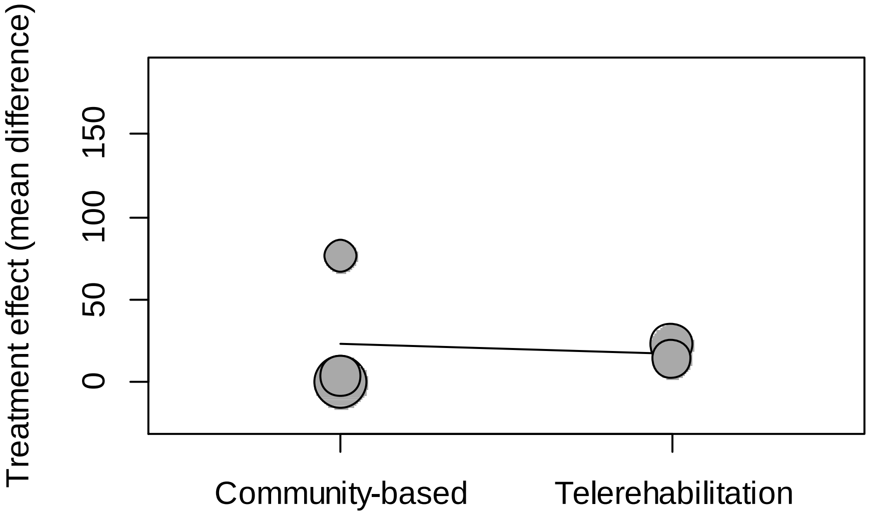
Treatment effect (mean difference)
0
50
100
150
Community-based
Telerehabilitation

## Six minute walking test (6MWT) adjusted to age (in years)

```
> View(rvmeta_Age)
> rvmeta_Age

Mixed-Effects Model (k = 15; tau^2 estimator: DL)

tau^2 (estimated amount of residual heterogeneity):     388.1607 (SE = 433.8453)
tau (square root of estimated tau^2 value):             19.7018
I^2 (residual heterogeneity / unaccounted variability): 83.24%
H^2 (unaccounted variability / sampling variability):   5.97
R^2 (amount of heterogeneity accounted for):            44.19%

Test for Residual Heterogeneity:
QE(df = 13) = 77.5703, p-val < .0001

Test of Moderators (coefficient 2):
QM(df = 1) = 2.9059, p-val = 0.0883

Model Results:

         estimate        se     zval    pval      ci.lb     ci.ub
intrcpt  447.6759  248.0795   1.8046  0.0711  -38.5509  933.9028  .
Age       -6.3842    3.7451  -1.7047  0.0883  -13.7244    0.9561  .

---
Signif. codes:  0 ‘***’ 0.001 ‘**’ 0.01 ‘*’ 0.05 ‘.’ 0.1 ‘ ’ 1
```

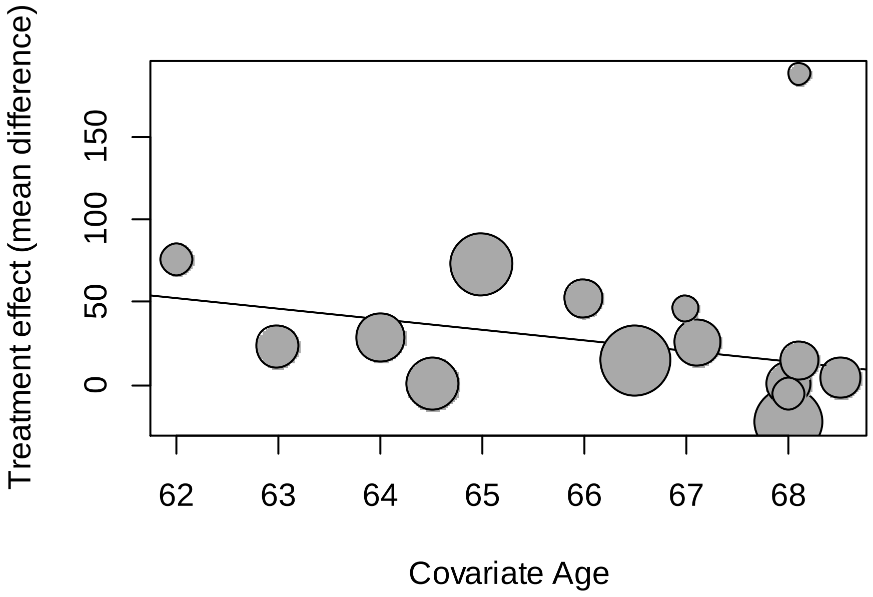

## Six minute walking test (6MWT) adjusted to supervision (reference: unsupervised) and FEV1

```
> rvmeta_Sup_FEV1

Mixed-Effects Model (k = 15; tau^2 estimator: DL)

tau^2 (estimated amount of residual heterogeneity):     830.6380 (SE = 580.1543)
tau (square root of estimated tau^2 value):             28.8208
I^2 (residual heterogeneity / unaccounted variability): 74.89%
H^2 (unaccounted variability / sampling variability):   3.98
R^2 (amount of heterogeneity accounted for):            0.00%

Test for Residual Heterogeneity:
QE(df = 11) = 43.8079, p-val < .0001

Test of Moderators (coefficients 2:4):
QM(df = 3) = 8.1503, p-val = 0.0430

Model Results:

                                  estimate       se     zval    pval     ci.lb     ci.ub
intrcpt                            45.6643  46.0607   0.9914  0.3215  -44.6131  135.9416
Supervisionalternate supervision   27.1075  25.2477   1.0737  0.2830  -22.3771   76.5921
Supervisionsupervised              72.7657  26.4906   2.7468  0.0060   20.8451  124.6864  **
FEV_1                              -0.9807   1.0516  -0.9325  0.3511   -3.0418    1.0805

---
Signif. codes:  0 ‘***’ 0.001 ‘**’ 0.01 ‘*’ 0.05 ‘.’ 0.1 ‘ ’ 1
```

## Six minute walking test (6MWT) adjusted to setting (reference: community-based) and FEV1

```
> rvmeta_Set_FEV1

Mixed-Effects Model (k = 15; tau^2 estimator: DL)

tau^2 (estimated amount of residual heterogeneity):     1728.5818 (SE = 1322.9518)
tau (square root of estimated tau^2 value):             41.5762
I^2 (residual heterogeneity / unaccounted variability): 87.51%
H^2 (unaccounted variability / sampling variability):   8.00
R^2 (amount of heterogeneity accounted for):            0.00%

Test for Residual Heterogeneity:
QE(df = 10) = 80.0469, p-val < .0001

Test of Moderators (coefficients 2:5):
QM(df = 4) = 2.0401, p-val = 0.7284

Model Results:

                           estimate       se     zval    pval      ci.lb     ci.ub
intrcpt                     34.5896  85.8758   0.4028  0.6871  -133.7239  202.9031
SettingHD and CB           -14.6346  38.8992  -0.3762  0.7068   -90.8755   61.6064
SettingHome-based           24.7086  37.2231   0.6638  0.5068   -48.2473   97.6645
SettingTelerehabilitation   -8.5562  47.5660  -0.1799  0.8572  -101.7838   84.6714
FEV_1                       -0.1822   1.3727  -0.1327  0.8944    -2.8727    2.5083

---
Signif. codes:  0 ‘***’ 0.001 ‘**’ 0.01 ‘*’ 0.05 ‘.’ 0.1 ‘ ’ 1

>
```

## Six minute walking test (6MWT) adjusted to setting (reference: community-based) and supervision (reference: unsupervised)

```
> rvmeta_Set_Sup <- metareg(rvmeta2,Setting+Supervision)
> rvmeta_Set_Sup

Mixed-Effects Model (k = 15; tau^2 estimator: DL)

tau^2 (estimated amount of residual heterogeneity):     664.0524 (SE = 536.1438)
tau (square root of estimated tau^2 value):             25.7692
I^2 (residual heterogeneity / unaccounted variability): 66.81%
H^2 (unaccounted variability / sampling variability):   3.01
R^2 (amount of heterogeneity accounted for):            4.53%

Test for Residual Heterogeneity:
QE(df = 9) = 27.1126, p-val = 0.0013

Test of Moderators (coefficients 2:6):
QM(df = 5) = 13.2692, p-val = 0.0210

Model Results:

                                 estimate       se     zval    pval     ci.lb     ci.ub
intrcpt                          -48.5169  31.8595  -1.5228  0.1278  -110.9603   13.9265
SettingHD and CB                  37.3966  29.7211   1.2582  0.2083   -20.8558   95.6489
SettingHome-based                 52.8433  25.0633   2.1084  0.0350     3.7202  101.9665   *
SettingTelerehabilitation         66.5660  39.9242   1.6673  0.0955   -11.6841  144.8160   .
Supervisionalternate supervision  29.7473  23.3166   1.2758  0.2020   -15.9524   75.4470
Supervisionsupervised             88.5050  29.6398   2.9860  0.0028    30.4121  146.5979  **

---
Signif. codes:  0 '***' 0.001 '**' 0.01 '*' 0.05 '.' 0.1 ' ' 1
```

## Six minute walking test (6MWT) adjusted to setting (reference: community-based), supervision (reference: unsupervised) and FEV1

```
> rvmeta_Set_Sup_FEV1 <- metareg(rvmeta2,Setting+Supervision+FEV_1)
> rvmeta_Set_Sup_FEV1

Mixed-Effects Model (k = 15; tau^2 estimator: DL)

tau^2 (estimated amount of residual heterogeneity):     785.0590 (SE = 636.3181)
tau (square root of estimated tau^2 value):             28.0189
I^2 (residual heterogeneity / unaccounted variability): 68.82%
H^2 (unaccounted variability / sampling variability):   3.21
R^2 (amount of heterogeneity accounted for):            0.00%

Test for Residual Heterogeneity:
QE(df = 8) = 25.6541, p-val = 0.0012

Test of Moderators (coefficients 2:7):
QM(df = 6) = 12.1623, p-val = 0.0584

Model Results:

                                 estimate       se     zval    pval     ci.lb     ci.ub
intrcpt                          -32.1249  70.6610  -0.4546  0.6494  -170.6179  106.3681
SettingHD and CB                  33.3209  35.6049   0.9359  0.3493   -36.4635  103.1053
SettingHome-based                 50.0147  29.9046   1.6725  0.0944    -8.5973  108.6266   .
SettingTelerehabilitation         64.2706  42.8851   1.4987  0.1340   -19.7827  148.3239
Supervisionalternate supervision  33.8476  29.6194   1.1428  0.2531   -24.2052   91.9005
Supervisionsupervised             91.2670  32.0569   2.8470  0.0044    28.4367  154.0973  **
FEV_1                             -0.3176   1.2101  -0.2624  0.7930    -2.6893    2.0542

---
Signif. codes:  0 '***' 0.001 '**' 0.01 '*' 0.05 '.' 0.1 ' ' 1
```

**Six minute walking test (6MWT) adjusted to) supervision (reference: unsupervised), setting (reference: community-based) and Duration of the maintenance PR programme**

```
> rvmeta_sup_setting_time2 <- metareg(rvmeta_final2,Supervision+Setting+Time_MP)
> rvmeta_sup_setting_time2

Mixed-Effects Model (k = 15; tau^2 estimator: DL)

tau^2 (estimated amount of residual heterogeneity):     779.6215 (SE = 651.8188)
tau (square root of estimated tau^2 value):             27.9217
I^2 (residual heterogeneity / unaccounted variability): 69.99%
H^2 (unaccounted variability / sampling variability):   3.33
R^2 (amount of heterogeneity accounted for):            0.00%

Test for Residual Heterogeneity:
QE(df = 8) = 26.6613, p-val = 0.0008

Test of Moderators (coefficients 2:7):
QM(df = 6) = 12.5903, p-val = 0.0500

Model Results:

                                  estimate       se     zval    pval      ci.lb     ci.ub
intrcpt                           -38.8483  36.4573  -1.0656  0.2866  -110.3033   32.6067
Supervisionalternate supervision   36.8710  26.9761   1.3668  0.1717   -16.0013   89.7432
Supervisionsupervised              89.1990  31.2207   2.8571  0.0043    28.0077  150.3904  **
SettingHD and CB                   33.1213  32.0474   1.0335  0.3014   -29.6904   95.9330
SettingHome-based                  57.3548  27.0478   2.1205  0.0340     4.3420  110.3675   *
SettingTelerehabilitation          67.7563  42.0154   1.6127  0.1068   -14.5924  150.1049
Time_MP                            -0.9846   1.4680  -0.6707  0.5024    -3.8619    1.8927

---
Signif. codes:  0 ‘***’ 0.001 ‘**’ 0.01 ‘*’ 0.05 ‘.’ 0.1 ‘ ’ 1
```

# OUTCOME: Modified Medical Research Council (mMRC)

## All studies:

| Study or Subgroup | MRP Mean | MRP SD | MRP Total | Control Mean | Control SD | Control Total | Weight | Mean Difference IV, Random, 95% CI |
|---|---|---|---|---|---|---|---|---|
| Li, Yi. 2018 | -1 | 0.89 | 65 | 1.2 | 0.82 | 69 | 33.6% | -2.20 [-2.49, -1.91] |
| Souza, Y. 2018 | -2 | 1 | 25 | -1 | 1 | 25 | 32.2% | -1.00 [-1.55, -0.45] |
| Van Wetering, C.R. 2010 | -0.3 | 0.1 | 102 | 0.1 | 0.1 | 97 | 34.2% | -0.40 [-0.43, -0.37] |
| **Total (95% CI)** | | | **192** | | | **191** | **100.0%** | **-1.20 [-2.50, 0.10]** |

Heterogeneity: Tau² = 1.29; Chi² = 150.64, df = 2 (P < 0.00001); I² = 99%
Test for overall effect: Z = 1.80 (P = 0.07)

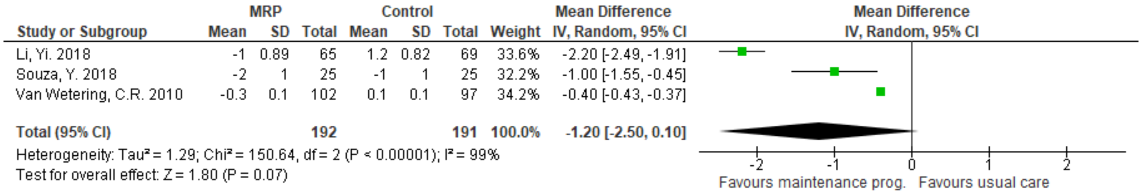


## <u>Sensitive analysis, excluding Li Yi 2018 efect:</u>

| Study or Subgroup | MRP Mean | MRP SD | MRP Total | Control Mean | Control SD | Control Total | Weight | Mean Difference IV, Random, 95% CI |
|---|---|---|---|---|---|---|---|---|
| Li, Yi. 2018 | -1 | 0.89 | 65 | 1.2 | 0.82 | 69 | 0.0% | -2.20 [-2.49, -1.91] |
| Souza, Y. 2018 | -2 | 1 | 25 | -1 | 1 | 25 | 38.9% | -1.00 [-1.55, -0.45] |
| Van Wetering, C.R. 2010 | -0.3 | 0.1 | 102 | 0.1 | 0.1 | 97 | 61.1% | -0.40 [-0.43, -0.37] |
| **Total (95% CI)** | | | **127** | | | **122** | **100.0%** | **-0.63 [-1.21, -0.06]** |

Heterogeneity: Tau² = 0.14; Chi² = 4.49, df = 1 (P = 0.03); I² = 78%
Test for overall effect: Z = 2.17 (P = 0.03)

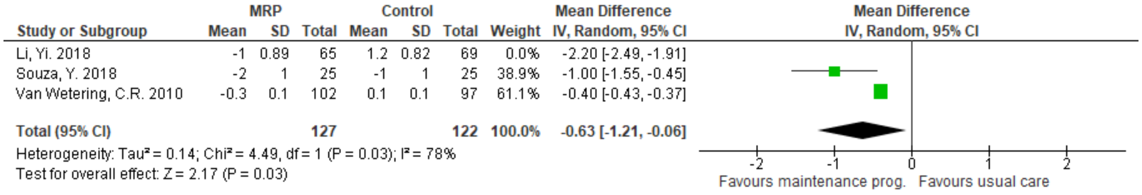

# OUTCOME: Health Related Quality of life

## All studies

| Study or Subgroup | MRP Mean | MRP SD | MRP Total | Control Mean | Control SD | Control Total | Weight | Mean Difference IV, Random, 95% CI |
|---|---|---|---|---|---|---|---|---|
| Cruz, J. 2016 | -7.5 | 14.8 | 16 | -8 | 11.4 | 16 | 20.8% | 0.50 [-8.65, 9.65] |
| Jiménez-Reguera, B. 2020 | -3.7 | 11.6 | 20 | -2.7 | 44.9 | 24 | 7.3% | -1.00 [-19.67, 17.67] |
| Spencer, L.M. 2010 | 3 | 14.1 | 24 | -3 | 16.7 | 24 | 22.0% | 6.00 [-2.74, 14.74] |
| Van Wetering, C.R. 2010 | -3.9 | 1.1 | 102 | 0.3 | 1 | 97 | 49.9% | -4.20 [-4.49, -3.91] |
| **Total (95% CI)** | | | **162** | | | **161** | **100.0%** | **-0.75 [-6.22, 4.73]** |

Heterogeneity: Tau² = 15.62; Chi² = 6.34, df = 3 (P = 0.10); I² = 53%
Test for overall effect: Z = 0.27 (P = 0.79)

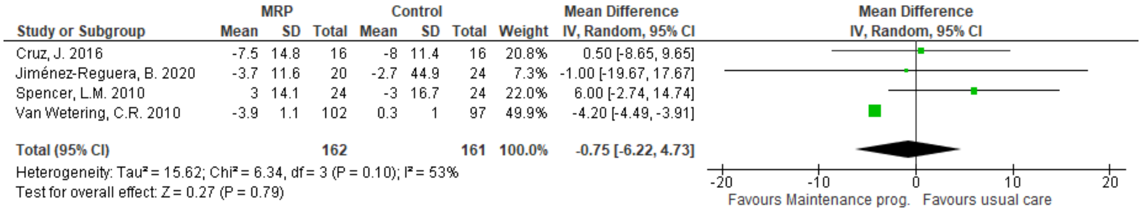


<u>Sensitive analysis, excluding Spencer LM 2010 efect:</u>

| Study or Subgroup | MRP Mean | MRP SD | MRP Total | Control Mean | Control SD | Control Total | Weight | Mean Difference IV, Random, 95% CI |
|---|---|---|---|---|---|---|---|---|
| Cruz, J. 2016 | -7.5 | 14.8 | 16 | -8 | 11.4 | 16 | 0.1% | 0.50 [-8.65, 9.65] |
| Jiménez-Reguera, B. 2020 | -3.7 | 11.6 | 20 | -2.7 | 44.9 | 24 | 0.0% | -1.00 [-19.67, 17.67] |
| Spencer, L.M. 2010 | 3 | 14.1 | 24 | -3 | 16.7 | 24 | 0.0% | 6.00 [-2.74, 14.74] |
| Van Wetering, C.R. 2010 | -3.9 | 1.1 | 102 | 0.3 | 1 | 97 | 99.9% | -4.20 [-4.49, -3.91] |
| **Total (95% CI)** | | | **138** | | | **137** | **100.0%** | **-4.19 [-4.49, -3.90]** |

Heterogeneity: Tau² = 0.00; Chi² = 1.12, df = 2 (P = 0.57); I² = 0%
Test for overall effect: Z = 28.19 (P < 0.00001)

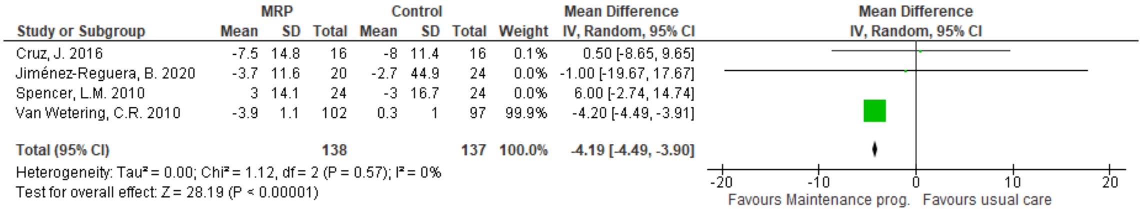


<u>Subgroup analysis according to the main characteristics of the studies:</u>

## Duration time of the maintenance PR programme

| Study or Subgroup | MRP Mean | MRP SD | MRP Total | Control Mean | Control SD | Control Total | Weight | Mean Difference IV, Random, 95% CI |
|---|---|---|---|---|---|---|---|---|
| **11.1.1 Up to 12 months** | | | | | | | | |
| Cruz, J. 2016 | -7.5 | 14.8 | 16 | -8 | 11.4 | 16 | 20.8% | 0.50 [-8.65, 9.65] |
| **Subtotal (95% CI)** | | | **16** | | | **16** | **20.8%** | **0.50 [-8.65, 9.65]** |
| Heterogeneity: Not applicable | | | | | | | | |
| Test for overall effect: Z = 0.11 (P = 0.91) | | | | | | | | |
| **11.1.2 > 12 months** | | | | | | | | |
| Jiménez-Reguera, B. 2020 | -3.7 | 11.6 | 20 | -2.7 | 44.9 | 24 | 7.3% | -1.00 [-19.67, 17.67] |
| Spencer, L.M. 2010 | 3 | 14.1 | 24 | -3 | 16.7 | 24 | 22.0% | 6.00 [-2.74, 14.74] |
| Van Wetering, C.R. 2010 | -3.9 | 1.1 | 102 | 0.3 | 1 | 97 | 49.9% | -4.20 [-4.49, -3.91] |
| **Subtotal (95% CI)** | | | **146** | | | **145** | **79.2%** | **-0.53 [-8.14, 7.08]** |
| Heterogeneity: Tau² = 27.24; Chi² = 5.33, df = 2 (P = 0.07); I² = 62% | | | | | | | | |
| Test for overall effect: Z = 0.14 (P = 0.89) | | | | | | | | |
| **Total (95% CI)** | | | **162** | | | **161** | **100.0%** | **-0.75 [-6.22, 4.73]** |

Heterogeneity: Tau² = 15.62; Chi² = 6.34, df = 3 (P = 0.10); I² = 53%
Test for overall effect: Z = 0.27 (P = 0.79)
Test for subgroup differences: Chi² = 0.03, df = 1 (P = 0.87), I² = 0%

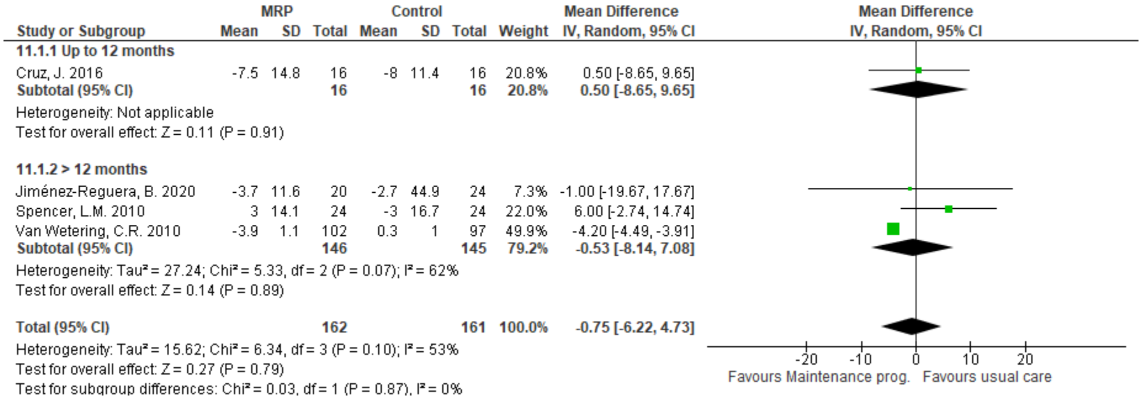

## Duration time of the initial PR programme

| Study or Subgroup | MRP Mean | MRP SD | MRP Total | Control Mean | Control SD | Control Total | Weight | Mean Difference IV, Random, 95% CI |
|---|---|---|---|---|---|---|---|---|
| **13.1.1 8 weeks** | | | | | | | | |
| Jiménez-Reguera, B. 2020 | -3.7 | 11.6 | 20 | -2.7 | 44.9 | 24 | 7.3% | -1.00 [-19.67, 17.67] |
| Spencer, L.M. 2010 | 3 | 14.1 | 24 | -3 | 16.7 | 24 | 22.0% | 6.00 [-2.74, 14.74] |
| **Subtotal (95% CI)** | | | **44** | | | **48** | **29.3%** | **4.74 [-3.18, 12.66]** |
| Heterogeneity: Tau² = 0.00; Chi² = 0.44, df = 1 (P = 0.51); I² = 0% | | | | | | | | |
| Test for overall effect: Z = 1.17 (P = 0.24) | | | | | | | | |
| **13.1.2 12 weeks** | | | | | | | | |
| Cruz, J. 2016 | -7.5 | 14.8 | 16 | -8 | 11.4 | 16 | 20.8% | 0.50 [-8.65, 9.65] |
| Van Wetering, C.R. 2010 | -3.9 | 1.1 | 102 | 0.3 | 1 | 97 | 49.9% | -4.20 [-4.49, -3.91] |
| **Subtotal (95% CI)** | | | **118** | | | **113** | **70.7%** | **-4.17 [-4.92, -3.41]** |
| Heterogeneity: Tau² = 0.13; Chi² = 1.01, df = 1 (P = 0.31); I² = 1% | | | | | | | | |
| Test for overall effect: Z = 10.80 (P < 0.00001) | | | | | | | | |
| **Total (95% CI)** | | | **162** | | | **161** | **100.0%** | **-0.75 [-6.22, 4.73]** |
| Heterogeneity: Tau² = 15.62; Chi² = 6.34, df = 3 (P = 0.10); I² = 53% | | | | | | | | |
| Test for overall effect: Z = 0.27 (P = 0.79) | | | | | | | | |
| Test for subgroup differences: Chi² = 4.82, df = 1 (P = 0.03), I² = 79.2% | | | | | | | | |

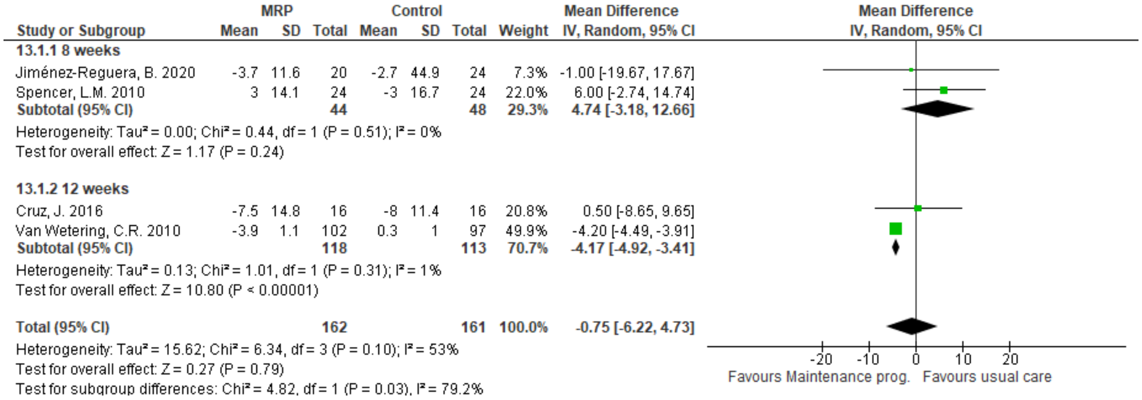


## Severity of COPD

| Study or Subgroup | MRP Mean | MRP SD | MRP Total | Control Mean | Control SD | Control Total | Weight | Mean Difference IV, Random, 95% CI |
|---|---|---|---|---|---|---|---|---|
| **12.1.1 Mild to severe** | | | | | | | | |
| Cruz, J. 2016 | -7.5 | 14.8 | 16 | -8 | 11.4 | 16 | 20.8% | 0.50 [-8.65, 9.65] |
| Jiménez-Reguera, B. 2020 | -3.7 | 11.6 | 20 | -2.7 | 44.9 | 24 | 7.3% | -1.00 [-19.67, 17.67] |
| Spencer, L.M. 2010 | 3 | 14.1 | 24 | -3 | 16.7 | 24 | 22.0% | 6.00 [-2.74, 14.74] |
| Van Wetering, C.R. 2010 | -3.9 | 1.1 | 102 | 0.3 | 1 | 97 | 49.9% | -4.20 [-4.49, -3.91] |
| **Subtotal (95% CI)** | | | **162** | | | **161** | **100.0%** | **-0.75 [-6.22, 4.73]** |
| Heterogeneity: Tau² = 15.62; Chi² = 6.34, df = 3 (P = 0.10); I² = 53% | | | | | | | | |
| Test for overall effect: Z = 0.27 (P = 0.79) | | | | | | | | |
| **12.1.2 Moderate to Severe** | | | | | | | | |
| **Subtotal (95% CI)** | | | **0** | | | **0** | | **Not estimable** |
| Heterogeneity: Not applicable | | | | | | | | |
| Test for overall effect: Not applicable | | | | | | | | |
| **Total (95% CI)** | | | **162** | | | **161** | **100.0%** | **-0.75 [-6.22, 4.73]** |
| Heterogeneity: Tau² = 15.62; Chi² = 6.34, df = 3 (P = 0.10); I² = 53% | | | | | | | | |
| Test for overall effect: Z = 0.27 (P = 0.79) | | | | | | | | |
| Test for subgroup differences: Not applicable | | | | | | | | |

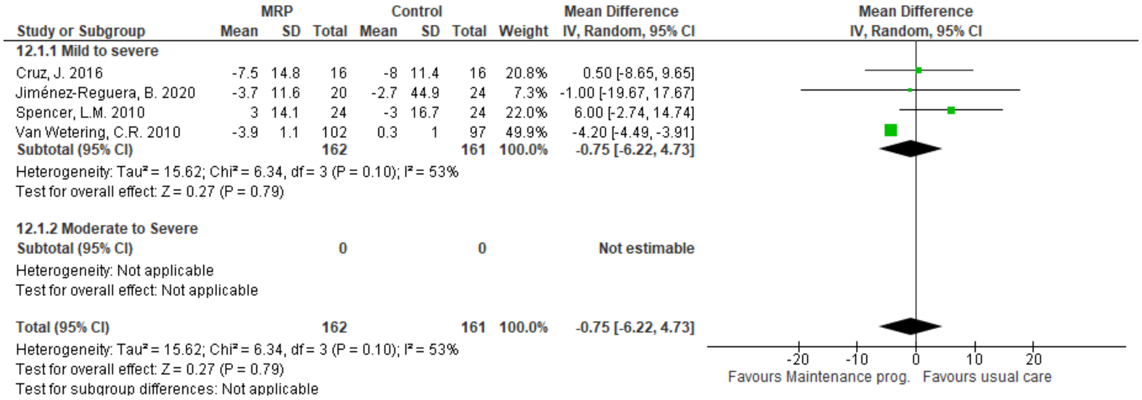


## Professional supervision

| Study or Subgroup | MRP Mean | MRP SD | MRP Total | Control Mean | Control SD | Control Total | Weight | Mean Difference IV, Random, 95% CI |
|---|---|---|---|---|---|---|---|---|
| **10.2.1 Supervised** | | | | | | | | |
| Cruz, J. 2016 | -7.5 | 14.8 | 16 | -8 | 11.4 | 16 | 20.8% | 0.50 [-8.65, 9.65] |
| **Subtotal (95% CI)** | | | **16** | | | **16** | **20.8%** | **0.50 [-8.65, 9.65]** |
| Heterogeneity: Not applicable | | | | | | | | |
| Test for overall effect: Z = 0.11 (P = 0.91) | | | | | | | | |
| **10.2.2 Unsupervised** | | | | | | | | |
| Jiménez-Reguera, B. 2020 | -3.7 | 11.6 | 20 | -2.7 | 44.9 | 24 | 7.3% | -1.00 [-19.67, 17.67] |
| **Subtotal (95% CI)** | | | **20** | | | **24** | **7.3%** | **-1.00 [-19.67, 17.67]** |
| Heterogeneity: Not applicable | | | | | | | | |
| Test for overall effect: Z = 0.10 (P = 0.92) | | | | | | | | |
| **10.2.3 Alternate supervision** | | | | | | | | |
| Spencer, L.M. 2010 | 3 | 14.1 | 24 | -3 | 16.7 | 24 | 22.0% | 6.00 [-2.74, 14.74] |
| Van Wetering, C.R. 2010 | -3.9 | 1.1 | 102 | 0.3 | 1 | 97 | 49.9% | -4.20 [-4.49, -3.91] |
| **Subtotal (95% CI)** | | | **126** | | | **121** | **71.8%** | **-0.07 [-9.89, 9.74]** |
| Heterogeneity: Tau² = 42.06; Chi² = 5.22, df = 1 (P = 0.02); I² = 81% | | | | | | | | |
| Test for overall effect: Z = 0.01 (P = 0.99) | | | | | | | | |
| **Total (95% CI)** | | | **162** | | | **161** | **100.0%** | **-0.75 [-6.22, 4.73]** |
| Heterogeneity: Tau² = 15.62; Chi² = 6.34, df = 3 (P = 0.10); I² = 53% | | | | | | | | |
| Test for overall effect: Z = 0.27 (P = 0.79) | | | | | | | | |
| Test for subgroup differences: Chi² = 0.02, df = 2 (P = 0.99), I² = 0% | | | | | | | | |

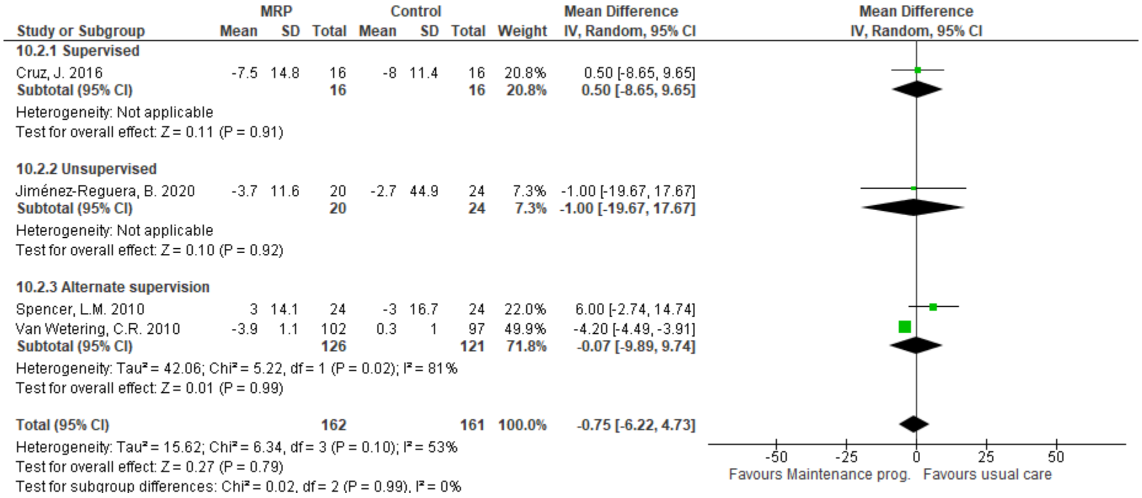

## Setting

| Study or Subgroup | MRP Mean | MRP SD | MRP Total | Control Mean | Control SD | Control Total | Weight | Mean Difference IV, Random, 95% CI |
|---|---|---|---|---|---|---|---|---|
| **15.1.1 Home-based (HB)** | | | | | | | | |
| Van Wetering, C.R. 2010 | -3.9 | 1.1 | 102 | 0.3 | 1 | 97 | 49.9% | -4.20 [-4.49, -3.91] |
| **Subtotal (95% CI)** | | | **102** | | | **97** | **49.9%** | **-4.20 [-4.49, -3.91]** |
| Heterogeneity: Not applicable | | | | | | | | |
| Test for overall effect: Z = 28.21 (P < 0.00001) | | | | | | | | |
| **15.1.2 Community-based (CB)** | | | | | | | | |
| **Subtotal (95% CI)** | | | **0** | | | **0** | | **Not estimable** |
| Heterogeneity: Not applicable | | | | | | | | |
| Test for overall effect: Not applicable | | | | | | | | |
| **15.1.3 Alternate HB/CB** | | | | | | | | |
| Cruz, J. 2016 | -7.5 | 14.8 | 16 | -8 | 11.4 | 16 | 20.8% | 0.50 [-8.65, 9.65] |
| Spencer, L.M. 2010 | 3 | 14.1 | 24 | -3 | 16.7 | 24 | 22.0% | 6.00 [-2.74, 14.74] |
| **Subtotal (95% CI)** | | | **40** | | | **40** | **42.8%** | **3.38 [-2.95, 9.70]** |
| Heterogeneity: Tau² = 0.00; Chi² = 0.73, df = 1 (P = 0.39); I² = 0% | | | | | | | | |
| Test for overall effect: Z = 1.05 (P = 0.30) | | | | | | | | |
| **15.1.4 Telerehabilitation** | | | | | | | | |
| Jiménez-Reguera, B. 2020 | -3.7 | 11.6 | 20 | -2.7 | 44.9 | 24 | 7.3% | -1.00 [-19.67, 17.67] |
| **Subtotal (95% CI)** | | | **20** | | | **24** | **7.3%** | **-1.00 [-19.67, 17.67]** |
| Heterogeneity: Not applicable | | | | | | | | |
| Test for overall effect: Z = 0.10 (P = 0.92) | | | | | | | | |
| **Total (95% CI)** | | | **162** | | | **161** | **100.0%** | **-0.75 [-6.22, 4.73]** |
| Heterogeneity: Tau² = 15.62; Chi² = 6.34, df = 3 (P = 0.10); I² = 53% | | | | | | | | |
| Test for overall effect: Z = 0.27 (P = 0.79) | | | | | | | | |
| Test for subgroup differences: Chi² = 5.61, df = 2 (P = 0.06), I² = 64.4% | | | | | | | | |

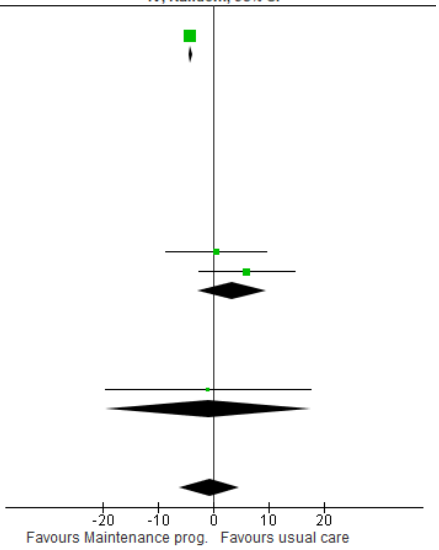


## Risk of Bias

| Study or Subgroup | MRP Mean | MRP SD | MRP Total | Control Mean | Control SD | Control Total | Weight | Mean Difference IV, Random, 95% CI | Risk of Bias A | B | C | D | E | F | G |
|---|---|---|---|---|---|---|---|---|---|---|---|---|---|---|---|
| **8.1.1 Low risk of bias** | | | | | | | | | | | | | | | |
| Jiménez-Reguera, B. 2020 | -3.7 | 11.6 | 20 | -2.7 | 44.9 | 24 | 7.3% | -1.00 [-19.67, 17.67] | + | + | − | + | + | + | + |
| Van Wetering, C.R. 2010 | -3.9 | 1.1 | 102 | 0.3 | 1 | 97 | 49.9% | -4.20 [-4.49, -3.91] | + | + | − | + | + | + | + |
| **Subtotal (95% CI)** | | | **122** | | | **121** | **57.2%** | **-4.20 [-4.49, -3.91]** | | | | | | | |
| Heterogeneity: Tau² = 0.00; Chi² = 0.11, df = 1 (P = 0.74); I² = 0% | | | | | | | | | | | | | | | |
| Test for overall effect: Z = 28.20 (P < 0.00001) | | | | | | | | | | | | | | | |
| **8.1.2 Uncertain and High risk of bias** | | | | | | | | | | | | | | | |
| Cruz, J. 2016 | -7.5 | 14.8 | 16 | -8 | 11.4 | 16 | 20.8% | 0.50 [-8.65, 9.65] | + | + | − | − | + | + | + |
| Spencer, L.M. 2010 | 3 | 14.1 | 24 | -3 | 16.7 | 24 | 22.0% | 6.00 [-2.74, 14.74] | + | + | − | ? | + | + | + |
| **Subtotal (95% CI)** | | | **40** | | | **40** | **42.8%** | **3.38 [-2.95, 9.70]** | | | | | | | |
| Heterogeneity: Tau² = 0.00; Chi² = 0.73, df = 1 (P = 0.39); I² = 0% | | | | | | | | | | | | | | | |
| Test for overall effect: Z = 1.05 (P = 0.30) | | | | | | | | | | | | | | | |
| **Total (95% CI)** | | | **162** | | | **161** | **100.0%** | **-0.75 [-6.22, 4.73]** | | | | | | | |
| Heterogeneity: Tau² = 15.62; Chi² = 6.34, df = 3 (P = 0.10); I² = 53% | | | | | | | | | | | | | | | |
| Test for overall effect: Z = 0.27 (P = 0.79) | | | | | | | | | | | | | | | |
| Test for subgroup differences: Chi² = 5.50, df = 1 (P = 0.02), I² = 81.8% | | | | | | | | | | | | | | | |

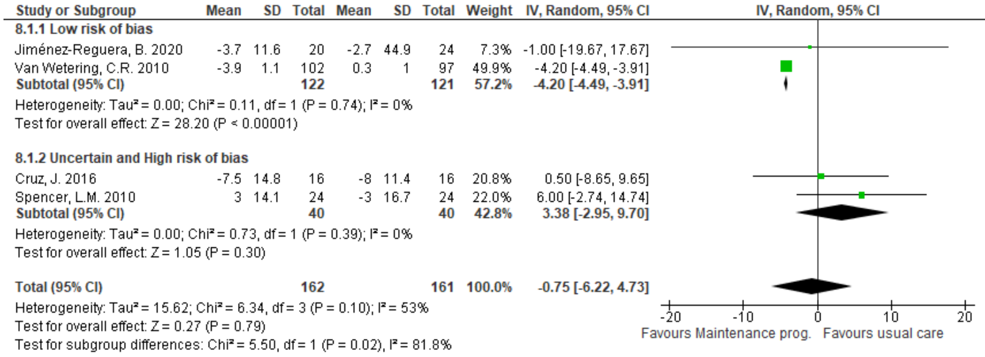


Risk of bias legend
(A) Random sequence generation (selection bias)
(B) Allocation concealment (selection bias)
(C) Blinding of participants and personnel (performance bias)
(D) Blinding of outcome assessment (detection bias)
(E) Incomplete outcome data (attrition bias)
(F) Selective reporting (reporting bias)
(G) Other bias

# OUTCOME: Chronic respiratory disease questionnaire (CRDQ)

## All studies:

| Study or Subgroup | MPR Mean | MPR SD | MPR Total | Usual Care Mean | Usual Care SD | Usual Care Total | Weight | Mean Difference IV, Random, 95% CI |
|---|---|---|---|---|---|---|---|---|
| Butler, S. 2020 | 0.1 | 1.1 | 49 | 0 | 1 | 48 | 27.9% | 0.10 [-0.32, 0.52] |
| du Moulin, M. 2008 | 0.4 | 0.7 | 10 | -0.3 | 0.8 | 10 | 16.4% | 0.70 [0.04, 1.36] |
| Galdiz, J.B. 2021 | -0.17 | 1.4 | 48 | 0 | 1.4 | 46 | 20.0% | -0.17 [-0.74, 0.40] |
| Guell, M-R., 2017 | -0.5 | 1.4 | 68 | -0.27 | 1.4 | 70 | 25.0% | -0.23 [-0.70, 0.24] |
| Román, M. 2013 | 0.44 | 1.8 | 32 | 0 | 1.8 | 33 | 10.7% | 0.44 [-0.44, 1.32] |
| **Total (95% CI)** | | | **207** | | | **207** | **100.0%** | **0.10 [-0.22, 0.42]** |

Heterogeneity: Tau² = 0.05; Chi² = 6.49, df = 4 (P = 0.17); I² = 38%
Test for overall effect: Z = 0.60 (P = 0.55)

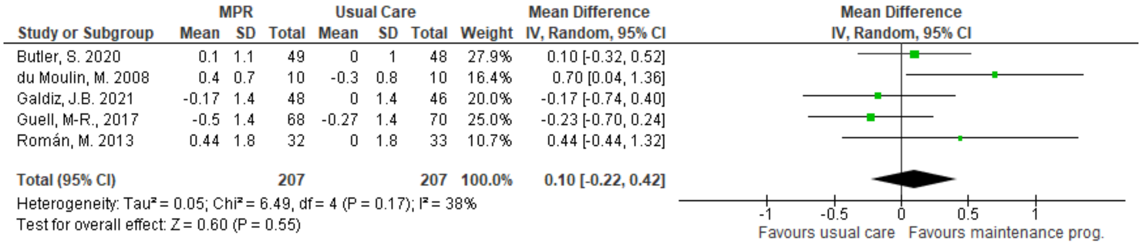


Subgroup analysis according to the main characteristics of the studies:

## Duration time of the maintenance PR programme

| Study or Subgroup | MPR Mean | MPR SD | MPR Total | Usual Care Mean | Usual Care SD | Usual Care Total | Weight | Mean Difference IV, Random, 95% CI |
|---|---|---|---|---|---|---|---|---|
| **9.1.1 Up to 6 months** | | | | | | | | |
| du Moulin, M. 2008 | 0.4 | 0.7 | 10 | -0.3 | 0.8 | 10 | 16.4% | 0.70 [0.04, 1.36] |
| **Subtotal (95% CI)** | | | **10** | | | **10** | **16.4%** | **0.70 [0.04, 1.36]** |
| Heterogeneity: Not applicable | | | | | | | | |
| Test for overall effect: Z = 2.08 (P = 0.04) | | | | | | | | |
| **9.1.2 12 months** | | | | | | | | |
| Butler, S. 2020 | 0.1 | 1.1 | 49 | 0 | 1 | 48 | 27.9% | 0.10 [-0.32, 0.52] |
| Galdiz, J.B. 2021 | -0.17 | 1.4 | 48 | 0 | 1.4 | 46 | 20.0% | -0.17 [-0.74, 0.40] |
| **Subtotal (95% CI)** | | | **97** | | | **94** | **47.9%** | **0.00 [-0.33, 0.34]** |
| Heterogeneity: Tau² = 0.00; Chi² = 0.57, df = 1 (P = 0.45); I² = 0% | | | | | | | | |
| Test for overall effect: Z = 0.03 (P = 0.98) | | | | | | | | |
| **9.1.3 More than 12 months** | | | | | | | | |
| Guell, M-R., 2017 | -0.5 | 1.4 | 68 | -0.27 | 1.4 | 70 | 25.0% | -0.23 [-0.70, 0.24] |
| Román, M. 2013 | 0.44 | 1.8 | 32 | 0 | 1.8 | 33 | 10.7% | 0.44 [-0.44, 1.32] |
| **Subtotal (95% CI)** | | | **100** | | | **103** | **35.7%** | **-0.00 [-0.62, 0.62]** |
| Heterogeneity: Tau² = 0.10; Chi² = 1.75, df = 1 (P = 0.19); I² = 43% | | | | | | | | |
| Test for overall effect: Z = 0.00 (P = 1.00) | | | | | | | | |
| **Total (95% CI)** | | | **207** | | | **207** | **100.0%** | **0.10 [-0.22, 0.42]** |

Heterogeneity: Tau² = 0.05; Chi² = 6.49, df = 4 (P = 0.17); I² = 38%
Test for overall effect: Z = 0.60 (P = 0.55)
Test for subgroup differences: Chi² = 3.57, df = 2 (P = 0.17), I² = 44.0%

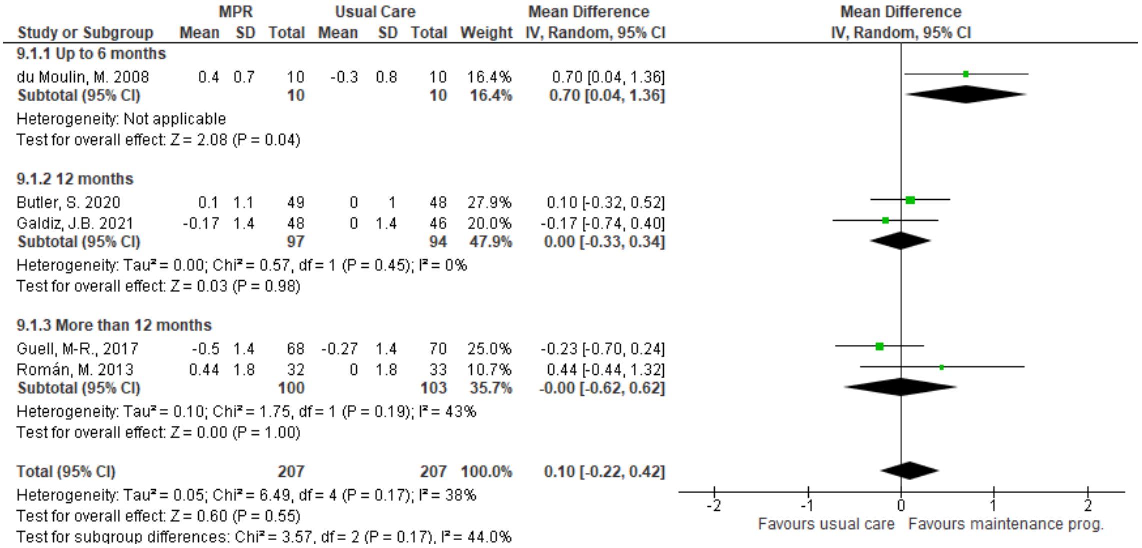

## Duration time of the initial PR programme

| Study or Subgroup | MPR Mean | MPR SD | MPR Total | Usual Care Mean | Usual Care SD | Usual Care Total | Weight | Mean Difference IV, Random, 95% CI |
|---|---|---|---|---|---|---|---|---|
| **9.2.1 3-6 weeks** | | | | | | | | |
| du Moulin, M. 2008 | 0.4 | 0.7 | 10 | -0.3 | 0.8 | 10 | 23.8% | 0.70 [0.04, 1.36] |
| **Subtotal (95% CI)** | | | **10** | | | **10** | **23.8%** | **0.70 [0.04, 1.36]** |
| Heterogeneity: Not applicable | | | | | | | | |
| Test for overall effect: Z = 2.08 (P = 0.04) | | | | | | | | |
| **9.2.2 8 weeks** | | | | | | | | |
| Galdiz, J.B. 2021 | -0.17 | 1.4 | 48 | 0 | 1.4 | 46 | 27.4% | -0.17 [-0.74, 0.40] |
| Guell, M-R., 2017 | -0.5 | 1.4 | 68 | -0.27 | 1.4 | 70 | 31.7% | -0.23 [-0.70, 0.24] |
| **Subtotal (95% CI)** | | | **116** | | | **116** | **59.0%** | **-0.21 [-0.57, 0.15]** |
| Heterogeneity: Tau$^2$ = 0.00; Chi$^2$ = 0.03, df = 1 (P = 0.87); I$^2$ = 0% | | | | | | | | |
| Test for overall effect: Z = 1.12 (P = 0.26) | | | | | | | | |
| **9.2.3 12 weeks** | | | | | | | | |
| Román, M. 2013 | 0.44 | 1.8 | 32 | 0 | 1.8 | 33 | 17.2% | 0.44 [-0.44, 1.32] |
| **Subtotal (95% CI)** | | | **32** | | | **33** | **17.2%** | **0.44 [-0.44, 1.32]** |
| Heterogeneity: Not applicable | | | | | | | | |
| Test for overall effect: Z = 0.99 (P = 0.32) | | | | | | | | |
| **Total (95% CI)** | | | **158** | | | **159** | **100.0%** | **0.12 [-0.33, 0.58]** |
| Heterogeneity: Tau$^2$ = 0.11; Chi$^2$ = 6.46, df = 3 (P = 0.09); I$^2$ = 54% | | | | | | | | |
| Test for overall effect: Z = 0.53 (P = 0.60) | | | | | | | | |
| Test for subgroup differences: Chi$^2$ = 6.44, df = 2 (P = 0.04), I$^2$ = 68.9% | | | | | | | | |

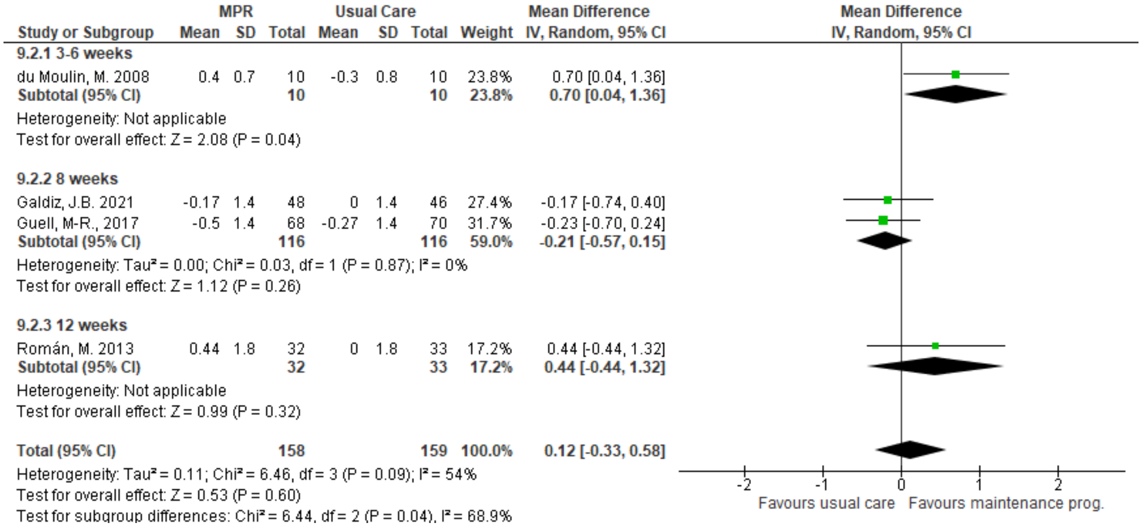


## Severity of COPD

| Study or Subgroup | MPR Mean | MPR SD | MPR Total | Usual Care Mean | Usual Care SD | Usual Care Total | Weight | Mean Difference IV, Random, 95% CI |
|---|---|---|---|---|---|---|---|---|
| **9.3.1 Mild to severe** | | | | | | | | |
| Román, M. 2013 | 0.44 | 1.8 | 32 | 0 | 1.8 | 33 | 8.9% | 0.44 [-0.44, 1.32] |
| **Subtotal (95% CI)** | | | **32** | | | **33** | **8.9%** | **0.44 [-0.44, 1.32]** |
| Heterogeneity: Not applicable | | | | | | | | |
| Test for overall effect: Z = 0.99 (P = 0.32) | | | | | | | | |
| **9.3.2 Moderate to severe** | | | | | | | | |
| Butler, S. 2020 | 0.1 | 1.1 | 49 | 0 | 1 | 48 | 38.8% | 0.10 [-0.32, 0.52] |
| Galdiz, J.B. 2021 | -0.17 | 1.4 | 48 | 0 | 1.4 | 46 | 21.2% | -0.17 [-0.74, 0.40] |
| Guell, M-R., 2017 | -0.5 | 1.4 | 68 | -0.27 | 1.4 | 70 | 31.1% | -0.23 [-0.70, 0.24] |
| **Subtotal (95% CI)** | | | **165** | | | **164** | **91.1%** | **-0.08 [-0.35, 0.20]** |
| Heterogeneity: Tau$^2$ = 0.00; Chi$^2$ = 1.20, df = 2 (P = 0.55); I$^2$ = 0% | | | | | | | | |
| Test for overall effect: Z = 0.54 (P = 0.59) | | | | | | | | |
| **Total (95% CI)** | | | **197** | | | **197** | **100.0%** | **-0.03 [-0.29, 0.23]** |
| Heterogeneity: Tau$^2$ = 0.00; Chi$^2$ = 2.42, df = 3 (P = 0.49); I$^2$ = 0% | | | | | | | | |
| Test for overall effect: Z = 0.22 (P = 0.82) | | | | | | | | |
| Test for subgroup differences: Chi$^2$ = 1.21, df = 1 (P = 0.27), I$^2$ = 17.6% | | | | | | | | |

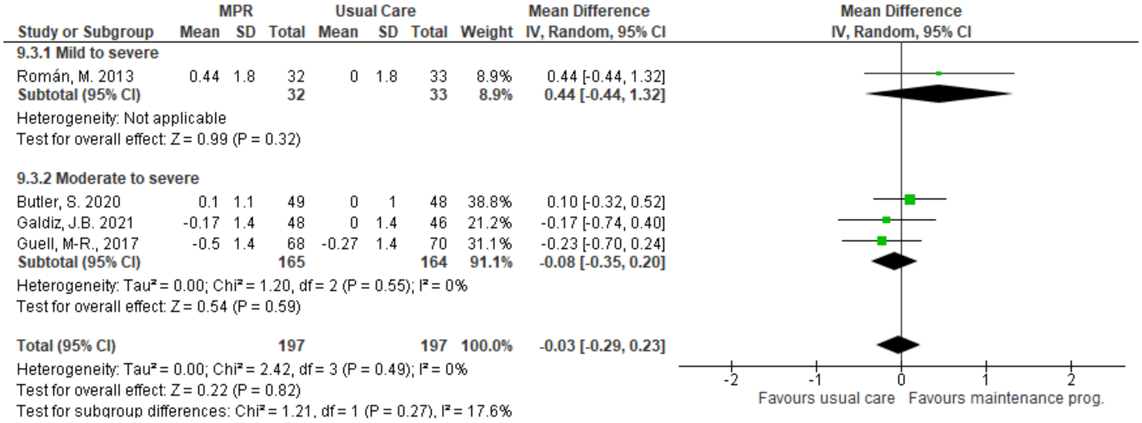


## Professional supervision

| Study or Subgroup | MPR Mean | MPR SD | MPR Total | Usual Care Mean | Usual Care SD | Usual Care Total | Weight | Mean Difference IV, Random, 95% CI |
|---|---|---|---|---|---|---|---|---|
| **9.4.1 Supervised** | | | | | | | | |
| Román, M. 2013 | 0.44 | 1.8 | 32 | 0 | 1.8 | 33 | 10.7% | 0.44 [-0.44, 1.32] |
| **Subtotal (95% CI)** | | | **32** | | | **33** | **10.7%** | **0.44 [-0.44, 1.32]** |
| Heterogeneity: Not applicable | | | | | | | | |
| Test for overall effect: Z = 0.99 (P = 0.32) | | | | | | | | |
| **9.4.2 Unsupervised** | | | | | | | | |
| du Moulin, M. 2008 | 0.4 | 0.7 | 10 | -0.3 | 0.8 | 10 | 16.4% | 0.70 [0.04, 1.36] |
| Galdiz, J.B. 2021 | -0.17 | 1.4 | 48 | 0 | 1.4 | 46 | 20.0% | -0.17 [-0.74, 0.40] |
| **Subtotal (95% CI)** | | | **58** | | | **56** | **36.4%** | **0.25 [-0.60, 1.10]** |
| Heterogeneity: Tau$^2$ = 0.28; Chi$^2$ = 3.85, df = 1 (P = 0.05); I$^2$ = 74% | | | | | | | | |
| Test for overall effect: Z = 0.57 (P = 0.57) | | | | | | | | |
| **9.4.3 Alternate supervision** | | | | | | | | |
| Butler, S. 2020 | 0.1 | 1.1 | 49 | 0 | 1 | 48 | 27.9% | 0.10 [-0.32, 0.52] |
| Guell, M-R., 2017 | -0.5 | 1.4 | 68 | -0.27 | 1.4 | 70 | 25.0% | -0.23 [-0.70, 0.24] |
| **Subtotal (95% CI)** | | | **117** | | | **118** | **52.9%** | **-0.05 [-0.37, 0.27]** |
| Heterogeneity: Tau$^2$ = 0.00; Chi$^2$ = 1.06, df = 1 (P = 0.30); I$^2$ = 6% | | | | | | | | |
| Test for overall effect: Z = 0.29 (P = 0.77) | | | | | | | | |
| **Total (95% CI)** | | | **207** | | | **207** | **100.0%** | **0.10 [-0.22, 0.42]** |
| Heterogeneity: Tau$^2$ = 0.05; Chi$^2$ = 6.49, df = 4 (P = 0.17); I$^2$ = 38% | | | | | | | | |
| Test for overall effect: Z = 0.60 (P = 0.55) | | | | | | | | |
| Test for subgroup differences: Chi$^2$ = 1.32, df = 2 (P = 0.52), I$^2$ = 0% | | | | | | | | |

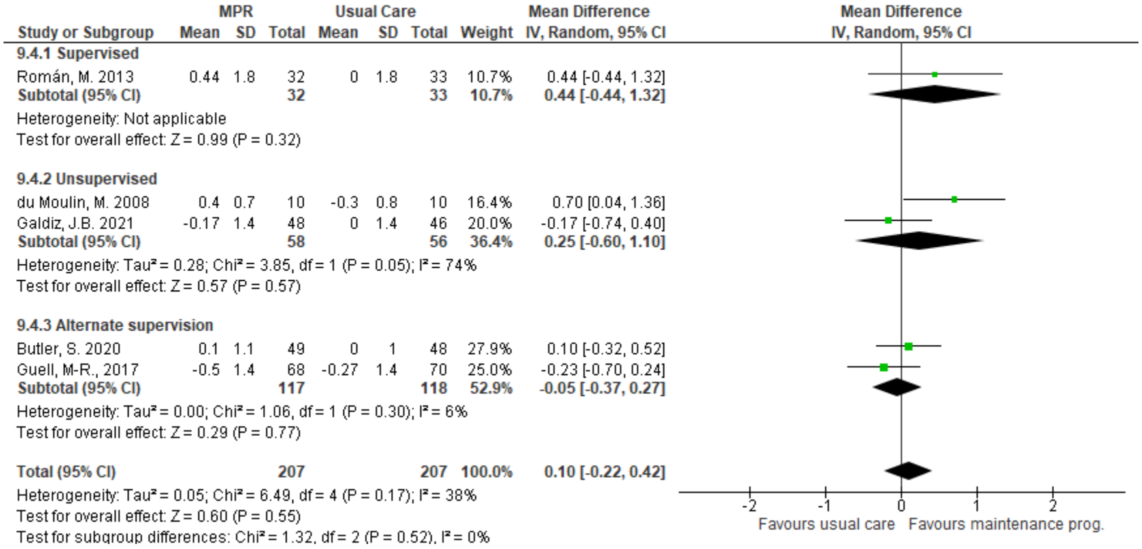

## Setting

| Study or Subgroup | MPR Mean | MPR SD | MPR Total | Usual Care Mean | Usual Care SD | Usual Care Total | Weight | Mean Difference IV, Random, 95% CI |
|---|---|---|---|---|---|---|---|---|
| **9.6.1 Home-based** | | | | | | | | |
| du Moulin, M. 2008 | 0.4 | 0.7 | 10 | -0.3 | 0.8 | 10 | 16.4% | 0.70 [0.04, 1.36] |
| Guell, M-R., 2017 | -0.5 | 1.4 | 68 | -0.27 | 1.4 | 70 | 25.0% | -0.23 [-0.70, 0.24] |
| **Subtotal (95% CI)** | | | **78** | | | **80** | **41.4%** | **0.20 [-0.70, 1.11]** |
| Heterogeneity: Tau² = 0.35; Chi² = 5.09, df = 1 (P = 0.02); I² = 80% | | | | | | | | |
| Test for overall effect: Z = 0.44 (P = 0.66) | | | | | | | | |
| **9.6.2 Community-based** | | | | | | | | |
| Butler, S. 2020 | 0.1 | 1.1 | 49 | 0 | 1 | 48 | 27.9% | 0.10 [-0.32, 0.52] |
| Román, M. 2013 | 0.44 | 1.8 | 32 | 0 | 1.8 | 33 | 10.7% | 0.44 [-0.44, 1.32] |
| **Subtotal (95% CI)** | | | **81** | | | **81** | **38.6%** | **0.16 [-0.21, 0.54]** |
| Heterogeneity: Tau² = 0.00; Chi² = 0.47, df = 1 (P = 0.49); I² = 0% | | | | | | | | |
| Test for overall effect: Z = 0.85 (P = 0.40) | | | | | | | | |
| **9.6.3 Alternate HB/CM** | | | | | | | | |
| **Subtotal (95% CI)** | | | **0** | | | **0** | | **Not estimable** |
| Heterogeneity: Not applicable | | | | | | | | |
| Test for overall effect: Not applicable | | | | | | | | |
| **9.6.4 Telerehabilitation** | | | | | | | | |
| Galdiz, J.B. 2021 | -0.17 | 1.4 | 48 | 0 | 1.4 | 46 | 20.0% | -0.17 [-0.74, 0.40] |
| **Subtotal (95% CI)** | | | **48** | | | **46** | **20.0%** | **-0.17 [-0.74, 0.40]** |
| Heterogeneity: Not applicable | | | | | | | | |
| Test for overall effect: Z = 0.59 (P = 0.56) | | | | | | | | |
| **Total (95% CI)** | | | **207** | | | **207** | **100.0%** | **0.10 [-0.22, 0.42]** |
| Heterogeneity: Tau² = 0.05; Chi² = 6.49, df = 4 (P = 0.17); I² = 38% | | | | | | | | |
| Test for overall effect: Z = 0.60 (P = 0.55) | | | | | | | | |
| Test for subgroup differences: Chi² = 1.01, df = 2 (P = 0.60), I² = 0% | | | | | | | | |

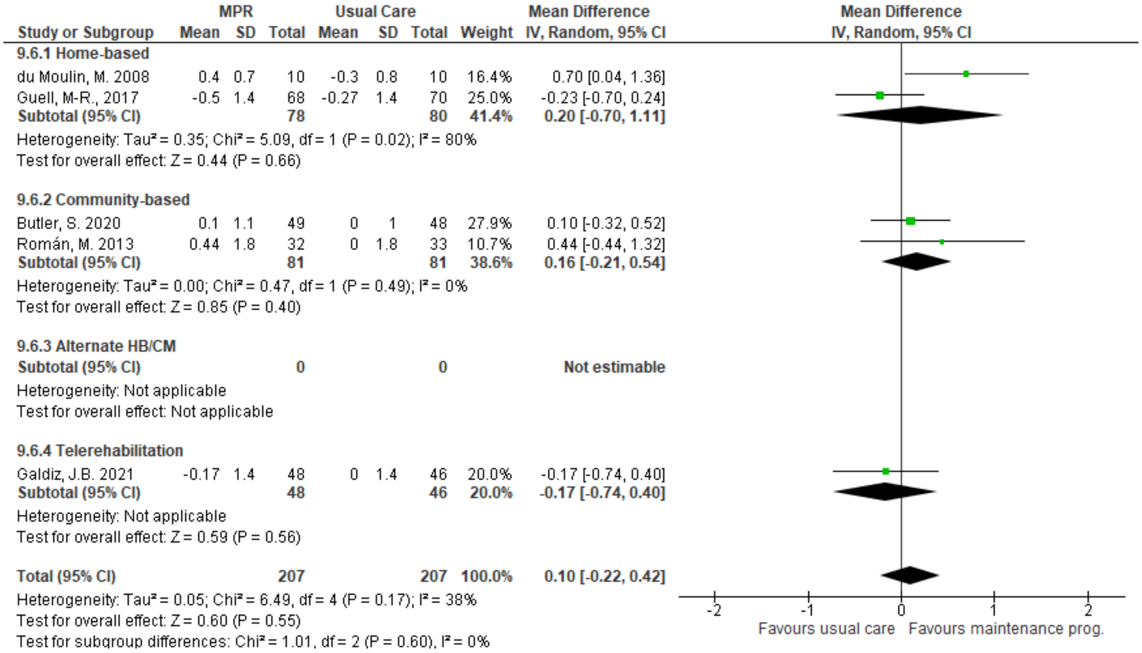


## Risk of Bias

| Study or Subgroup | MPR Mean | MPR SD | MPR Total | Usual Care Mean | Usual Care SD | Usual Care Total | Weight | Mean Difference IV, Random, 95% CI | A | B | C | D | E | F | G |
|---|---|---|---|---|---|---|---|---|---|---|---|---|---|---|---|
| **9.5.1 Uncertain risk of bias** | | | | | | | | | | | | | | | |
| Galdiz, J.B. 2021 | -0.17 | 1.4 | 48 | 0 | 1.4 | 46 | 20.0% | -0.17 [-0.74, 0.40] | + | + | − | ? | + | + | + |
| Guell, M-R., 2017 | -0.5 | 1.4 | 68 | -0.27 | 1.4 | 70 | 25.0% | -0.23 [-0.70, 0.24] | + | ? | − | + | + | + | + |
| Román, M. 2013 | 0.44 | 1.8 | 32 | 0 | 1.8 | 33 | 10.7% | 0.44 [-0.44, 1.32] | + | + | − | ? | + | + | + |
| **Subtotal (95% CI)** | | | **148** | | | **149** | **55.7%** | **-0.11 [-0.45, 0.22]** | | | | | | | |
| Heterogeneity: Tau² = 0.00; Chi² = 1.81, df = 2 (P = 0.40); I² = 0% | | | | | | | | | | | | | | | |
| Test for overall effect: Z = 0.66 (P = 0.51) | | | | | | | | | | | | | | | |
| **9.5.2 Low risk of bias** | | | | | | | | | | | | | | | |
| Butler, S. 2020 | 0.1 | 1.1 | 49 | 0 | 1 | 48 | 27.9% | 0.10 [-0.32, 0.52] | + | + | − | + | + | + | + |
| du Moulin, M. 2008 | 0.4 | 0.7 | 10 | -0.3 | 0.8 | 10 | 16.4% | 0.70 [0.04, 1.36] | + | + | − | + | + | + | + |
| **Subtotal (95% CI)** | | | **59** | | | **58** | **44.3%** | **0.34 [-0.23, 0.92]** | | | | | | | |
| Heterogeneity: Tau² = 0.10; Chi² = 2.27, df = 1 (P = 0.13); I² = 56% | | | | | | | | | | | | | | | |
| Test for overall effect: Z = 1.17 (P = 0.24) | | | | | | | | | | | | | | | |
| **Total (95% CI)** | | | **207** | | | **207** | **100.0%** | **0.10 [-0.22, 0.42]** | | | | | | | |
| Heterogeneity: Tau² = 0.05; Chi² = 6.49, df = 4 (P = 0.17); I² = 38% | | | | | | | | | | | | | | | |
| Test for overall effect: Z = 0.60 (P = 0.55) | | | | | | | | | | | | | | | |
| Test for subgroup differences: Chi² = 1.80, df = 1 (P = 0.18), I² = 44.3% | | | | | | | | | | | | | | | |

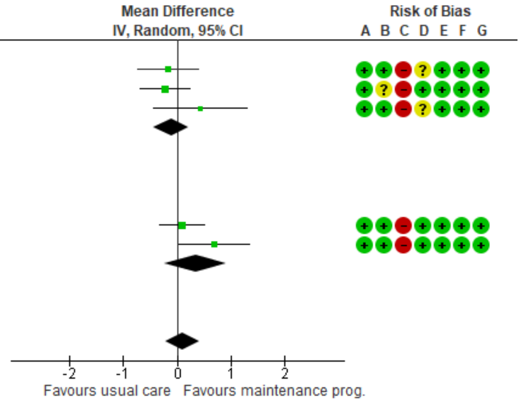


Risk of bias legend
(A) Random sequence generation (selection bias)
(B) Allocation concealment (selection bias)
(C) Blinding of participants and personnel (performance bias)
(D) Blinding of outcome assessment (detection bias)
(E) Incomplete outcome data (attrition bias)
(F) Selective reporting (reporting bias)
(G) Other bias
